# A Palladium-Catalyzed Cascade Double Annulation Strategy for Modular Access to Dihydrocyclopenta[*b*]chromenes

**DOI:** 10.1021/acs.orglett.5c01856

**Published:** 2025-07-28

**Authors:** Yanan Liu, Pui Ying Choy, Qiang Tang, Mengdi Wu, Yangzilin Kong, Yongjia Shang, Fuk Yee Kwong, Xinwei He

**Affiliations:** † Key Laboratory of Functional Molecular Solids, Ministry of Education, Anhui Laboratory of Molecule-Based Materials (State Key Laboratory Cultivation Base), College of Chemistry and Materials Science, Anhui Normal University, Wuhu 241000, P. R. China; ‡ Department of Chemistry and State Key Laboratory of Synthetic Chemistry, The Chinese University of Hong Kong, Shatin, New Territories, Hong Kong 999077, P.R. China; ⊥ The Translational Research Institute for Neurological Disorders & Interdisciplinary Research Center of Neuromedicine and Chemical Biology of Wannan Medical College and Anhui Normal University, Department of Neurosurgery, The First Affiliated Hospital of Wannan Medical College (Yijishan Hospital of Wannan Medical College), Wuhu 241001, P.R. China

## Abstract

The cyclopentachromene skeleton is a key structural motif in pharmaceutical and fluorescent materials. We here introduce a Pd-promoted double ring-closure strategy for tackling the modular synthesis of densely functionalized dihydrocyclopenta­[*b*]­chromenes, which proceeds simply from two reaction partners with high diversity. This scheme features a reaction sequence beginning with 1,6-conjugate addition of *ortho*-alkynyl quinone methide, followed by annulation and migratory insertion. This approach is compatible with functional groups, providing a streamlined pathway to access complex five-membered ring-fused chromenes.

## Graphical abstract

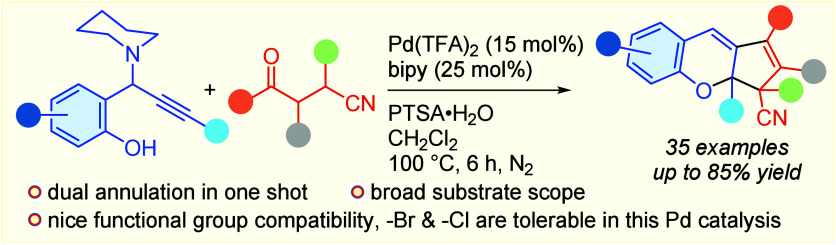

Heterocyclic compounds are essential in the pharmaceutical industry, as evidenced by the fact that more than 70% of marketed drugs contain at least one heterocyclic moiety.^1^ Among these, the cyclopentene-fused *O*-heterocycles have emerged as privileged scaffolds due to their broad bioactivity profile (Scheme 1a).^2^ Cyclopentachromenes, in particular, exhibit remarkable pharmacological properties and unique therapeutic potential.^3^ Notably, one of the cyclopenta­[*b*]­chromene isomers where the cyclopentene subunit is fused to the C2 and C3 positions of the chromene skeleton displays a distinctive fluorescence property, making it valuable for bioimaging.^4^ Despite the rich utility of these compounds, synthetic access remains limited.^5^ Recent advances, such as Pd­(II)-catalyzed denitrative annulation, has enabled the synthesis of dihydrocyclopenta­[*b*]­chromenes, yet faced persistent regiocontrol challenges (Scheme 1b).^6^ In fact, the limited synthetic protocol underscores the need to develop a new, selective, and modular synthetic scheme that can effectively produce structurally diverse entities favorable for pharmaceutical and material investigations.

**Scheme 1:**
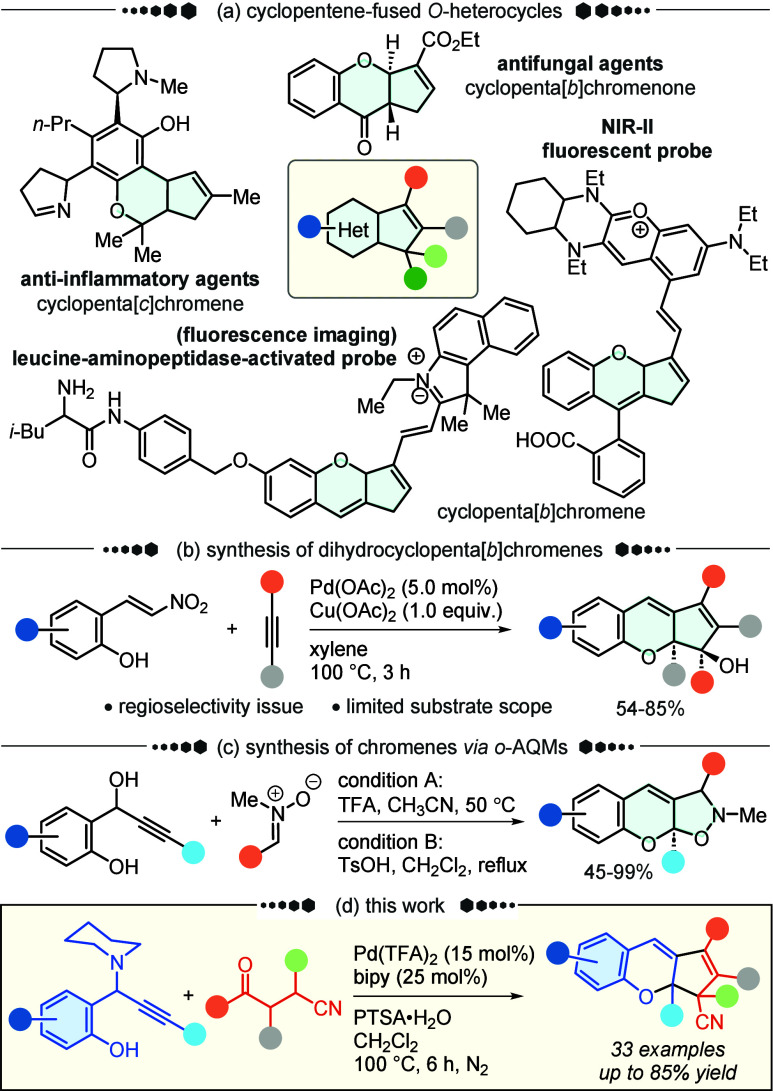
Synthetic Strategies for Assembling Cyclopenta­[*b*]­chromene Derivatives

*ortho*-Alkynyl quinone methide (*o*-AQM) has been proven to be a versatile precursor for constructing complex polycyclic architectures,^7^ including fused-chromene derivatives.^8^ Recently, catalytic annulations of *o*-AQM, generated *in situ* from *o*-hydroxyphenyl propargylamines and propargylic alcohols, were developed for the synthesis of five-membered heterocycle-fused chromenes (Scheme 1c).^9,10^ Nevertheless, a catalytic profile for reaching the five-membered carbon ring-fused chromene skeleton remains elusive, likely due to the challenge in controlling the selectivity of *o*-AQM. Building on our effort in palladium-catalyzed annulation^11^ and our prior works on *o*-AQM chemistry for heterocycle assembly,^12^ herein we introduce a general and efficient pathway for the modular synthesis of dihydrocyclopenta­[*b*]­chromenes **3,** making the first instance of such a preparation (Scheme 1d).

At the outset of our work, we investigated the influence of various representative palladium metal sources, ligands, additives, and solvents on the annulation reaction between propargylamine **1a** and β-ketomalononitrile **2a** (Table 1). Screening of the Pd source revealed that Pd­(TFA)_2_ was the catalyst of choice for the ring-closure process, affording product **3aa** in 40% yield (Table 1, entries 1–4). Shifting the ligand from 1,10-phen to bipy resulted in a slightly higher yield of **3aa** (entry 2 vs entry 6), while PPh_3_ did not promote the reaction (entry 5). Other bipyridine-type ligands were tested, and bipy offered the best performance (entries 6–10). The effect of the catalyst loading, reaction temperature, and time were also investigated (entry 6 vs entries 11–18). Next, different solvents and additives were surveyed (see the Supporting Information for details, Table S2, entries 17–23). With the conditions of dichloromethane as the solvent and *p*-toluenesulfonic acid monohydrate (PTSA·H_2_O) as the additive, the desired product **3aa** was obtained in optimized yield (standard conditions, entry 17). The structure of **3aa** was unambiguously characterized by single-crystal X-ray analysis (Scheme 2a, CCDC 2431986, see the Supporting Information for details).

**Scheme 2:**
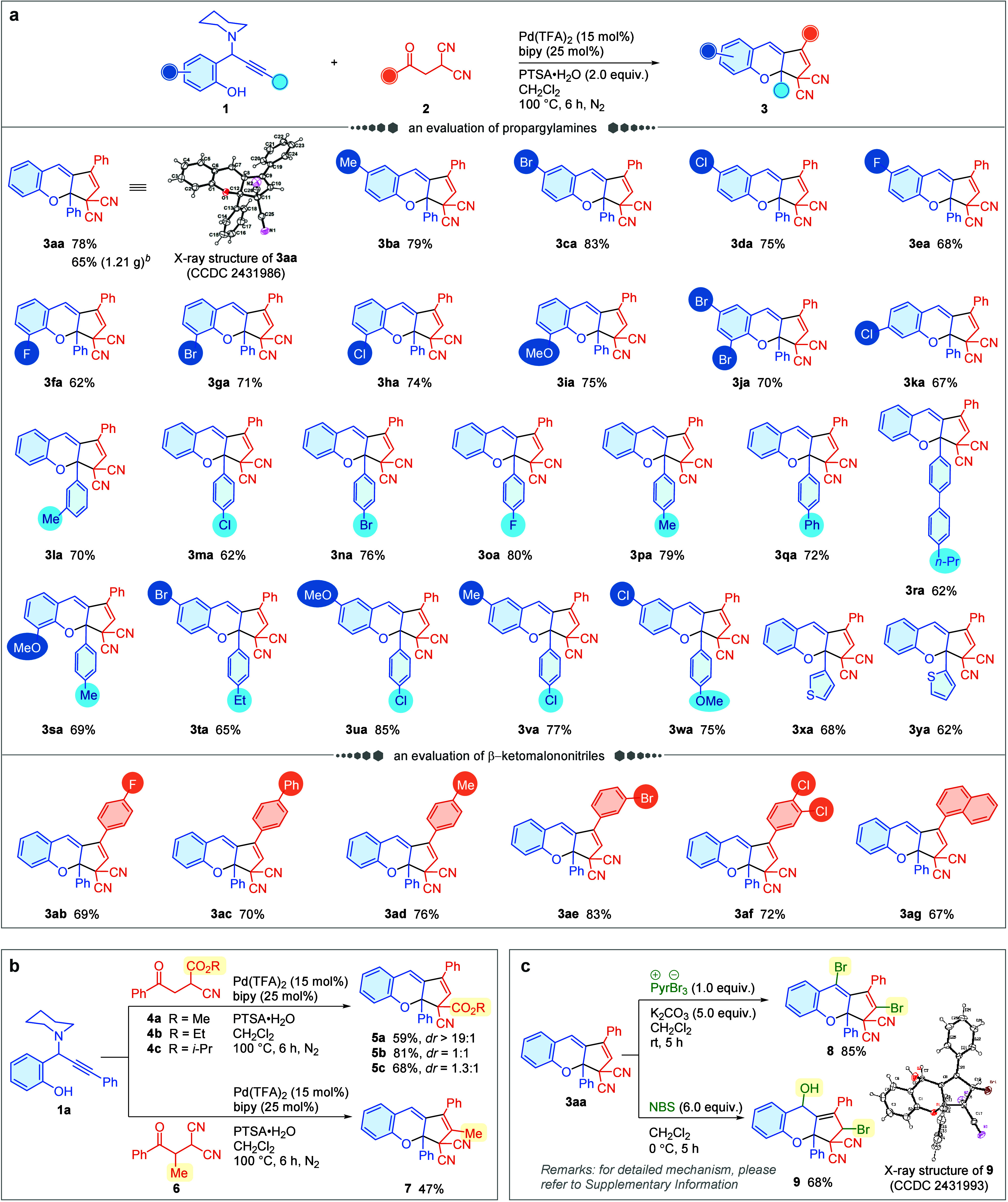
Substrate Scope and Further Transformation^a^

a Reaction conditions: propargylamines **1** (0.15 mmol), **2** or **4** or **6** (0.1 mmol), Pd­(TFA)_2_ (15 mol %), 2,2′-bipyridine (bipy) (25 mol %), and PTSA·H_2_O (2.0 equiv) in dichloromethane (2 mL) at 100 °C under a nitrogen atmosphere for 6 h. Isolated yields were reported.
b Gram-scale synthesis.

**Table 1:**
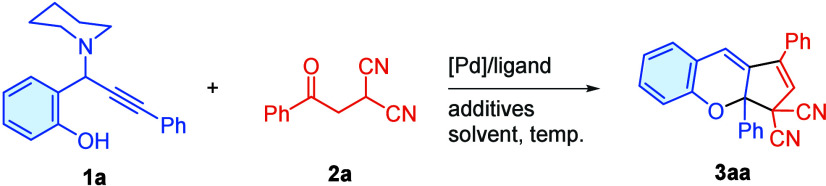
Optimization of Reaction Conditions^a^

entry	Pd catalyst (mol %)	ligand (mol %)	additives	yield (%)^b^
1	Pd(OAc)_2_ (15)	1,10-phen (30)	PTSA·H_2_O	24
2	Pd(TFA)_2_ (15)	1,10-phen (30)	PTSA·H_2_O	40
3	PdCl_2_(PPh_3_)_2_ (15)	1,10-phen (30)	PTSA·H_2_O	n.r.
4	[Pd(MeCN)_4_][BF_4_]_2_ (15)	1,10-phen (30)	PTSA·H_2_O	32
5	Pd(TFA)_2_ (15)	PPh_3_ (30)	PTSA·H_2_O	n.r.
6	Pd(TFA)_2_ (15)	bipy (30)	PTSA·H_2_O	49
7	Pd(TFA)_2_ (15)	Bphen (30)	PTSA·H_2_O	14
8	Pd(TFA)_2_ (15)	DMPHEN (30)	PTSA·H_2_O	n.r.
9	Pd(TFA)_2_ (15)	**L1** (30)	PTSA·H_2_O	23
10	Pd(TFA)_2_ (15)	**L2** (30)	PTSA·H_2_O	n.r.
11^c^	Pd(TFA)_2_ (15)	bipy (30)	PTSA·H_2_O	32
12^d^	Pd(TFA)_2_ (15)	bipy (30)	PTSA·H_2_O	60
13^e^	Pd(TFA)_2_ (15)	bipy (30)	PTSA·H_2_O	51
14^d^	Pd(TFA)_2_ (5)	bipy (30)	PTSA·H_2_O	trace
15^d^	Pd(TFA)_2_ (10)	bipy (30)	PTSA·H_2_O	37
16^d^	Pd(TFA)_2_ (20)	bipy (30)	PTSA·H_2_O	45
17^d^	Pd(TFA)_2_ (15)	bipy (25)	PTSA·H_2_O	78
18^d^	Pd(TFA)_2_ (15)	bipy (15)	PTSA·H_2_O	50
19^d^	Pd(TFA)_2_ (15)	bipy (25)	PivOH	n.r.

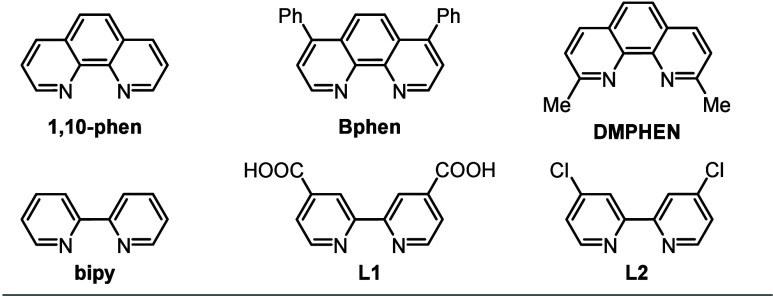

a Reaction conditions: propargylamine **1a** (0.15 mmol), 2-(2-oxo-2-phenylethyl)­malononitrile (**2a**) (0.1 mmol), Pd catalyst (as indicated), ligand (as indicated), and additives (2.0 equiv) in dichloromethane (2 mL) under a nitrogen atmosphere at 100 °C for 12 h.

b Isolated yields. n.r. = no reaction.

c At 80 °C.

d For 6 h.

e For 4 h.

f CHCl_3_ was used instead of CH_2_Cl_2_.

g 1,2-DCE was used instead of CH_2_Cl_2_.

h Toluene was used instead of CH_2_Cl_2_.

With the optimal reaction conditions established, we then studied the generality of this annulation process. As summarized in Scheme 2a, propargylamines **1** bearing substituents locating at the *para*-position (products **3ba-3ea**, **3ta**–**3wa**), *ortho*-position (products **3fa**–**3ia**, **3sa**) and *meta*-position (product **3ka**) to the hydroxyl group were well-tolerated, and the corresponding products were afforded smoothly (52–85% yields). This methodology is also applicable to propargylamines **1**, regardless of whether they possess electron-rich (products **3la**, **3wa**), or electron-deficient (products **3ma**–**3oa**, **3ua**, **3va**) substituents at the alkynyl arene, giving products in satisfactory yields (62–85%). Of particular note is the fact that halo groups, *e.g.*, – Br, −Cl, and −F groups, located at either the phenolic arene or the alkynyl arene, remained intact under these reaction conditions (products **3ca**–**3ha**, **3ja**, **3ka**, **3ma**–**3oa**, **3ua**, **3va**). This beneficial outcome allows the products to undergo further transformations through established cross-coupling strategies at a later stage.^13^ Replacing the phenyl group, which directly associated with the alkynyl segment in **1a**, with a 3- or 2-thienyl substituent gave rise to product **3xa** or **3ya** in 68% or 62% yield, respectively.

The scope of β-ketomalononitrile **2** was further investigated. The annulation reaction proceeded smoothly with substrates **2** bearing a halo group, delivering desired products in good yields (products **3ab**, **3ae**). Particularly noteworthy is that a substrate bearing dichloro groups was successfully applied in this reaction, producing **3af** in 72% yield. This reaction was also found to be compatible with sterically hindered 2-(2-(naphthalen-1-yl)-2-oxoethyl)­malononitrile in which the π-extended structure **3ag** was able to be constructed in 67% yield. The scale-up efficiency of the annulation strategy was demonstrated through a gram-scale experiment, providing **3aa** in a satisfactory yield. In addition, by replacing one of the cyano groups of **2a** with an ester group, substrates **4** were identified as viable coupling partners for this reaction, resulting in the production of compounds **5** in good yields (59–81%) (Scheme 2b). The α-substituted ketone **6** was also determined to be a feasible substrate in this reaction, leading to incorporation of an all-substituted peripheral moiety (Scheme 2b). The potential synthetic applications were further evaluated by the extended transformation of the annulated products (Scheme 2c). Upon treatment of compound **3aa** with pyridinium tribromide in the presence of K_2_CO_3_ and CH_2_Cl_2_ at room temperature for 5 h, the dibromo-substituted product **8** was afforded in 85% yield. Another common brominating reagent, *N*-bromosuccinimide (NBS), was also attempted to treat compound **3aa**, and the hydroxy-bromo-product **9** was obtained in 68% yield (see the Supporting Information for a proposed mechanism). The geometry of **9** was characterized by single crystal X-ray crystallographic analysis (CCDC 2431993, see the Supporting Information for details).

To probe the mechanism of this reaction, a series of control experiments were performed (Scheme 3). When propargylamine **10,** a substrate without an *ortho*-hydroxyl group, was subjected to the standard reaction conditions, the prototypical reaction was completely shut down (Scheme 3a). Not surprisingly, product **3aa** was not observed when the hydroxyl group was altered from the *ortho*-position to the *meta*-position on propargylamine **11**. In addition, substrate **2a** underwent single cyclization with the alkynyl segment only when the hydroxyl group was attached at the *para*-position, affording furan derivative **13** in 46% yield (see the Supporting Information for a proposed mechanism). These results indicated that the *ortho*-hydroxyl group of propargylamine **1a** is crucial in facilitating the annulation reaction through the *in situ* generation of the *o*-AQM key intermediate. Attempt to further simplify the strategy by using a direct three-component one-pot assembly approach were unsuccessful (Scheme 3b).

**Scheme 3:**
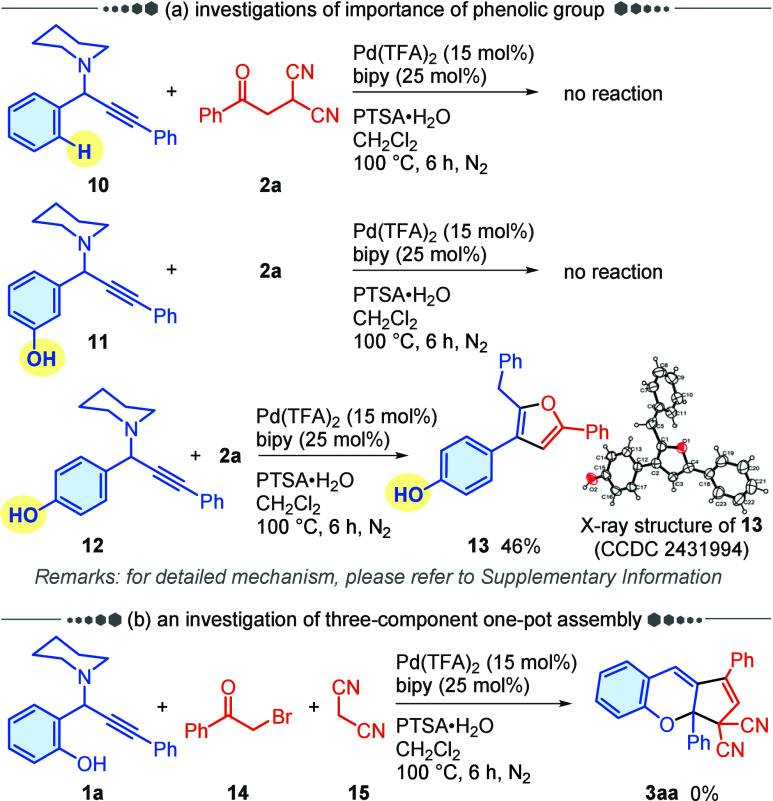
Control Experiment

Based on the literature precedent^9,10,14^ and above experimental observations, a plausible mechanism for the formation of compound **3aa** is proposed (Scheme 4). Initially, propargylamine **1a** binds with the Pd center with the aid of a nitrogen lone pair and a C–C triple bond π-donor coordination, generating intermediate **A**. Subsequent deamination of intermediate **A** leads to the formation of quinone intermediate **B**, which then undergoes 1,6-conjugate addition with **2a** to give intermediate **C**. Then, annulation of intermediate **C** produces intermediate **D**, which further undergoes migratory insertion (formation of intermediate **E**) and protonation to regenerate the Pd catalyst, yielding the intermediate **F**, which was confirmed by GC-MS. Finally, the desired product **3aa** is eventually attained through a facile dehydration process of intermediate **F**.

**Scheme 4:**
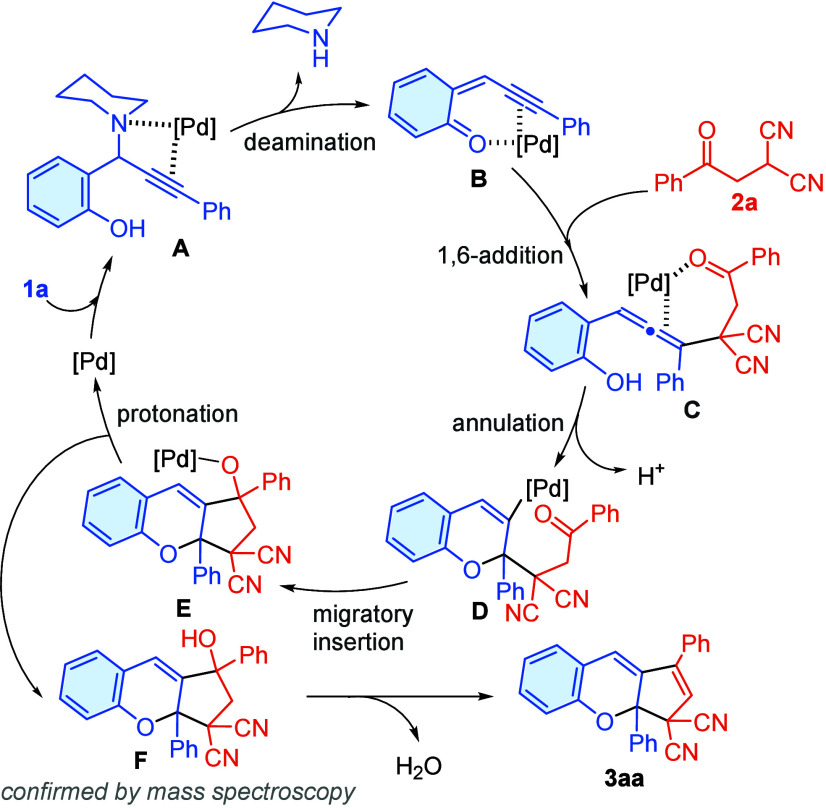
Proposed Mechanism for Product **3aa** Formation

The photophysical properties of dihydrocyclopenta­[*b*]­chromenes **3** and their late-stage functionalized derivatives were systematically investigated (see Supporting Information for details). The majority of these compounds exhibited photoluminescence in the visible spectrum, emitting blue light with emission maxima ranging from 387 to 474 nm and a Stokes shift varying from 59 to 143 nm (for the photophysical property data, see the Supporting Information for details). These findings underscore the potential utility of this class of dihydrocyclopenta­[*b*]­chromenes in bioimaging applications, given their distinctive fluorescence properties.

In conclusion, the cyclopentachromene skeleton represents a unique five-membered ring-fused chromene unit for a variety of pharmaceutical and materials applications. Nevertheless, late-stage modification of this framework by selective functionalization of the arene core and cyclopentyl periphery poses significant synthetic challenges. In this study, we established a Pd-catalyzed dual-ring-closure strategy for the modular assembly of densely functionalized cyclopentachromene systems. This method leverages a cascade sequence involving conjugate addition, annulation and migratory insertion, enabling the direct construction of complex five-membered ring-fused chromenes from simple and diverse precursors. It is worth noting that this versatile palladium catalysis approach effectively accommodates various functional groups, particularly being compatible with −Br and −Cl groups, thereby offering the possibility of more complexity management *via* existing cross-coupling technologies for attaining complex five-membered ring-fused chromenes. It is believed that this method not only unlocks previous limitations for accessing these scaffolds but also provides new opportunities for skeletal manipulation of fluorophore structures, with potential applications in bioimaging and advanced material explorations.

## Supplementary Material



## Data Availability

The data underlying this study are available in the published article, in its Supporting Information, and openly available in ACS chemistry databank at DOI:10.5061/dryad.zs7h44jnd.

## Notes

Experimental details; procedures; crystal data; characterization of compounds; ^1^H, ^13^C, and ^19^F NMR spectra; and characterization data of the products (PDF)
