# Supplementary material for: A Palladium-Catalyzed Cascade Double Annulation Strategy for Modular Access to Dihydrocyclopenta[b]chromenes
Source: Org Lett. 2025 Jul 28;27(31):8405–10. doi: 10.1021/acs.orglett.5c01856 (PMC12340954; doi:10.1021/acs.orglett.5c01856)
Supplement: Supplementary file 1 [file ol5c01856_si_001.pdf]

Supporting Information

A Palladium-Catalyzed Cascade Double Annulation Strategy for Modular Access of Dihydrocyclopenta[*b*]chromenes

Yanan Liu,[a] Pui Ying Choy,[b] Qiang Tang,[a,c] Mengdi Wu,[a] Yangzilin Kong,[a] Yongjia Shang,*[a] Fuk Yee Kwong,*[b] and Xinwei He*[a]

[a]*Key Laboratory of Functional Molecular Solids, Ministry of Education, Anhui Laboratory of Molecule-Based Materials (State Key Laboratory Cultivation Base), College of Chemistry and Materials Science, Anhui Normal University, Wuhu 241000, P. R. China.*

[b]*Department of Chemistry and State Key Laboratory of Synthetic Chemistry, The Chinese University of Hong Kong, Shatin, New Territories, Hong Kong, P. R. China.*

[c]*The Translational Research Institute for Neurological Disorders & Interdisciplinary Research Center of Neuromedicine and Chemical Biology of Wannan Medical College and Anhui Normal University, Department of Neurosurgery, The First Affiliated Hospital of Wannan Medical College (Yijishan Hospital of Wannan Medical College), Wuhu 241001, P.R. China.*

E-mail: shyj@mail.ahnu.edu.cn, fykwong@cuhk.edu.hk, xinweihe@mail.ahnu.edu.cn

Table of contents

1. General Considerations S2
2. General Procedures for Synthesis of Propargylamines **1** S3
3. General Procedures for Synthesis of 2-(2-oxo-2-arylylethyl)malononitriles **2** S3
4. General Procedures for Ligand and Reaction Condition Screenings S4
5. General Procedures for Pd-catalyzed Annulation Reaction S6
6. General Procedures for Gram-scale Synthesis of Compound **3aa** S6
7. General Procedures for Synthesis of Product **8** S7
8. General Procedures for Synthesis of Product **9** S7
9. General Procedures for Synthesis of Product **13** S8
10. X-ray Crystallographic Data of Product **3aa** S9
11. X-ray Crystallographic Data of Product **9** S10
12. X-ray Crystallographic Data of Product **13** S11
13. Characterization Data for All Products S12
14. ^{1}H, ^{13}C, and ^{19}F NMR Spectra S27
15. Proposed Mechanisms for the Generation of Compound **9** and **13** S69
16. GC-MS Spectra for Mechanistic Investigations S70
17. Photophysical Properties of Product **3** and Their Derivatives S71
18. References S72

1. General Considerations

Unless otherwise noted, all reagents and starting materials were purchased from commercial suppliers and used as received without purification. All cascade reactions were performed in a resealable screw-capped Schlenk flask (approx. 20 mL volume) in the presence of Teflon coated magnetic stirrer bar (4.5 mm × 12 mm). Dichloromethane (CH_2Cl_2) was freshly distilled from calcium hydride under nitrogen before use.[1] Thin layer chromatography was conducted on precoated silica gel 60 F_{254} plates. Silica gel (200-300 mesh) was used for column chromatography. Melting points were recorded on an uncorrected instrument. ^{1}H NMR spectra were recorded on a 400 MHz spectrometer. Spectra were referenced internally to the residual proton resonance in $CDCl_3$ (δ 7.26 ppm) or *d*-DMSO (δ 2.50 ppm). ^{13}C NMR spectra were recorded on a 100 MHz spectrometer and the spectra were referenced to $CDCl_3$ (δ 77.0 ppm) or *d*-DMSO (δ 39.5 ppm). Multiplicities are described as s (singlet), d (doublet), t (triplet), q (quartet), or m (multiplet), and the coupling constants (J) are reported in Hertz (Hz). HRMS analysis with a quadrupole time-of-flight mass spectrometer yielded ion mass/charge (m/z) ratios in atomic mass units. High-resolution mass spectra (HRMS) were obtained by quadrupole time-of-flight mass spectrometer yielded ion mass/charge (m/z) ratios in atomic mass units. Compounds described in the literature were characterized by comparison of their ^{1}H, and/or ^{13}C NMR spectra to the previously reported data.

2. General Procedures for Synthesis of Propargylamines 1

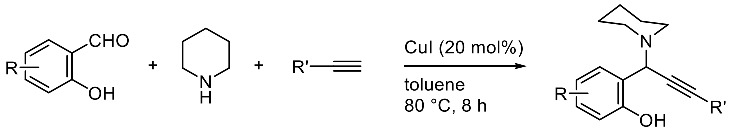

According the reported literature,[2] piperidine (6.5 mmol), aldehyde (5.0 mmol, 1.0 equiv.), acetylene (6.5 mmol), copper(I) iodide (20 mol%) and toluene (10 mL) were added to a 25 mL round-bottom flask equipped with a magnetic stir bar. The mixture was degassed and backfilled with nitrogen, and then stirred in a preheated oil bath at 80 °C for 8 h (monitored by TLC). After the reaction completed (as determined using TLC), the reaction mixture was cooled to room temperature, diluted with dichloromethane (CH_2Cl_2) (10 mL) and filtered through a thin pad of silica gel. The filter cake was washed with CH_2Cl_2, and the combined filtrate was concentrated in vacuum. The crude product was purified by flash column chromatography on silica gel to afford the corresponding propargylamines **1**. All the substrates **1** were known compounds.

3. General Procedures for Synthesis of 2-(2-oxo-2-arylylethyl)malononitriles 2

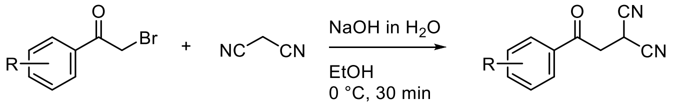

According the reported literature,[3] was added α-bromoacetophenone (10.0 mmol, 1.0 equiv.), malononitrile (0.66 g, 10.0 mmol, 1.0 equiv.) and EtOH (20 mL) were added to an oven-dried 100 mL round bottom flask. NaOH (0.4 g, 10.0 mmol, 1.0 equiv.) in H_2O (20 mL) was added over 5 min. The reaction mixture was stirred for 30 min at 0 °C, then H_2O (20 mL) was added and the colorless residue was filtered off and dried under a constant stream of air. Recrystallization was carried out using EtOH to afford the desired 2-(2-oxo-2-arylylethyl)malononitriles (**2**) which was a known compound.

4. General Procedures for Ligand and Reaction Condition Screenings

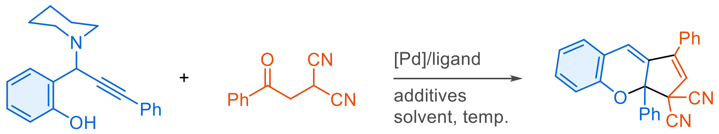

Pd source (mol% indicated in Table S2) and ligand (indicated in Table S1 and S2) were loaded into a Schlenk tube with a Teflon-coated magnetic stir bar. Propargylamine **1** (0.3 mmol), 2-(2-oxo-2-arylylethyl)malononitriles **2** (1.0 equiv.) and additives (2.0 equiv.) were loaded to Schlenk tube, and the system was further evacuated and flushed with nitrogen for three cycles. Solvent (2.0 mL) was added with stirring at room temperature for several minutes. The tube was then placed into a pre-heated oil bath (indicated in Table S2) and stirred for the duration as indicated. The reaction tube was allowed to cool to room temperature. CH_2Cl_2 (~3 x 10 mL), and brine were added for extraction. The organic layer was purified by flash column chromatography with ethyl acetate and petroleum ether as the elution solvent to give desired products **3**.

Table S1. Ligand screening[a]

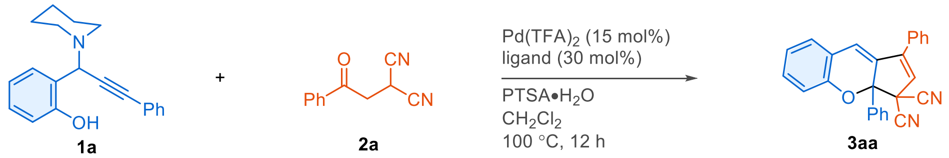

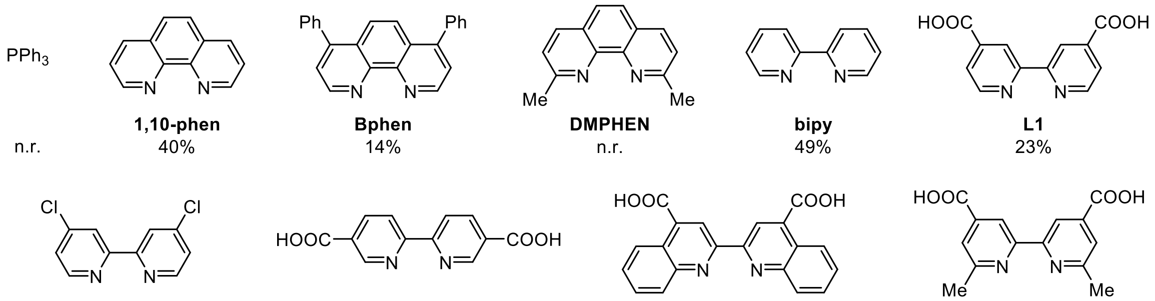

[a]Reaction conditions: Propargylamine **1a** (0.15 mmol), 2-(2-oxo-2-phenylethyl)malononitrile (**2a**) (0.1 mmol), $Pd(TFA)_2$ (15 mol%), ligand (30 mol%), and *p*-toluenesulfonic acid monohydrate ($PTSA \bullet H_2O$) (2.0 equiv.) in dichloromethane (2 mL) under nitrogen atmosphere at 100 °C for 12 h. Isolated yield is reported. n.r. = no reaction.

Table S2. Optimization of reaction conditions[a]

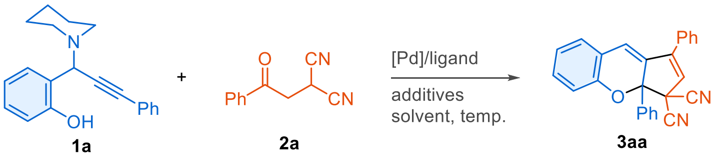

entry	Pd catalyst (mol %)	ligand (mol%)	solvent	additives	temp (°C)	yield (%)[b]
1	$Pd(OAc)_2$ (15)	1,10-phen (30)	CH_2Cl_2	PTSA•H_2O	100	24
2	$Pd(TFA)_2$ (15)	1,10-phen (30)	CH_2Cl_2	PTSA•H_2O	100	40
3	$PdCl_2(PPh_3)_2$ (15)	1,10-phen (30)	CH_2Cl_2	PTSA•H_2O	100	n.r.
4	$[Pd(MeCN)_4][BF_4]_2$ (15)	1,10-phen (30)	CH_2Cl_2	PTSA•H_2O	100	32
5	$Pd(TFA)_2$ (15)	---	CH_2Cl_2	PTSA•H_2O	100	n.r.
6	---	bipy (30)	CH_2Cl_2	PTSA•H_2O	100	n.r.
7	$Pd(TFA)_2$ (15)	bipy (30)	CH_2Cl_2	---	100	n.r.
8	$Pd(TFA)_2$ (15)	bipy (30)	CH_2Cl_2	PTSA•H_2O	80	32
9[c]	$Pd(TFA)_2$ (15)	bipy (30)	CH_2Cl_2	PTSA•H_2O	100	60
10[d]	$Pd(TFA)_2$ (15)	bipy (30)	CH_2Cl_2	PTSA•H_2O	100	51
11[c]	$Pd(TFA)_2$ (5)	bipy (30)	CH_2Cl_2	PTSA•H_2O	100	trace
12[c]	$Pd(TFA)_2$ (10)	bipy (30)	CH_2Cl_2	PTSA•H_2O	100	37
13[c]	$Pd(TFA)_2$ (20)	bipy (30)	CH_2Cl_2	PTSA•H_2O	100	45
14[c]	$Pd(TFA)_2$ (15)	bipy (25)	CH_2Cl_2	PTSA•H_2O	100	78
15[c]	$Pd(TFA)_2$ (15)	bipy (15)	CH_2Cl_2	PTSA•H_2O	100	50
16[c]	$Pd(TFA)_2$ (15)	bipy (10)	CH_2Cl_2	PTSA•H_2O	100	27
17[c]	$Pd(TFA)_2$ (15)	bipy (25)	CH_2Cl_2	PivOH	100	n.r.
18[c]	$Pd(TFA)_2$ (15)	bipy (25)	CH_2Cl_2	AcOH	100	n.r.
19[c]	$Pd(TFA)_2$ (15)	bipy (25)	CH_2Cl_2	PhCOOH	100	n.r.
20[c]	$Pd(TFA)_2$ (15)	bipy (25)	$CHCl_3$	PTSA•H_2O	100	46
21[c]	$Pd(TFA)_2$ (15)	bipy (25)	1,2-DCE	PTSA•H_2O	100	50
22[c]	$Pd(TFA)_2$ (15)	bipy (25)	toluene	PTSA•H_2O	100	n.r.
23[c]	$Pd(TFA)_2$ (15)	bipy (25)	PhCl	PTSA•H_2O	100	n.r.

[a]Reaction conditions: Propargylamine **1a** (0.15 mmol), 2-(2-oxo-2-phenylethyl)malononitrile (**2a**) (0.1 mmol), Pd catalyst (15 mol%), ligand (30 mol%), and additives (2.0 equiv.) in dichloromethane (2 mL) under nitrogen atmosphere at 100 °C for 12 h. [b]Isolated yields. n.r. = no reaction. [c]For 6 h. [d]For 4 h.

5. General Procedures for Pd-catalyzed Annulation Reaction

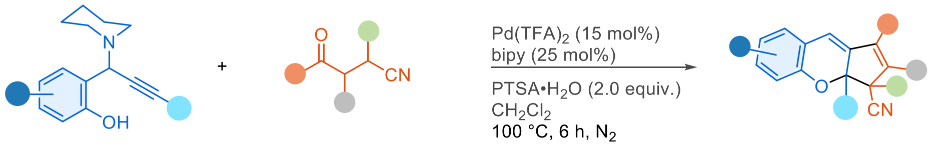

1 **2** or **4** or **6** **3** or **5** or **7**

Pd(TFA)$_2$ (15 mol%) and bipy (25 mol%) were loaded into a Schlenk tube with a Teflon-coated magnetic stir bar. Propargylamine **1** (0.3 mmol), 2-(2-oxo-2-arylylethyl)malononitriles **2** or ethyl 2-cyano-4-oxo-4-phenylbutanoate (**4**) or ethyl 2-cyano-4-oxo-4-phenylbutanoate (**6**) (1.0 equiv.) and *p*-toluenesulfonic acid monohydrate (PTSA•H_2O) (2.0 equiv.) were loaded to Schlenk tube, and the system was further evacuated and flushed with nitrogen for three cycles. Dichloromethane (CH_2Cl_2) (2.0 mL) was added with stirring at room temperature for several minutes. The tube was then placed into a pre-heated oil bath at 100 ^{o}C and stirred for 6 hours. The reaction tube was allowed to cool to room temperature. CH_2Cl_2 and brine were added for extraction. The organic layer was collected and purified by flash column chromatography with ethyl acetate and petroleum ether as the elution solvent to give desired product **3** or **5** or **7**.

6. General Procedures for Gram-scale Synthesis of Compound 3aa

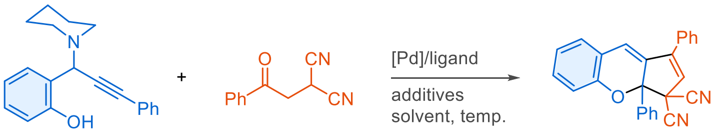

1a **2a** **3aa**

Pd(TFA)$_2$ (15 mol%) and bipy (25 mol%) were loaded into a Schlenk tube with a Teflon-coated magnetic stir bar. Propargylamine **1** (7.5 mmol), 2-(2-oxo-2-arylylethyl)malononitriles **2** (5.0 mmol) and *p*-toluenesulfonic acid monohydrate (PTSA•H_2O) (2.0 equiv.) were loaded to Schlenk tube, and the system was further evacuated and flushed with nitrogen for three cycles. Dichloromethane (CH_2Cl_2) (20 mL) was added with stirring at room temperature for several minutes. The tube was then placed into a pre-heated oil bath at 100 ^{o}C and stirred for 6 hours. The reaction tube was allowed to cool to room temperature. CH_2Cl_2 and brine were added for extraction. The organic layer was collected and purified by flash column chromatography with ethyl acetate and petroleum ether as the elution solvent to give desired product **3aa** in 65% (1.21 g).

7. General Procedures for Synthesis of Product 8

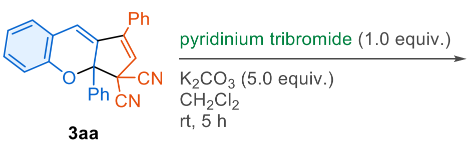

According to the reported literature,[4] compound **3aa** (0.2 mmol, 1.0 equiv.) in CH_2Cl_2, pyridinium tribromide (1.0 equiv.) and K_2CO_3 (5.0 equiv.) were added in a reaction flask under air atmosphere. Then the solution was mixed at room temperature for 5 h. After the completion of reaction (monitored by TLC), the crude products were extracted with CH_2Cl_2 (3 × 20 mL) and washed with brine. The organic layers were combined, dried over Na_2SO_4, filtered, and then evaporated under vacuum. The residue was purified using flash column chromatography with a silica gel (200-300 mesh), using ethyl acetate and petroleum ether (1:8, v/v) as the elution solvent to give desired product **8** in 85% yield (90 mg).

8. General Procedures for Synthesis of Product 9

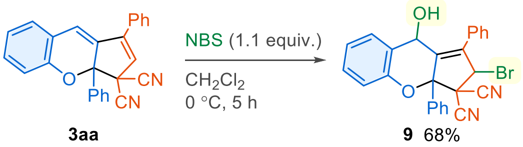

An oven-dried reaction flask was equipped with a Teflon-coated magnetic stir bar, was charged compound **3aa** (0.2 mmol, 1.0 equiv.). Anhydrous CH_2Cl_2 (3 mL) was added, and the solution was cooled to 0 °C in ice-water bath. *N*-Bromosuccinimide (0.22 mmol, 1.1 equiv.) was added to the reaction mixture under air atmosphere. Upon completion of addition, the reaction mixture was kept at 0 °C and stirred for 5 h. After the completion of reaction (monitored by TLC), the solvent was evaporated under reduced pressure. The residue was purified using flash column chromatography with a silica gel (200-300 mesh), using ethyl acetate and petroleum ether (1:10, v/v) as the elution solvent to give desired product **9** in 68% yield (64 mg).

9. General Procedures for Synthesis of Product 13

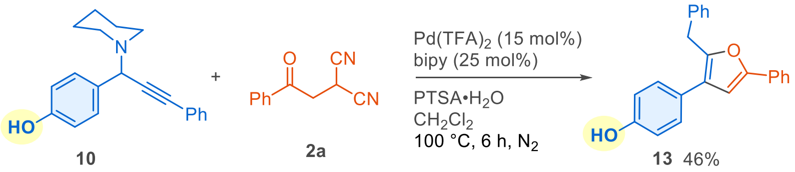

An oven-dried pressure tube was equipped with a Teflon-coated magnetic stir bar, was charged 4-(3-phenyl-1-(piperidin-1-yl)prop-2-yn-1-yl)phenol (**10**) (0.3 mmol, 1.5 equiv.), 2-(2-oxo-2-phenylethyl)malononitriles (**2a**) (1.0 equiv.), $Pd(TFA)_2$ (15 mol%), bipy (25 mol%), PTSA•H_2O (2.0 equiv.) and CH_2Cl_2 (2 mL). The tube was sealed under nitrogen and heated to 100 ^{o}C with stirring for 6 hours. After completion of the reaction (monitored by TLC analysis), the reaction mixture was admixed with ethyl acetate and the organic layer was washed with saturated sodium bicarbonate solution. The organic layer was dried over anhydrous Na_2SO_4, and the solvent was evaporated under reduced pressure, which was purified by silica gel column chromatography eluting with ethyl acetate and petroleum ether (1:8, v/v) to give the product **13** in 46% yield (30 mg).

10. X-ray Crystallographic Data of Product 3aa

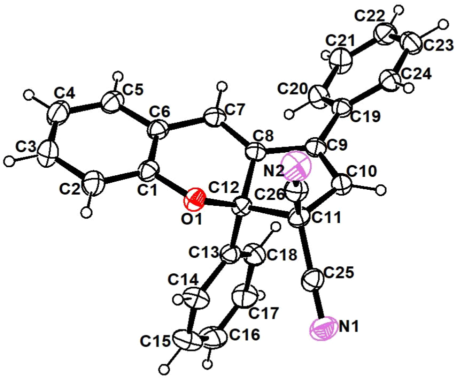

The purified compound **3aa** is dissolved in a mixed solvent of ethyl acetate and petroleum ether, and placed in a dark cabinet to evaporate slowly. After several days, a colourless bulk crystal was obtained. The X-ray crystal-structure determinations were obtained on a Bruker Smart CCDC APEX-2 diffractometer (graphite-monochromated Mo *Kα* radiation, *λ*=0.71073 nm) at 300 K.

Figure S1. ORTEP drawing of product **3aa** (CCDC 2431986) (The ellipsoid contour 30% probability levels)

Table S3. Crystal data and structure refinement for product **3aa**

Identification code	220606b_0m_a	
Empirical formula	C26H16N2O	
Formula weight	372.41	
Temperature	300 K	
Radiation	MoKα (λ = 0.71073 Å)	
Crystal system	triclinic	
Space group	P - 1	
Unit cell dimensions	a = 8.8554(12) Å	α= 109.005(12)°
	b = 10.0293(14) Å	β= 101.864(2)°
	c = 12.3183(17) Å	γ= 91.654(2)°
Volume	1006.9(2) $Å^3$	
Z	2	
Density (calculated)	1.228 g/cm^3	
μ	0.076 mm^{-1}	
F(000)	388.0	
Crystal size	0.21 × 0.2 × 0.19 mm^3	
2Θ range for data collection	3.592 to 55.08°	
Index ranges	-11≤h≤11, -13≤k≤13, -15≤l≤15	
Reflections collected	11829	
Independent reflections	4559[R_{int} = 0.0324, R_{sigma} = 0.0434]	
Data/restraints/parameters	4559 / 0 / 263	
Goodness-of-fit on F^2	1.068	
Final R indices [I>2σ (I)]	R_1 = 0.0442, wR_2 = 0.1084	
Final R indices (all data)	R_1 = 0.0650, wR_2 = 0.1182	
Largest diff. peak/hole	0.20 and -0.14 e.$Å^{-3}$	

11. X-ray Crystallographic Data of Product 9

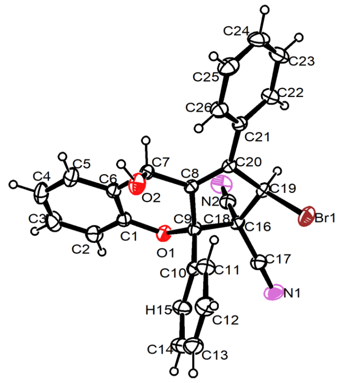

The purified compound **9** is dissolved in a mixed solvent of ethyl acetate and petroleum ether, and placed in a dark cabinet to slowly evaporate. After several days, a colourless bulk crystal was obtained. The X-ray crystal-structure determinations were obtained on a Bruker Smart CCDC APEX-2 diffractometer (graphite- monochromated Mo *Kα* radiation, *λ*=0.71073 nm) at 298 K.

Figure S2. ORTEP drawing of product **9** (CCDC 2431993) (The ellipsoid contour 30% probability levels)

Table S4. Crystal data and structure refinement for product **9**

Identification code	20250224m	
Empirical formula	C26H17BrN2O2	
Formula weight	469.32	
Temperature	298 K	
Radiation	MoKα (λ = 0.71073 Å)	
Crystal system	triclinic	
Space group	P -1	
Unit cell dimensions	a = 8.102(2) Å	α= 76.986(9)°
	b = 11.0473(19) Å	β= 86.512(10)°
	c = 12.108(3) Å	γ= 84.039(10)°
Volume	1049.5(4) $Å^3$	
Z	2	
Density (calculated)	1.485 g/cm^3	
μ	1.985 mm^{-1}	
F(000)	476.0	
Crystal size	0.2507 × 0.1661 × 0.0609 mm^3	
2Θ range for data collection	5.674 to 59.184°	
Index ranges	-11≤h≤11, -15≤k≤15, -16≤l≤16	
Reflections collected	27006	
Independent reflections	5823[R_{int} = 0.0715, R_{sigma} = 0.0587]	
Data/restraints/parameters	5823 / 0 / 281	
Goodness-of-fit on F^2	1.011	
Final R indices [I>2σ (I)]	R_1 = 0.0400, wR_2 = 0.0871	
Final R indices (all data)	R_1 = 0.0783, wR_2 = 0.0999	
Largest diff. peak/hole	0.29 and -0.46 e.$Å^{-3}$	

12. X-ray Crystallographic Data of Product 13

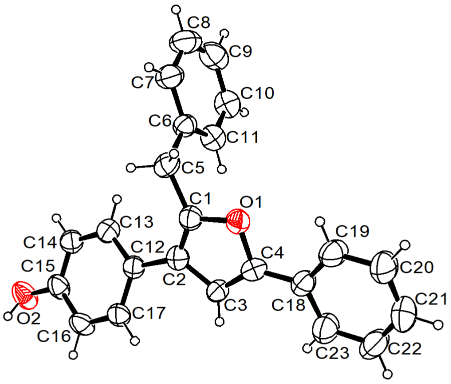

The purified compound **13** is dissolved in a mixed solvent of ethyl acetate and petroleum ether, and placed in a dark cabinet to evaporate slowly. After several days, a colourless bulk crystal was obtained. The X-ray crystal-structure determinations were obtained on a Bruker Smart CCDC APEX-2 diffractometer (graphite-monochromated Mo *Kα* radiation, *λ*=0.71073 nm) at 298 K.

Figure S3. ORTEP drawing of product **13** (CCDC 2431994) (The ellipsoid contour 30% probability levels)

Table S5. Crystal data and structure refinement for product **13**

Identification code	241114a	
Empirical formula	C23H18O2	
Formula weight	326.37	
Temperature	298 K	
Radiation	MoKα (λ = 0.71073 Å)	
Crystal system	hexagonal	
Space group	P 6_3/m	
Unit cell dimensions	a = 24.198(10) Å	α= 90(12)°
	b = 24.198(10) Å	β= 90(2)°
	c = 6.008(4) Å	γ= 120(2)°
Volume	3047(3) $Å^3$	
Z	6	
Density (calculated)	1.067 g/cm^3	
μ	0.067 mm^{-1}	
F(000)	1032.0	
Crystal size	0.13 × 0.12 × 0.10 mm^3	
2Θ range for data collection	1.944 to 54.506°	
Index ranges	-30≤h≤15, 0≤k≤31, 0≤l≤7	
Reflections collected	4530	
Independent reflections	2493[R_{int} = 0.1011, R_{sigma} = 0.0934]	
Data/restraints/parameters	2493 / 10 / 224	
Goodness-of-fit on F^2	0.917	
Final R indices [I>2σ (I)]	R_1 = 0.0755, wR_2 = 0.2240	
Final R indices (all data)	R_1 = 0.1517, wR_2 = 0.2745	
Largest diff. peak/hole	0.19 and -0.22 e.$Å^{-3}$	

13. Characterization Data for All Products

1,3a-Diphenylcyclopenta[*b*]chromene-3,3(3a*H*)-dicarbonitrile (product 3aa)

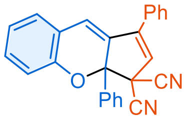

This compound was purified by column chromatography (ethyl acetate/petroleum ether = 1:10, R_f = 0.5) to afford a yellow solid in 78% yield (58 mg); m.p. 170 – 171 °C. **^{1}H NMR** (500 MHz, $CDCl_3$) δ 7.72 – 7.70 (m, 2H), 7.58 (dd, *J* = 5.0 Hz, 2.1 Hz, 3H), 7.56 – 7.54 (m, 2H), 7.34 – 7.32 (m, 3H), 7.26 – 7.22 (m, 2H), 7.12 (dd, *J* = 7.5, 1.5 Hz, 1H), 6.98 – 6.95 (m, 2H), 6.14 (s, 1H); **^{13}C NMR** (101 MHz, DMSO-*d*$_6$) δ 151.5, 149.3, 136.6, 135.4, 131.2 (2C), 130.8, 130.6, 129.9, 129.3, 129.2, 128.4, 127.0, 123.9, 122.0 (2C), 121.6, 117.9, 112.9 (2C), 87.3, 51.2; **HRMS** (ESI-TOF) *m/z*: $[M+H]^+$ Calcd for: $C_{26}H_{17}N_2O$ 373.1335; found 373.1330.

7-Methyl-1,3a-diphenylcyclopenta[*b*]chromene-3,3(3a*H*)-dicarbonitrile (product 3ba)

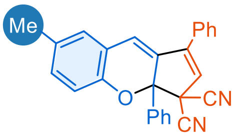

This compound was purified by column chromatography (ethyl acetate/petroleum ether = 1:10, R_f = 0.6) to afford a yellow solid in 79% yield (61 mg); m.p. 252 – 253 °C. **^{1}H NMR** (400 MHz, DMSO-*d*$_6$) δ 7.87 – 7.81 (m, 2H), 7.63 (d, *J* = 5.4 Hz, 3H), 7.52 – 7.47 (m, 2H), 7.38 (d, *J* = 5.3 Hz, 2H), 7.23 (d, *J* = 7.6 Hz, 2H), 7.15 (s, 1H), 7.10 (d, *J* = 8.3 Hz, 1H), 7.05 (d, *J* = 6.6 Hz, 1H), 6.91 (s, 1H), 2.18 (s, 3H); **^{13}C NMR** (101 MHz, DMSO-*d*$_6$) δ 149.5, 148.8, 136.6, 135.5, 132.9, 131.7, 131.2, 130.8, 130.5, 129.9, 129.4, 129.1, 128.4, 127.0, 122.0, 121.8, 121.7, 117.7, 112.9 (2C), 87.3, 51.1, 20.1; **HRMS** (ESI-TOF) *m/z*: $[M+H]^+$ Calcd for: $C_{27}H_{19}N_2O$ 387.1492; found 387.1494.

7-Bromo-1,3a-diphenylcyclopenta[*b*]chromene-3,3(3a*H*)-dicarbonitrile (product 3ca)

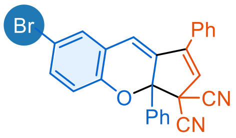

This compound was purified by column chromatography (ethyl acetate/petroleum ether = 1:10, R_f = 0.6) to afford a yellow solid in 83% yield (75 mg); m.p. 265 – 266 °C. **^{1}H NMR** (400 MHz, DMSO-*d*$_6$) δ 7.88 – 7.82 (m, 2H), 7.67 – 7.60 (m, 4H), 7.53 – 7.45 (m, 3H), 7.44 – 7.39 (m, 3H), 7.36 (d, *J* = 8.8 Hz, 1H), 7.32 (s, 1H), 6.99 (s, 1H); **^{13}C NMR** (101 MHz, DMSO-*d*$_6$) δ 150.2, 148.6, 136.1, 134.6, 133.0, 130.9, 130.8, 130.3, 130.2, 129.5, 128.8, 128.0, 126.0, 123.8, 121.7, 120.2, 115.60, 115.6, 112.3, 109.8, 86.4, 50.6; **HRMS** (ESI-TOF) *m/z*: $[M+H]^+$ Calcd for: $C_{26}H_{16}BrN_2O$ 451.0441; found 451.0439.

7-Chloro-1,3a-diphenylcyclopenta[*b*]chromene-3,3(3a*H*)-dicarbonitrile (product 3da)

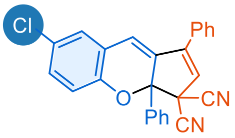

This compound was purified by column chromatography (ethyl acetate/petroleum ether = 1:10, R_f = 0.6) to afford a yellow solid in 75% yield (61 mg); m.p. 226 – 227 °C. **^{1}H NMR** (400 MHz, DMSO-d_6) δ 7.88 – 7.82 (m, 2H), 7.63 (d, J = 3.2 Hz, 3H), 7.53 – 7.47 (m, 3H), 7.46 – 7.38 (m, 4H), 7.35 (dd, J = 8.6 Hz, 2.6 Hz, 1H), 7.31 (s, 1H), 6.99 (s, 1H); **^{13}C NMR** (101 MHz, DMSO-d_6) δ 150.2, 149.0, 135.6, 131.3, 130.8, 130.6, 130.0, 129.2, 128.5, 128.4, 127.6, 127.0, 126.2, 123.7, 123.0, 120.7, 119. 8, 112.7, 112.7, 87.5, 51.1; **HRMS** (ESI-TOF) *m/z*: $[M+H]^+$ Calcd for: $C_{26}H_{16}ClN_2O$ 407.0946; found 407.0954.

7-Fluoro-1,3a-diphenylcyclopenta[*b*]chromene-3,3(3a*H*)-dicarbonitrile (product 3ea)

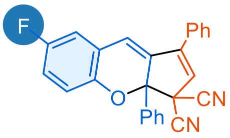

This compound was purified by column chromatography (ethyl acetate/petroleum ether = 1:10, R_f = 0.6) to afford a yellow solid in 68% yield (53 mg); m.p. 206-207 °C. **^{1}H NMR** (400 MHz, DMSO-d_6) δ 7.88 – 7.80 (m, 2H), 7.67 – 7.61 (m, 3H), 7.54 – 7.46 (m, 2H), 7.45 – 7.36 (m, 4H), 7.29 (s, 1H), 7.26 (dd, J = 8.8, 3.1 Hz, 1H), 7.16 (td, J = 8.7 Hz, 3.1 Hz, 1H), 6.98 (s, 1H); **^{13}C NMR** (101 MHz, DMSO-d_6) δ 158.8, 156.4, 148.6, 147.2, 136.3, 135.7, 130.8, 130.2 (d, J_{C-F} = 3.8 Hz), 129.5, 128.8, 128.0, 126.6, 123.7, 122.8 (d, J_{C-F} = 9.2 Hz), 122.5, 120.5, 119.0 (d, J_{C-F} = 8.5 Hz), 117.1 (d, J_{C-F} = 23.8 Hz), 114.6 (d, J_{C-F} = 24.3 Hz), 112.3 (d, J_{C-F} = 5.1 Hz), 87.0, 50.7; **^{19}F NMR** (376 MHz, DMSO-d_6) δ -119.6; **HRMS** (ESI-TOF) *m/z*: $[M+H]^+$ Calcd for: $C_{26}H_{16}FN_2O$ 391.1241; found 391.1235.

5-Fluoro-1,3a-diphenylcyclopenta[*b*]chromene-3,3(3a*H*)-dicarbonitrile (product 3fa)

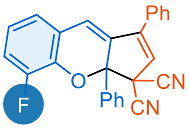

This compound was purified by column chromatography (ethyl acetate/petroleum ether = 1:10, R_f = 0.6) to afford a yellow solid in 62% yield (48 mg); m.p. 184 – 185 °C. **^{1}H NMR** (400 MHz, DMSO-d_6) δ 7.91 – 7.80 (m, 2H), 7.67 – 7.59 (m, 3H), 7.53 – 7.40 (m, 5H), 7.38 (s, 1H), 7.30 (t, J = 9.5 Hz, 1H), 7.23 (d, J = 7.6 Hz, 1H), 7.08 – 7.02 (m, 1H), 7.01 (s, 1H); **^{13}C NMR** (101 MHz, DMSO-d_6) δ 151.9, 149.5, 148.7, 138.3 (d, J_{C-F} = 11.5 Hz), 136.1, 135.5, 130.8, 130.5, 130.1, 129.5, 129.0, 128.0, 126.0, 124.4 (d, J_{C-F} = 3.2 Hz), 124.0, 123.9 (d, J_{C-F} = 7.2 Hz), 122.4, 120.7 (d, J_{C-F} = 3.3 Hz), 117.5 (d, J_{C-F} = 12.2 Hz), 112.1 (d, J_{C-F} = 9.2 Hz), 87.1, 50.5; **^{19}F NMR** (376 MHz, DMSO-d_6) δ -135.8; **HRMS** (ESI-TOF) *m/z*: $[M+H]^+$ Calcd for: $C_{26}H_{16}FN_2O$ 391.1241; found

391.1250.

5-Bromo-1,3a-diphenylcyclopenta[*b*]chromene-3,3(3a*H*)-dicarbonitrile (product 3ga)

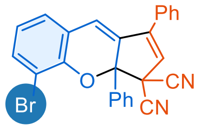

This compound was purified by column chromatography (ethyl acetate/petroleum ether = 1:10, R_f = 0.6) to afford a yellow solid in 71% yield (64 mg); m.p. 188 – 189 °C. **^{1}H NMR** (400 MHz, DMSO-*d*$_6$) δ 7.86 (d, *J* = 4.4 Hz, 2H), 7.63 (s, 3H), 7.59 (d, *J* = 7.8 Hz, 1H), 7.56 – 7.52 (m, 2H), 7.46 – 7.39 (m, 4H), 7.35 (s, 1H), 7.05 – 6.97 (m, 2H); **^{13}C NMR** (101 MHz, DMSO-*d*$_6$) δ 148.6, 147.6, 135.5 (2C), 133.8, 130.8, 130.5, 130.1, 129.5, 128.9, 128.4, 128.0, 126.2, 124.8, 123.4, 122.30, 120.9, 112.3, 112.0, 110.6, 87.8, 50.7; **HRMS** (ESI-TOF) *m/z*: [M+H]$^+$ Calcd for: $C_{26}H_{16}BrN_2O$ 451.0441; found 451.0440.

5-Chloro-1,3a-diphenylcyclopenta[*b*]chromene-3,3(3a*H*)-dicarbonitrile (product 3ha)

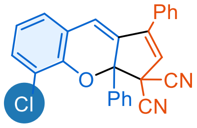

This compound was purified by column chromatography (ethyl acetate/petroleum ether = 1:10, R_f = 0.6) to afford a yellow solid in 74% yield (60 mg); m.p. 186 – 187 °C. **^{1}H NMR** (400 MHz, DMSO-*d*$_6$) δ 7.90 – 7.83 (m, 2H), 7.65 – 7.61 (m, 3H), 7.55 – 7.49 (m, 2H), 7.49 – 7.41 (m, 4H), 7.41 – 7.31 (m, 2H), 7.07 (t, *J* = 7.8 Hz, 1H), 7.01 (s, 1H); **^{13}C NMR** (101 MHz, DMSO-*d*$_6$) δ 148.6, 146.5, 135.7, 135.5, 130.8, 130.5, 130.1, 129.5, 128.9, 128.0, 127.7, 126.1, 124.3, 123.4, 122.8, 121.3, 120.8, 112.2, 112.0, 87.6, 50.6; **HRMS** (ESI-TOF) *m/z*: [M+H]$^+$ Calcd for: $C_{26}H_{16}ClN_2O$ 407.0946; found 407.0943.

5-Methoxy-1,3a-diphenylcyclopenta[*b*]chromene-3,3(3a*H*)-dicarbonitrile (product 3ia)

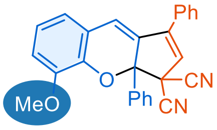

This compound was purified by column chromatography (ethyl acetate/petroleum ether = 1:8, R_f = 0.6) to afford a yellow solid in 75% yield (60 mg); m.p. 209 – 210 °C. **^{1}H NMR** (400 MHz, DMSO-*d*$_6$) δ 7.88 – 7.79 (m, 2H), 7.66 – 7.60 (m, 3H), 7.51 – 7.45 (m, 2H), 7.39 (dd, *J* = 5.2 Hz, 2.0 Hz, 3H), 7.26 (s, 1H), 7.10 – 7.03 (m, 2H), 6.98 – 6.94 (m, 2H), 3.96 (s, 3H); **^{13}C NMR** (101 MHz, DMSO-*d*$_6$) δ 148.9, 148.3, 139.9, 136.1, 135.3, 130.7, 130.4, 130.2, 129.5, 128.8, 128.0, 125.9, 123.4, 122.3, 121.8, 121.2, 120.4, 114.0, 112.4, 112.4, 87.0, 56.2, 50.7; **HRMS** (ESI-TOF) *m/z*: [M+H]$^+$ Calcd for: $C_{27}H_{19}N_2O_2$ 403.1441; found 403.1445.

5,7-Dibromo-1,3a-diphenylcyclopenta[*b*]chromene-3,3(3a*H*)-dicarbonitrile (product 3ja)

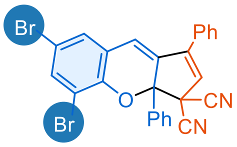

This compound was purified by column chromatography (ethyl acetate/petroleum ether = 1:10, R_f = 0.6) to afford a yellow solid in 70% yield (74 mg); m.p. 195 – 196 °C. **^{1}H NMR** (400 MHz, DMSO-d_6) δ 7.84 (d, J = 2.3 Hz, 3H), 7.69 – 7.60 (m, 4H), 7.55 – 7.47 (m, 2H), 7.44 (dd, J = 5.2, 2.0 Hz, 3H), 7.36 (s, 1H), 7.05 (s, 1H); **^{13}C NMR** (101 MHz, DMSO-d_6) δ 148.3, 147.0, 136.6, 135.2, 135.1, 130.9, 130.7, 130.6, 129.9, 129.5, 128.9, 128.0, 126.2, 124.9, 123.1, 119.9, 115.4, 112.1, 111.8, 87.9, 50.6; **HRMS** (ESI-TOF) *m/z*: $[M+H]^+$ Calcd for: $C_{26}H_{15}Br_2N_2O$ 528.9546; found 528.9545.

6-Chloro-1,3a-diphenylcyclopenta[*b*]chromene-3,3(3a*H*)-dicarbonitrile (product 3ka)

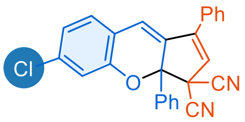

This compound was purified by column chromatography (ethyl acetate/petroleum ether = 1:10, R_f = 0.6) to afford a yellow solid in 67% yield (54 mg); m.p. 219 – 220 °C. **^{1}H NMR** (400 MHz, DMSO-d_6) δ 7.88 – 7.81 (m, 2H), 7.66 – 7.59 (m, 3H), 7.58 – 7.50 (m, 3H), 7.45 – 7.36 (m, 4H), 7.32 (s, 1H), 7.11 (dd, J = 8.2 Hz, 2.0 Hz, 1H), 6.96 (s, 1H); **^{13}C NMR** (101 MHz, DMSO-d_6) δ 151.7, 148.7, 135.7, 135.2, 134.3, 130.8, 130.3, 130.2, 130.0, 129.5, 128.8, 128.0, 126.6, 123.7, 122.0, 120.6, 120.4, 117.7, 112.3, 112.2, 87.1, 50.6; **HRMS** (ESI-TOF) *m/z*: $[M+H]^+$ Calcd for: $C_{26}H_{16}ClN_2O$ 407.0946; found 407.0945.

1-Phenyl-3a-(*m*-tolyl)cyclopenta[*b*]chromene-3,3(3a*H*)-dicarbonitrile (product 3la)

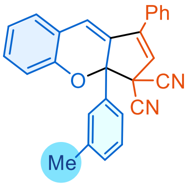

This compound was purified by column chromatography (ethyl acetate/petroleum ether = 1:10, R_f = 0.6) to afford a yellow solid in 70% yield (54 mg); m.p. 166 – 167 °C. **^{1}H NMR** (400 MHz, DMSO-d_6) δ 7.87 – 7.81 (m, 2H), 7.63 (d, J = 6.3 Hz, 3H), 7.38 – 7.34 (m, 2H), 7.33 – 7.24 (m, 5H), 7.18 (d, J = 7.2 Hz, 1H), 7.07 – 7.01(m, 1H), 6.91 (s, 1H), 2.25 (s, 3H); **^{13}C NMR** (101 MHz, DMSO-d_6) δ 151.1, 148.8, 137.9, 136.2, 134.9, 130.8, 130.7, 130.4, 129.5, 128.8, 128.6, 127.9, 127.0, 123.7, 123.6, 123.4, 121.6, 121.5, 121.1, 117.5, 112.5 (2C), 86.8, 50.8, 21.2; **HRMS** (ESI-TOF) *m/z*: $[M+H]^+$ Calcd for: $C_{27}H_{19}N_2O$ 387.1492; found 387.1500.

3a-(4-Chlorophenyl)-1-phenylcyclopenta[*b*]chromene-3,3(3a*H*)-dicarbonitrile (product 3ma)

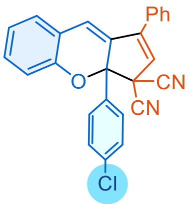

This compound was purified by column chromatography (ethyl acetate/petroleum ether = 1:10, R_f = 0.6) to afford a yellow solid in 62% yield (50 mg); m.p. 179 – 180 °C. **^{1}H NMR** (400 MHz, DMSO-d_6) δ 7.87 – 7.81 (m, 2H), 7.65 – 7.59 (m, 3H), 7.55 – 7.45 (m, 4H), 7.40 – 7.30 (m, 4H), 7.05 (t, *J* = 6.6 Hz, 1H), 6.95 (s, 1H); **^{13}C NMR** (101 MHz, DMSO-d_6) δ 151.3, 149.2, 135.6, 135.4, 134.9, 131.4, 131.2, 130.7, 129.9, 129.4, 129.3, 129.1, 128.5, 124.1, 122.0 (2C), 121.9, 118.0, 112.9, 112.7, 86.9, 51.0; **HRMS** (ESI-TOF) *m/z*: [M+H]$^+$ Calcd for: $C_{26}H_{16}ClN_2O$ 407.0946; found 407.0953.

3a-(4-Bromophenyl)-1-phenylcyclopenta[*b*]chromene-3,3(3a*H*)-dicarbonitrile (product 3na)

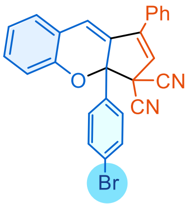

This compound was purified by column chromatography (ethyl acetate/petroleum ether = 1:10, R_f = 0.6) to afford a yellow solid in 76% yield (68 mg); m.p. 167 – 168 °C. **^{1}H NMR** (400 MHz, DMSO-d_6) δ 7.87 – 7.82 (m, 2H), 7.67 – 7.58 (m, 5H), 7.46 (d, *J* = 8.7 Hz, 2H), 7.41 – 7.31 (m, 4H), 7.08 – 7.03 (m, 1H), 6.94 (s, 1H); **^{13}C NMR** (101 MHz, DMSO-d_6) δ 151.3, 149.2, 136.0, 134.9, 132.3, 131.4, 131.2, 130.7, 129.9, 129.4, 129.3, 128.5, 124.2, 124.1, 122.0 (2C), 121.9, 117.9, 112.9, 112.7, 86.9, 51.0; **HRMS** (ESI-TOF) *m/z*: [M+H]$^+$ Calcd for: $C_{26}H_{16}BrN_2O$ 451.0441; found 451.0447.

3a-(4-Fluorophenyl)-1-phenylcyclopenta[*b*]chromene-3,3(3a*H*)-dicarbonitrile (product 3oa)

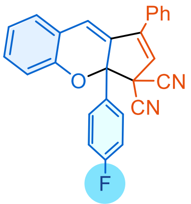

This compound was purified by column chromatography (ethyl acetate/petroleum ether = 1:10, R_f = 0.6) to afford a yellow solid in 80% yield (62 mg); m.p. 171 – 172 °C. **^{1}H NMR** (400 MHz, $CDCl_3$) δ 7.75 – 7.67 (m, 2H), 7.62 – 7.56 (m, 3H), 7.56 – 7.49 (m, 2H), 7.32 – 7.26 (m, 1H), 7.21 (d, *J* = 7.1 Hz, 1H), 7.14 (dd, *J* = 7.6 Hz, 1.6 Hz, 1H), 7.06 – 6.93 (m, 4H), 6.15 (s, 1H); **^{13}C NMR**

(101 MHz, DMSO-d_6) δ 164.0, 161.6, 150.9, 148.7, 134.7, 132.3 (d, $J_{C\text{-}F}$ = 3.0 Hz), 130.9, 130.6, 130.3, 129.5, 129.1 (d, $J_{C\text{-}F}$ = 8.7 Hz), 128.9, 128.0, 123.6, 121.6, 121.4 (d, $J_{C\text{-}F}$ = 4.7 Hz), 117.5, 115.8 (d, $J_{C\text{-}F}$ = 21.9 Hz), 112.5, 112.3, 86.5, 50.7; **^{19}F NMR** (376 MHz, DMSO-d_6) δ -110.9; **HRMS** (ESI-TOF) *m/z*: $[M+H]^+$ Calcd for $C_{26}H_{16}FN_2O$ 391.1241, found 391.1244.

1-Phenyl-3a-(*p*-tolyl)cyclopenta[*b*]chromene-3,3(3a*H*)-dicarbonitrile (product 3pa)

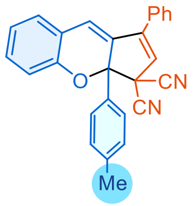

This compound was purified by column chromatography (ethyl acetate/petroleum ether = 1:10, R_f = 0.6) to afford a yellow solid in 79% yield (61 mg); m.p. 203 – 204 °C. **^{1}H NMR** (400 MHz, DMSO-d_6) δ 7.86 – 7.81 (m, 2H), 7.66 – 7.60 (m, 3H), 7.40 – 7.29 (m, 5H), 7.27 (s, 1H), 7.18 (d, *J* = 8.0 Hz, 2H), 7.06 – 7.00 (m, 1H), 6.92 (s, 1H), 2.23 (s, 3H); **^{13}C NMR** (101 MHz, DMSO-d_6) δ 151.5, 149.2, 140.2, 135.5, 133.6, 131.2, 131.1, 130.9, 129.9, 129.7, 129.7, 128.4, 127.0, 123.8, 122.1, 122.0, 121.5, 117.9, 113.0, 113.0, 87.3, 51.2, 21.2; **HRMS** (ESI-TOF) *m/z*: $[M+H]^+$ Calcd for $C_{27}H_{19}N_2O$ 387.1492; found 387.1491.

3a-([1,1'-Biphenyl]-4-yl)-1-phenylcyclopenta[*b*]chromene-3,3(3a*H*)-dicarbonitrile (product 3qa)

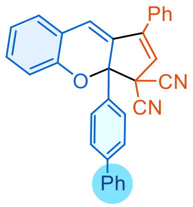

This compound was purified by column chromatography (ethyl acetate/petroleum ether = 1:10, R_f = 0.6) to afford a yellow solid in 72% yield (65 mg); m.p. 220 – 221 °C.**^{1}H NMR** (400 MHz, DMSO-d_6) δ 7.91 – 7.85 (m, 2H), 7.70 (d, *J* = 8.3 Hz, 2H), 7.67 – 7.60 (m, 6H), 7.58 (s, 1H), 7.46 – 7.32 (m, 7H), 7.05 (t, *J* = 7.4 Hz, 1H), 6.97 (s, 1H); **^{13}C NMR** (101 MHz, DMSO-d_6) δ 151.1, 148.8, 141.6, 138.8, 135.2, 134.8, 130.9, 130.8, 130.4, 129.5, 129.0, 128.9, 128.0 (2C), 127.3, 126.9, 126.7, 123.5, 121.6, 121.5, 121.3, 117.5, 112.6, 112.4, 86.8, 50.7; **HRMS** (ESI-TOF) *m/z*: $[M+H]^+$ Calcd for: $C_{32}H_{21}N_2O$ 449.1648; found 449.1652.

1-Phenyl-3a-(4'-propyl-[1,1'-biphenyl]-4-yl)cyclopenta[*b*]chromene-3,3(3a*H*)-dicarbonitrile (product 3ra)

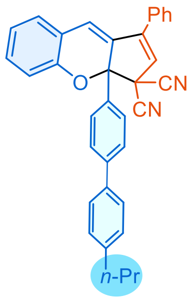

This compound was purified by column chromatography (ethyl acetate/petroleum ether = 1:10, R_f = 0.6) to afford a yellow solid in 62% yield (61 mg); m.p. 212 – 213 °C. **^{1}H NMR** (400 MHz, DMSO-d_6) δ 7.93 – 7.81 (m, 2H), 7.69 – 7.62 (m, 5H), 7.55 (dd, *J* = 11.8, 8.2 Hz, 4H), 7.38 (d, *J* = 9.8 Hz, 2H), 7.36 – 7.28 (m, 2H), 7.24 (d, *J* = 8.0 Hz, 2H), 7.05 (t, *J* = 7.4 Hz, 1H), 6.96 (s, 1H), 2.56 (t, *J* = 7.5 Hz, 2H), 1.58 (h, *J* = 7.3 Hz, 2H), 0.88 (t, *J* = 7.3 Hz, 3H); **^{13}C NMR** (101 MHz, DMSO-d_6) δ 151.1, 148.8, 142.1, 141.6, 136.2, 134.8, 134.8, 130.8, 130.8, 130.4, 129.5, 129.0, 128.9, 128.0, 127.2, 126.6, 123.5, 121.6, 121.5, 121.2, 117.5, 112.6, 112.5, 86.9, 50.7, 36.8, 23.9, 13.7; **HRMS** (ESI-TOF) *m/z*: $[M+H]^+$ Calcd for: $C_{35}H_{27}N_2O$ 491.2118; found 491.2109.

5-Methoxy-1-phenyl-3a-(*p*-tolyl)cyclopenta[*b*]chromene-3,3(3a*H*)-dicarbonitrile (product 3sa)

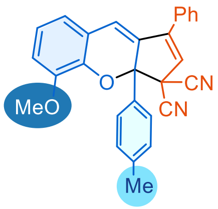

This compound was purified by column chromatography (ethyl acetate/petroleum ether = 1:10, R_f = 0.6) to afford a yellow solid in 69% yield (57 mg); m.p. 246 – 247 °C. **^{1}H NMR** (400 MHz, DMSO-d_6) δ 7.86 – 7.78 (m, 2H), 7.67 – 7.58 (m, 3H), 7.36 (d, *J* = 8.3 Hz, 2H), 7.23 (s, 1H), 7.18 (d, *J* = 8.1 Hz, 2H), 7.06 (dd, *J* = 7.9 Hz, 1.8 Hz, 1H), 7.01 – 6.91 (m, 3H), 3.94 (s, 3H), 2.23 (s, 3H); **^{13}C NMR** (101 MHz, DMSO-d_6) δ 148.8, 148.3, 140.0, 139.8, 135.5, 133.1, 130.7, 130.4, 129.5, 129.3, 127.9, 125.8, 123.3, 122.3, 121.8, 121.0, 120.3, 113.9, 112.5, 112.5, 87.0, 56.2, 50.7, 20.7; **HRMS** (ESI-TOF) *m/z*: $[M+H]^+$ Calcd for: $C_{28}H_{21}N_2O_2$ 417.1598; found 417.1605.

7-Bromo-3a-(4-ethylphenyl)-1-phenylcyclopenta[*b*]chromene-3,3(3a*H*)-dicarbonitrile (product 3ta)

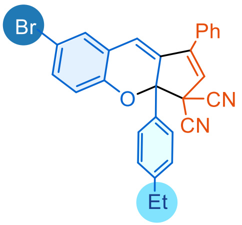

This compound was purified by column chromatography (ethyl acetate/petroleum ether = 1:10, R_f = 0.6) to afford a yellow solid in 65% yield (62 mg); m.p. 188 – 189 °C. **^{1}H NMR** (400 MHz, DMSO-d_6) δ 7.86 – 7.80 (m, 2H), 7.63 (d, *J* = 1.9 Hz, 3H), 7.59 (d, *J* = 2.4 Hz, 1H), 7.45 (dd, *J* = 8.6, 2.4 Hz, 1H), 7.41 (s, 1H), 7.39 (s, 1H), 7.33 (d, *J* = 8.7 Hz, 1H), 7.29 (s, 1H), 7.23 (s, 1H), 7.21 (s, 1H), 6.97 (s, 1H), 2.58 – 2.51 (q, *J* = 7.6 Hz, 2H), 1.09 (t, *J* = 7.6 Hz, 3H); **^{13}C NMR** (101 MHz, DMSO-d_6) *δ* 150.3, 148.5, 146.0, 136.2, 132.9, 130.9, 130.8, 130.2, 129.5, 128.1, 127.9, 126.8, 126.6, 123.8, 122.5, 120.0, 119.7, 114.9, 112.4, 112.3, 87.0, 50.6, 27.7, 15.0; **HRMS** (ESI-TOF) *m/z*: [M+H]$^+$ Calcd for: $C_{28}H_{20}BrN_2O$ 479.0754; found 479.0751.

3a-(4-Chlorophenyl)-7-methoxy-1-phenylcyclopenta[*b*]chromene-3,3(3a*H*)-dicarbonitrile (product 3ua)

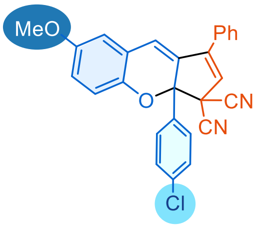

This compound was purified by column chromatography (ethyl acetate/petroleum ether = 1:10, R_f = 0.6) to afford a yellow solid in 85% yield (74 mg); m.p. 205 – 206 °C. **^{1}H NMR** (400 MHz, DMSO-d_6) δ 7.86 – 7.81 (m, 2H), 7.65 – 7.59 (m, 3H), 7.49 (q, *J* = 8.9 Hz, 4H), 7.28 (d, *J* = 8.5 Hz, 2H), 7.00 (d, *J* = 3.0 Hz, 1H), 6.95 (s, 1H), 6.89 (dd, *J* = 8.9 Hz, 3.1 Hz, 1H), 3.67 (s, 3H); **^{13}C NMR** (101 MHz, DMSO-d_6) *δ* 155.0, 148.6, 144.7, 135.2, 135.1, 134.9, 130.8, 130.3, 129.5, 128.8, 128.7, 128.00 122.1, 121.8, 121.4, 118.3, 116.7, 112.9, 112.5, 112.2, 86.9, 55.5, 50.1; **HRMS** (ESI-TOF) *m/z*: [M+H]$^+$ Calcd for: $C_{27}H_{18}ClN_2O_2$ 437.1051; found 437.1055.

3a-(4-Chlorophenyl)-7-methyl-1-phenylcyclopenta[*b*]chromene-3,3(3a*H*)-dicarbonitrile (product 3va)

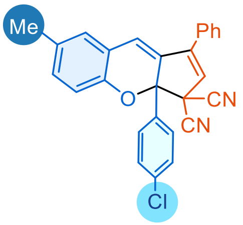

This compound was purified by column chromatography (ethyl acetate/petroleum ether = 1:10, R_f = 0.6) to afford a yellow solid in 77% yield (65 mg); m.p. 179 – 180 °C. **^{1}H NMR** (400 MHz, DMSO-d_6) δ 7.87 – 7.80 (m, 2H), 7.66 – 7.59 (m, 3H), 7.53 – 7.44 (m, 4H), 7.27 – 7.21 (m, 2H), 7.17 (s, 1H), 7.16 – 7.02 (m, 1H), 6.93 (s, 1H), 2.19 (s, 3H); **^{13}C NMR** (101 MHz, DMSO-d_6) δ 148.8, 148.7, 135.2, 134.9, 134.6, 132.6, 131.4, 130.8, 130.3, 129.5, 129.1, 128.8, 128.6, 128.0, 121.6, 121.5, 121.2, 117.3, 112.5, 112.3, 86.4, 50.5, 20.2; **HRMS** (ESI-TOF) *m/z*: [M+H]$^+$ Calcd for: $C_{27}H_{18}ClN_2O$ 421.1102; found 421.1108.

7-Chloro-3a-(4-methoxyphenyl)-1-phenylcyclopenta[*b*]chromene-3,3(3a*H*)-dicarbonitrile (product 3wa)

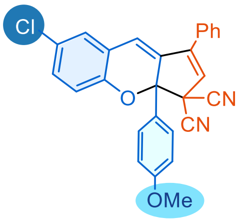

This compound was purified by column chromatography (ethyl acetate/petroleum ether = 1:10, R_f = 0.6) to afford a yellow solid in 75% yield (65 mg); m.p. 234 – 235 °C. **^{1}H NMR** (400 MHz, DMSO-d_6) δ 7.83 (dd, J = 6.7, 2.9 Hz, 2H), 7.67 – 7.59 (m, 3H), 7.48 (d, J = 2.4 Hz, 1H), 7.45 – 7.37 (m, 2H), 7.38 – 7.31 (m, 2H), 7.29 (s, 1H), 6.97 (s, 1H), 6.94 (d, J = 8.6 Hz, 2H), 2.50 (s, 3H); **^{13}C NMR** (101 MHz, DMSO-d_6) δ 160.4, 149.8, 148.5, 136.4, 130.8, 130.2, 130.1, 129.5, 128.2, 128.0, 127.9, 127.2, 127.0, 123.4, 122.6, 120.1, 119.3, 114.0, 112.4, 112.3, 87.0, 55.2, 50.7; **HRMS** (ESI-TOF) *m/z*: [M+H]$^+$ Calcd for: $C_{27}H_{18}ClN_2O_2$ 437.1051; found 437.1053.

1-Phenyl-3a-(thiophen-3-yl)cyclopenta[*b*]chromene-3,3(3a*H*)-dicarbonitrile (product 3xa)

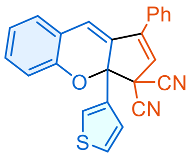

This compound was purified by column chromatography (ethyl acetate/petroleum ether = 1:10, R_f = 0.6) to afford a yellow solid in 68% yield (51 mg); m.p. 199-200 °C. **^{1}H NMR** (400 MHz, DMSO-d_6) δ 7.80 (d, J = 5.5 Hz, 2H), 7.64 (s, 1H), 7.61 (d, J = 5.8 Hz, 3H), 7.56 – 7.52 (m, 1H), 7.37 (d, J = 7.5 Hz, 1H), 7.33 (s, 2H), 7.20 (s, 1H), 7.13 (d, J = 5.2 Hz, 1H), 7.08 – 7.03 (m, 1H), 6.93 (s, 1H); **^{13}C NMR** (101 MHz, DMSO-d_6) δ 151.2, 148.3, 137.2, 134.8, 130.8, 130.6, 130.5, 129.4, 128.9, 128.0, 127.7, 126.3, 125.6, 123.4, 121.6, 121.1, 120.7, 117.2, 112.5, 112.4, 85.9, 50.3; **HRMS** (ESI-TOF) *m/z*: [M+H]$^+$ Calcd for: $C_{24}H_{15}N_2OS$ 379.0900; found 379.0908.

1-Phenyl-3a-(thiophen-2-yl)cyclopenta[*b*]chromene-3,3(3a*H*)-dicarbonitrile (product 3ya)

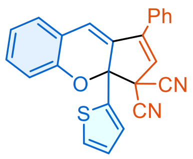

This compound was purified by column chromatography (ethyl acetate/petroleum ether = 1:10, R_f = 0.6) to afford a yellow solid in 62% yield (47 mg); m.p. 184 – 185 °C. **^{1}H NMR** (400 MHz, DMSO-d_6) δ 7.78 (d, J = 4.8 Hz, 2H), 7.62 (m, 3H), 7.58 (d, J = 5.0 Hz, 1H), 7.40 (d, J = 7.7 Hz, 1H), 7.35 (s, 2H), 7.26 (d, J = 3.6 Hz, 1H), 7.21 (s, 1H), 7.11 – 7.05 (m, 1H), 7.01 (t, J = 4.5 Hz, 1H), 6.97 (s, 1H); **^{13}C NMR** (101 MHz, DMSO-d_6) δ 151.1, 148.2, 138.7, 134.7, 131.0, 130.7, 130.5, 129.4, 129.0, 128.2, 128.1, 127.9, 127.2, 123.7, 121.5, 120.7, 117.3, 112.3, 112.2, 86.4, 51.0; **HRMS** (ESI-TOF) *m/z*: $[M+H]^+$ Calcd for: $C_{24}H_{15}N_2OS$ 379.0900; found 379.0902.

1-(4-Fluorophenyl)-3a-phenylcyclopenta[*b*]chromene-3,3(3a*H*)-dicarbonitrile (product 3ab)

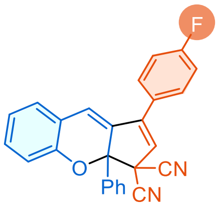

This compound was purified by column chromatography (ethyl acetate/petroleum ether = 1:10, R_f = 0.6) to afford a yellow solid in 69% yield (54 mg); m.p. 210 – 211 °C. **^{1}H NMR** (400 MHz, DMSO-d_6) δ 7.92 (dd, J = 8.7, 5.5 Hz, 2H), 7.53 – 7.44 (m, 4H), 7.43 – 7.35 (m, 4H), 7.35 (s, 1H), 7.32 (dd, J = 7.0 Hz, 1.5 Hz, 1H), 7.29 (s, 1H), 7.04 (td, J = 7.3 Hz, 1.5 Hz, 1H), 6.92 (s, 1H); **^{13}C NMR** (101 MHz, DMSO-d_6) δ 164.7, 162.2, 151.0, 147.7, 136.0, 134.8, 130.8, 130.4 (d, J_{C-F} = 8.6 Hz), 130.1, 128.9, 128.7, 126.8 (d, J_{C-F} = 3.2 Hz), 126.6, 123.5, 121.5 (2C), 121.3, 117.5, 116.5 (d, J_{C-F} = 21.6 Hz), 112.4 (d, J_{C-F} = 5.4 Hz), 86.8, 50.7; **^{19}F NMR** (376 MHz, $CDCl_3$) δ -112.3; **HRMS** (ESI-TOF) *m/z*: $[M+H]^+$ Calcd for: $C_{26}H_{16}FN_2O$ 391.1241; found 391.1248.

1-([1,1'-Biphenyl]-4-yl)-3a-phenylcyclopenta[*b*]chromene-3,3(3a*H*)-dicarbonitrile (product 3ac)

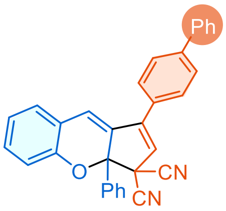

This compound was purified by column chromatography (ethyl acetate/petroleum ether = 1:10, R_f = 0.6) to afford a yellow solid in 70% yield (63 mg); m.p. 242 – 243 °C. **^{1}H NMR** (400 MHz, DMSO-d_6) δ 8.00 – 7.90 (m, 4H), 7.78 (s, 2H), 7.57 – 7.50 (m, 4H), 7.47 – 7.33 (m, 8H), 7.09 –

7.04 (m, 1H), 6.99 (s, 1H); **^{13}C NMR** (101 MHz, DMSO-d_6) δ 151.1, 148.3, 142.3, 139.1, 136.1, 134.9, 130.8, 130.1, 129.3, 129.2, 128.7, 128.7, 128.6, 128.2, 127.6, 126.8, 126.6, 123.5, 121.5, 121.4, 121.3, 117.5, 112.5, 112.5, 86.9, 50.8; **HRMS** (ESI-TOF) *m/z*: [M+H]$^+$ Calcd for: $C_{32}H_{21}N_2O$ 449.1648; found 449.1650.

3a-Phenyl-1-(*p*-tolyl)cyclopenta[*b*]chromene-3,3(3a*H*)-dicarbonitrile (product 3ad)

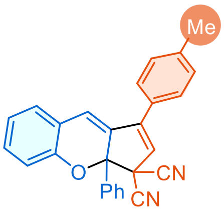

This compound was purified by column chromatography (ethyl acetate/petroleum ether = 1:10, R_f = 0.6) to afford a yellow solid in 76% yield (59 mg); m.p. 205 – 206 °C. **^{1}H NMR** (400 MHz, DMSO-d_6) δ 7.75 (d, J = 8.0 Hz, 2H), 7.54 – 7.47 (m, 2H), 7.44 (d, J = 7.9 Hz, 2H), 7.41 – 7.33 (m, 5H), 7.32 (d, J = 7.2 Hz, 1H), 7.28 (s, 1H), 7.03 (d, J = 7.4 Hz, 1H), 6.87 (s, 1H), 2.42 (s, 3H); **^{13}C NMR** (101 MHz, DMSO-d_6) δ 151.1, 148.7, 140.6, 136.2, 135.1, 130.7, 130.1, 130.0, 128.8, 128.7, 127.9, 127.5, 126.6, 123.4, 121.5, 121.1, 120.7, 117.5, 112.6, 112.5, 86.8, 50.7, 21.0; **HRMS** (ESI-TOF) *m/z*: [M+H]$^+$ Calcd for: $C_{27}H_{19}N_2O$ 387.1492; found 387.1487.

1-(3-Bromophenyl)-3a-phenylcyclopenta[*b*]chromene-3,3(3a*H*)-dicarbonitrile (product 3ae)

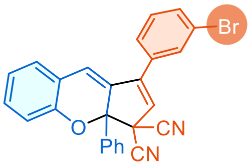

This compound was purified by column chromatography (ethyl acetate/petroleum ether = 1:10, R_f = 0.6) to afford a yellow solid in 83% yield (75 mg); m.p. 157 – 158 °C. **^{1}H NMR** (400 MHz, DMSO-d_6) δ 8.02 (s, 1H), 7.88 (d, J = 7.8 Hz, 1H), 7.82 (d, J = 7.9 Hz, 1H), 7.59 (t, J = 7.8 Hz, 2H), 7.53 – 7.47 (m, 2H), 7.40 – 7.31 (m, 6H), 7.07 – 6.99 (m, 2H); **^{13}C NMR** (101 MHz, $CDCl_3$) δ 151.0, 147.3, 135.9, 133.5, 132.6, 131.5, 130.9, 130.4, 130.2, 128.9, 128.7, 127.1, 126.5, 123.5, 122.8, 122.7, 121.4, 121.3, 117.5, 112.3, 112.2, 86.8, 50.7; **HRMS** (ESI-TOF) *m/z*: [M+H]$^+$ Calcd for: $C_{26}H_{16}BrN_2O$ 451.0441; found 451.0441.

1-(3,4-Dichlorophenyl)-3a-phenylcyclopenta[*b*]chromene-3,3(3a*H*)-dicarbonitrile (product 3af)

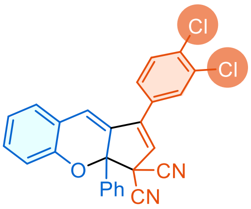

This compound was purified by column chromatography (ethyl acetate/petroleum ether = 1:10, R_f = 0.6) to afford a yellow solid in 72% yield (63 mg); m.p. 107 – 108 °C. **^{1}H NMR** (400 MHz, DMSO-d_6) δ 8.12 (d, J = 2.0 Hz, 1H), 7.93 – 7.82 (m, 2H), 7.55 – 7.47 (m, 2H), 7.40 – 7.36 (m, 4H), 7.36 – 7.27 (m, 3H), 7.08 – 7.00 (m, 2H); **^{13}C NMR** (101 MHz, DMSO-d_6) δ 151.0, 146.5, 135.8, 134.1, 133.4, 132.3, 131.6, 131.0, 130.8, 130.2, 129.8, 128.9, 128.7, 128.3, 126.6, 123.5, 123.2, 121.5, 121.4, 117.5, 112.2, 112.1, 86.8, 50.7; **HRMS** (ESI-TOF) *m/z*: $[M+H]^+$ Calcd for: $C_{26}H_{15}Cl_2N_2O$ 441.0556; found 441.0558.

1-(Naphthalen-1-yl)-3a-phenylcyclopenta[*b*]chromene-3,3(3a*H*)-dicarbonitrile (product 3ag)

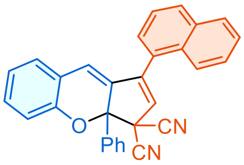

This compound was purified by column chromatography (ethyl acetate/petroleum ether = 1:10, R_f = 0.6) to afford a yellow solid in 67% yield (57 mg); m.p. 206 – 207 °C. **^{1}H NMR** (400 MHz, DMSO-d_6) δ 8.51 (s, 1H), 8.22 – 8.13 (m, 2H), 8.09 – 8.02 (m, 1H), 7.91 (dd, J = 8.5 Hz, 1.8 Hz, 1H), 7.69 – 7.63 (m, 2H), 7.61 – 7.54 (m, 2H), 7.45 – 7.36 (m, 6H), 7.32 (td, J = 8.2, 7.7, 1.6 Hz, 1H), 7.10 – 7.01 (m, 2H); **^{13}C NMR** (101 MHz, DMSO-d_6) δ 151.1, 148.8, 136.1, 134.9, 133.7, 132.9, 130.8, 130.1, 129.2, 128.9, 128.9, 128.7, 127.9, 127.7, 127.6, 127.0, 126.6, 125.0, 123.4, 121.8, 121.5, 121.4, 117.5, 112.5, 112.5, 86.9, 50.8; **HRMS** (ESI-TOF) *m/z*: $[M+H]^+$ Calcd for: $C_{30}H_{19}N_2O$ 423.1492; found 423.1490.

Methyl 3-cyano-1,3a-diphenyl-3,3a-dihydrocyclopenta[*b*]chromene-3-carboxylate (product 5a)

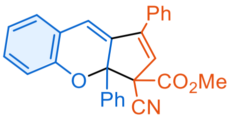

This compound was purified by column chromatography (ethyl acetate/petroleum ether = 1:10, R_f = 0.5) to afford a white solid in 59% yield (48 mg); *dr* > 19:1; m.p. 134 – 135 °C. **^{1}H NMR** (500 MHz, $CDCl_3$) δ 7.76 – 7.70 (m, 2H), 7.61 – 7.50 (m, 5H), 7.36 – 7.29 (m, 3H), 7.20 – 7.12 (m, 1H), 7.09 – 7.02 (m, 2H), 6.89 (m, 1H), 6.80 (s, 1H), 6.12 (s, 1H), 3.88 (s, 3H); **^{13}C NMR** (101 MHz, $CDCl_3$) δ 165.4, 151.7, 149.7, 138.6, 138.1, 132.2, 130.0, 129.9, 129.5, 129.3, 128.4, 128.1, 127.9, 126.7, 125.3, 122.9, 122.4, 118.6, 117.5, 116.1, 88.3, 64.3, 54.2; **HRMS** (ESI-TOF) *m/z*: $[M+H]^+$ Calcd for: $C_{27}H_{20}NO_3$ 406.1438; found 406.1430.

Ethyl 3-cyano-1,3a-diphenyl-3,3a-dihydrocyclopenta[*b*]chromene-3-carboxylate (product 5b)

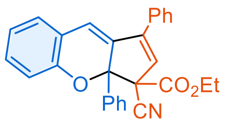

This compound was purified by column chromatography (ethyl acetate/petroleum ether = 1:12, R_f = 0.5) to afford a white solid in 81% yield (68 mg); *dr* = 1:1; m.p. 178 – 179 °C. **^{1}H NMR** (400 MHz, DMSO-d_6) δ 7.90 – 7.82 (m, 2H), 7.81 – 7.77 (m, 2H), 7.68 – 7.54 (m, 6H), 7.49 (d, *J* = 2.3 Hz, 2H), 7.39 – 7.34 (m, 4H), 7.33 – 7.30 (m, 1H), 7.30 – 7.25 (m, 5H), 7.24 (s, 1H), 7.23 – 7.18 (m, 1H), 7.12 (d, *J* = 5.1 Hz, 2H), 7.00 (s, 1H), 6.99 – 6.89 (m, 2H), 6.69 (s, 1H), 6.65 (s, 1H), 4.27 (q, *J* = 7.2 Hz, 2H), 3.72 (m, 2H),1.17 (t, *J* = 7.1 Hz, 3H), 0.89 (t, *J* = 7.1 Hz, 3H); **^{13}C NMR** (101 MHz, DMSO-d_6) δ 164.5, 164.1, 151.4, 150.8, 148.2, 146.4, 138.6, 137.9, 137.8, 136.6, 131.5, 131.2, 130.1, 130.0, 129.9, 129.4, 129.3, 129.3, 129.3, 128.4 (2C), 128.3, 128.0, 127.7, 127.6, 126.9, 126.3, 126.2, 123.0, 122.8, 122.0 (2C), 118.8, 117.6, 117.5, 117.0, 116.0, 115.7, 87.5, 86.8, 64.0, 63.0, 62.8, 62.8, 14.0, 13.4; **HRMS** (ESI-TOF) *m/z*: $[M+H]^+$ Calcd for: $C_{28}H_{22}NO_3$ 420.1594; found 420.1595.

Isopropyl 3-cyano-1,3a-diphenyl-3,3a-dihydrocyclopenta[*b*]chromene-3-carboxylate (product 5c)

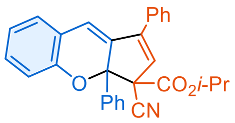

This compound was purified by column chromatography (ethyl acetate/petroleum ether = 1:10, R_f = 0.5) to afford a white solid in 68% yield (59 mg); *dr* = 1.3:1; m.p. 130 – 131 °C. **^{1}H NMR** (500 MHz, $CDCl_3$) δ 7.72 (m, 3.6H), 7.62 – 7.45 (m, 9H), 7.35 – 7.26 (m, 3.6H), 7.20 (dd, *J* = 5.2, 2.3 Hz, 3.6H), 7.17 – 7.12 (m, 0.8H), 7.08 – 7.01 (m, 2.7H), 6.90 (m, 1.8H), 6.82 (s, 1H), 6.78 (s, 0.8H), 6.30 (s, 1H), 6.12 (s, 0.8H), 5.15 (p, *J* = 6.2 Hz, 0.8H), 4.63 (p, *J* = 6.3 Hz, 1H), 1.35 (d, *J* = 6.3 Hz, 2.4H), 1.25 (d, *J* = 6.3 Hz, 2.4H), 1.11 (d, *J* = 6.3 Hz, 3H), 0.94 (d, *J* = 6.3 Hz, 3H); **^{13}C NMR** (126 MHz, $CDCl_3$) δ 164.5, 164.2, 152.4, 151.6, 149.6, 147.9, 138.8, 138.5, 138.2, 137.1, 132.4, 132.2, 130.0, 129.9 (2C), 129.5, 129.3 (2C), 129.2, 128.4, 128.1 (2C), 127.9, 127.8, 127.4, 126.7, 125.61, 125.5, 122.8, 122.3, 122.1, 119.1, 118.2, 118.0, 117.2, 115.8, 87.3, 71.6 (2C), 64.5, 63.3, 21.9, 21.6, 21.5, 21.2; **HRMS** (ESI-TOF) *m/z*: $[M+H]^+$ Calcd for: $C_{29}H_{24}NO_3$ 434.1751; found 434.1738.

2-Methyl-1,3a-diphenylcyclopenta[*b*]chromene-3,3(3a*H*)-dicarbonitrile (product 7)

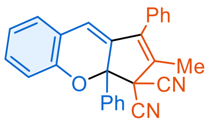

This compound was purified by column chromatography (ethyl acetate/petroleum ether = 1:10, R_f = 0.6) to afford a white solid in 47% yield (36 mg); m.p.153 – 154 °C. **^{1}H NMR** (500 MHz, $CDCl_3$) δ 7.61 – 7.51 (m, 7H), 7.34 (dd, *J* = 5.1 Hz, 1.9 Hz, 3H), 7.21 (d, *J* = 3.6 Hz, 2H), 7.03 (d, *J* = 7.5 Hz, 1H), 6.96 – 6.89 (m, 1H), 6.60 (s, 1H), 2.19 (s, 3H); **^{13}C NMR** (101 MHz, $CDCl_3$) δ 151.9, 144.2, 138.0, 137.0, 131.8, 131.3, 130.2, 130.1, 129.6, 129.3, 128.9, 128.7, 128.2, 126.8, 123.2, 122.2, 118.8, 117.8, 112.2, 111.9, 85.7, 56.4, 13.0; **HRMS** (ESI-TOF) *m/z*: $[M+H]^+$ Calcd for: $C_{27}H_{19}N_2O$ 387.1492; found 387.1495.

2,9-Dibromo-1,3a-diphenylcyclopenta[*b*]chromene-3,3(3a*H*)-dicarbonitrile (product 8)

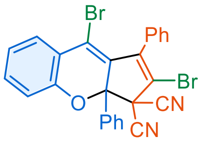

This compound was purified by column chromatography (ethyl acetate/petroleum ether = 1:8, R_f = 0.6) to afford a yellow solid in 85% yield (90 mg); m.p. 175 – 176 °C. **^{1}H NMR** (400 MHz, DMSO-d_6) *δ* 7.79 (d, *J* = 8.4 Hz, 1H), 7.76 (d, *J* = 7.8 Hz, 1H), 7.71 – 7.65 (m, 1H), 7.63 – 7.59 (m, 3H), 7.53 – 7.50 (m, 1H), 7.47 (s, 1H), 7.35 (d, *J* = 7.5 Hz, 1H), 7.31 – 7.28 (m, 2H), 7.17 (d, *J* = 1.0 Hz, 1H), 7.01 (dd, *J* = 6.7, 2.9 Hz, 2H); **^{13}C NMR** (101 MHz, DMSO-d_6) *δ* 157.7, 156.7, 156.0, 154.1, 150.4, 136.7, 129.8, 129.0, 128.8, 128.7, 128.3, 127.9, 127.8, 127.8, 124.0, 123.6, 123.1, 117.6, 113.9, 112.0, 108.4, 94.2; **HRMS** (ESI-TOF) *m/z*: $[M+H]^+$ Calcd for: $C_{26}H_{15}Br_2N_2O$ 528.9546; found 528.9549.

2-Bromo-9-hydroxy-1,3a-diphenyl-9,9a-dihydrocyclopenta[*b*]chromene-3,3(3a*H*)-dicarbonitrile (product 9)

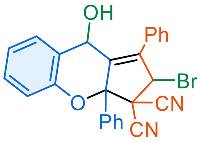

This compound was purified by column chromatography (ethyl acetate/petroleum ether = 1:10, R_f = 0.6) to afford a white solid in 68% yield (64 mg); 159-160 °C. **^{1}H NMR** (400 MHz, $CDCl_3$) *δ* 7.85 – 7.76 (m, 4H), 7.58 (q, *J* = 8.4, 7.4 Hz, 3H), 7.49 – 7.41 (m, 3H), 7.30 (t, *J* = 7.1 Hz, 2H), 7.18 (d, *J* = 8.7 Hz, 1H), 7.05 – 6.96 (m, 1H), 5.83 (s, 1H), 5.18 (d, *J* = 9.9 Hz, 1H), 1.88 (d, *J* = 9.9 Hz, 1H). **^{13}C NMR** (101 MHz, $CDCl_3$) *δ* 150.7, 144.0, 135.7, 134.6, 131.0, 130.9, 130.8, 130.6, 130.3, 129.5, 129.1, 128.8, 128.7, 123.4, 122.3, 118.4, 112.6, 111.5, 91.4, 61.7, 53.6, 53.5. **HRMS** (ESI-TOF) *m/z*: $[M–H]^+$ Calcd for: $C_{26}H_{18}BrN_2O_2$ 469.0546; Found 469.0538.

4-(2-Benzyl-5-phenylfuran-3-yl)phenol (product 13)

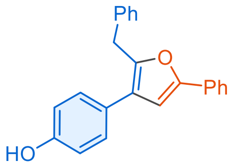

This compound was purified by column chromatography (ethyl acetate/petroleum ether = 1:8, R_f = 0.5) to afford a white solid in 46% yield (30 mg); m.p. 137 – 138 °C. **^{1}H NMR** (500 MHz, $CDCl_3$) δ 7.69 – 7.64 (m, 2H), 7.40 – 7.34 (m, 3H), 7.32 – 7.27 (m, 5H), 7.25 – 7.21 (m, 2H), 6.88 – 6.84 (m, 2H), 6.78 (s, 1H), 4.87 (s, 1H), 4.17 (s, 2H); **^{13}C NMR** (101 MHz, $CDCl_3$) δ 154.66, 152.38, 148.53, 138.77, 130.90, 129.15, 128.77, 128.74, 128.45, 127.29, 126.55, 126.51, 124.16, 123.69, 115.70, 106.82, 33.02; **HRMS** (ESI-TOF) *m/z*: $[M+H]^+$ Calcd for: $C_{23}H_{19}O_2$ 327.1380; found 327.1381.

14. ^{1}H, ^{13}C, and ^{19}F NMR Spectra

1,3a-Diphenylcyclopenta[*b*]chromene-3,3(3a*H*)-dicarbonitrile (product 3aa)

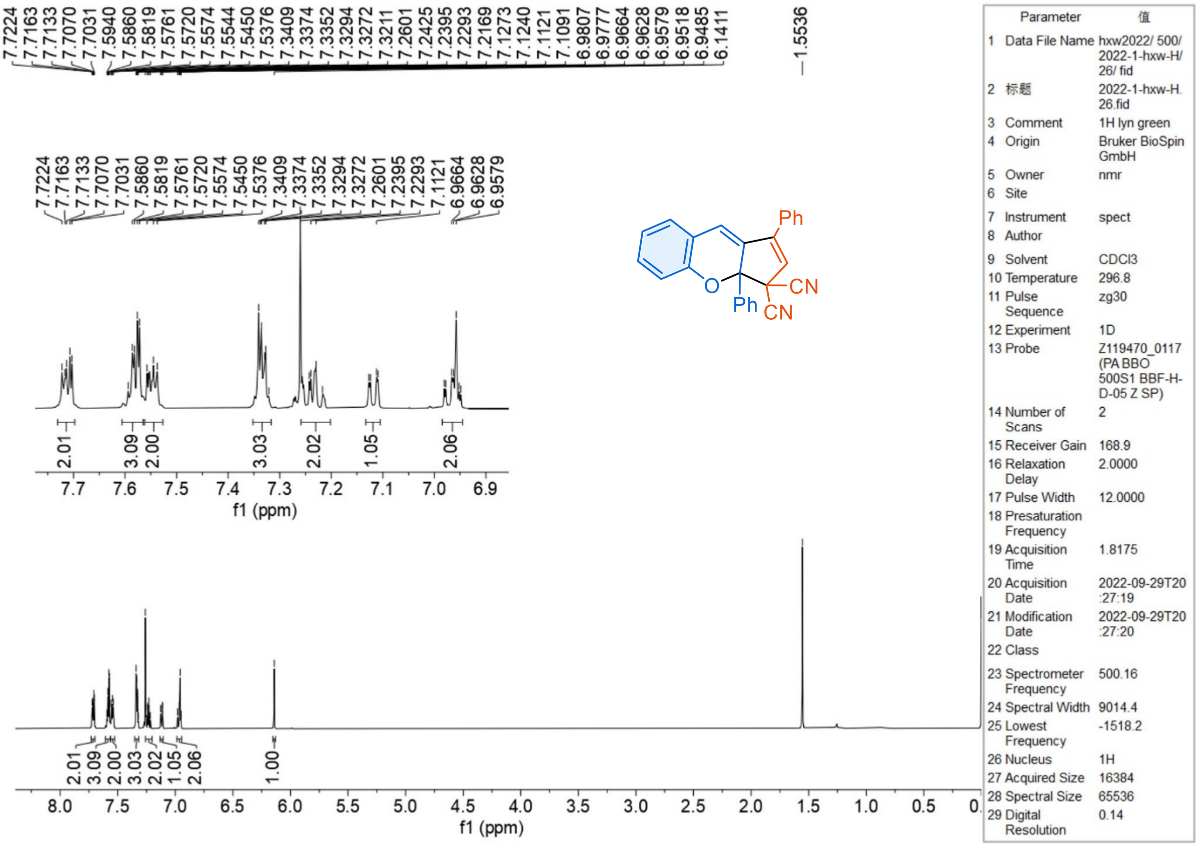

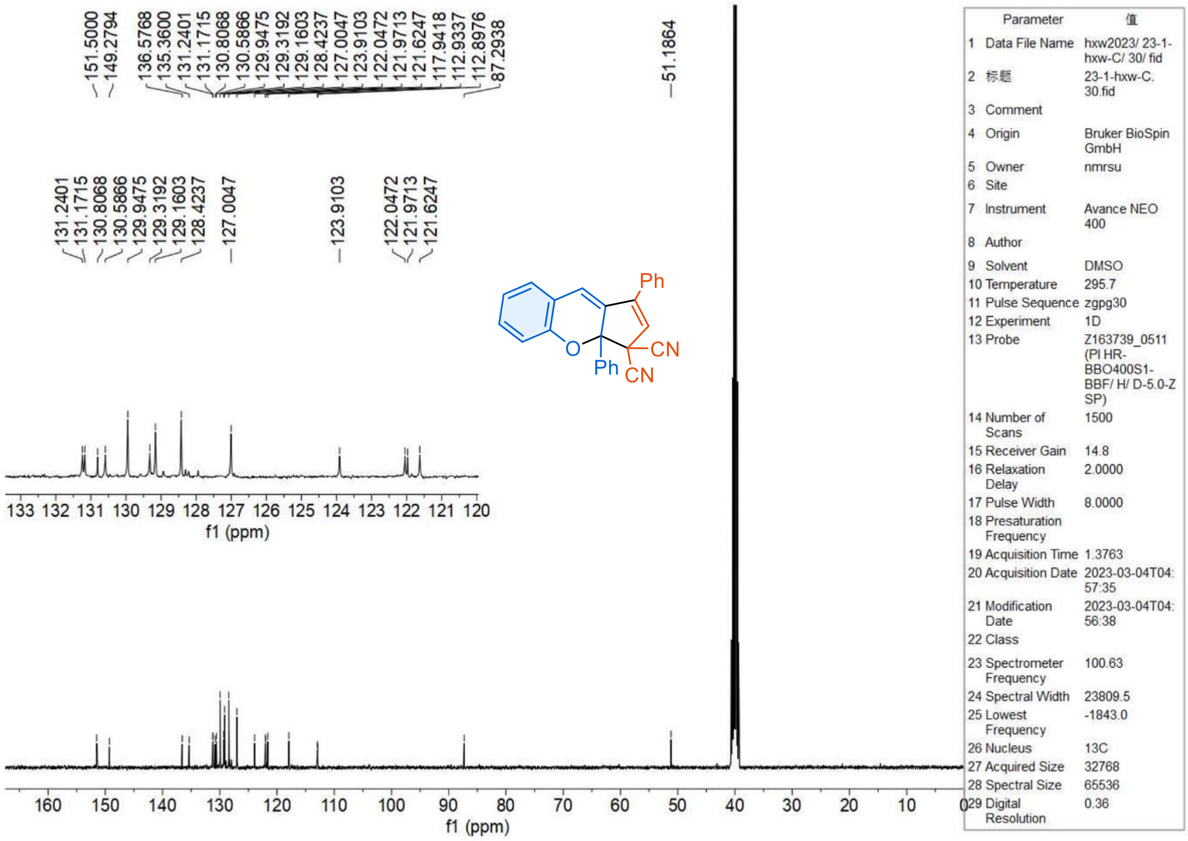

7-Methyl-1,3a-diphenylcyclopenta[*b*]chromene-3,3(3a*H*)-dicarbonitrile (product 3ba)

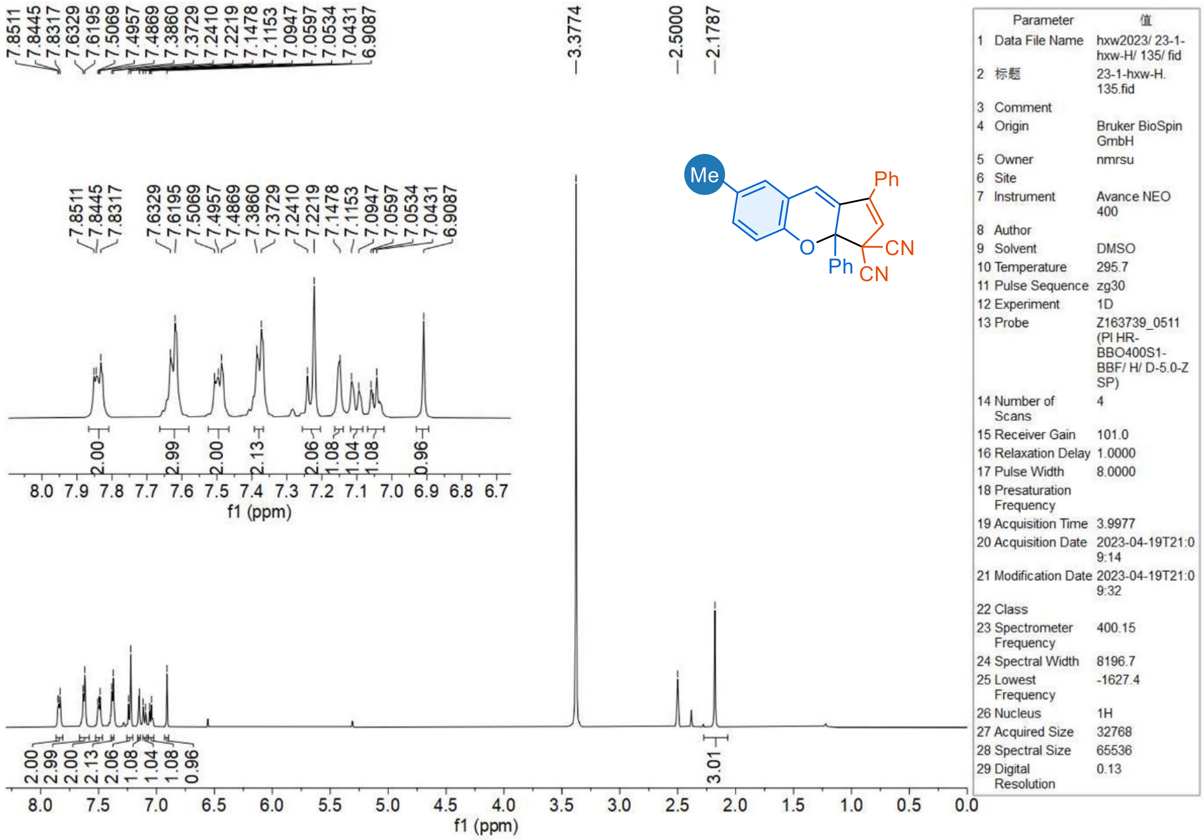

Parameter	值
1 Data File Name	hxw2023/ 23-1-hxw-H/ 135/ fid
2 标题	23-1-hxw-H.135.fid
3 Comment	
4 Origin	Bruker BioSpin GmbH
5 Owner	nmrsu
6 Site	
7 Instrument	Avance NEO 400
8 Author	
9 Solvent	DMSO
10 Temperature	295.7
11 Pulse Sequence	zg30
12 Experiment	1D
13 Probe	Z163739_0511 (PI HR-BBO400S1-BBF/ H/ D-5.0-Z SP)
14 Number of Scans	4
15 Receiver Gain	101.0
16 Relaxation Delay	1.0000
17 Pulse Width	8.0000
18 Presaturation Frequency	
19 Acquisition Time	3.9977
20 Acquisition Date	2023-04-19T21:09:14
21 Modification Date	2023-04-19T21:09:32
22 Class	
23 Spectrometer Frequency	400.15
24 Spectral Width	8196.7
25 Lowest Frequency	-1627.4
26 Nucleus	1H
27 Acquired Size	32768
28 Spectral Size	65536
29 Digital Resolution	0.13

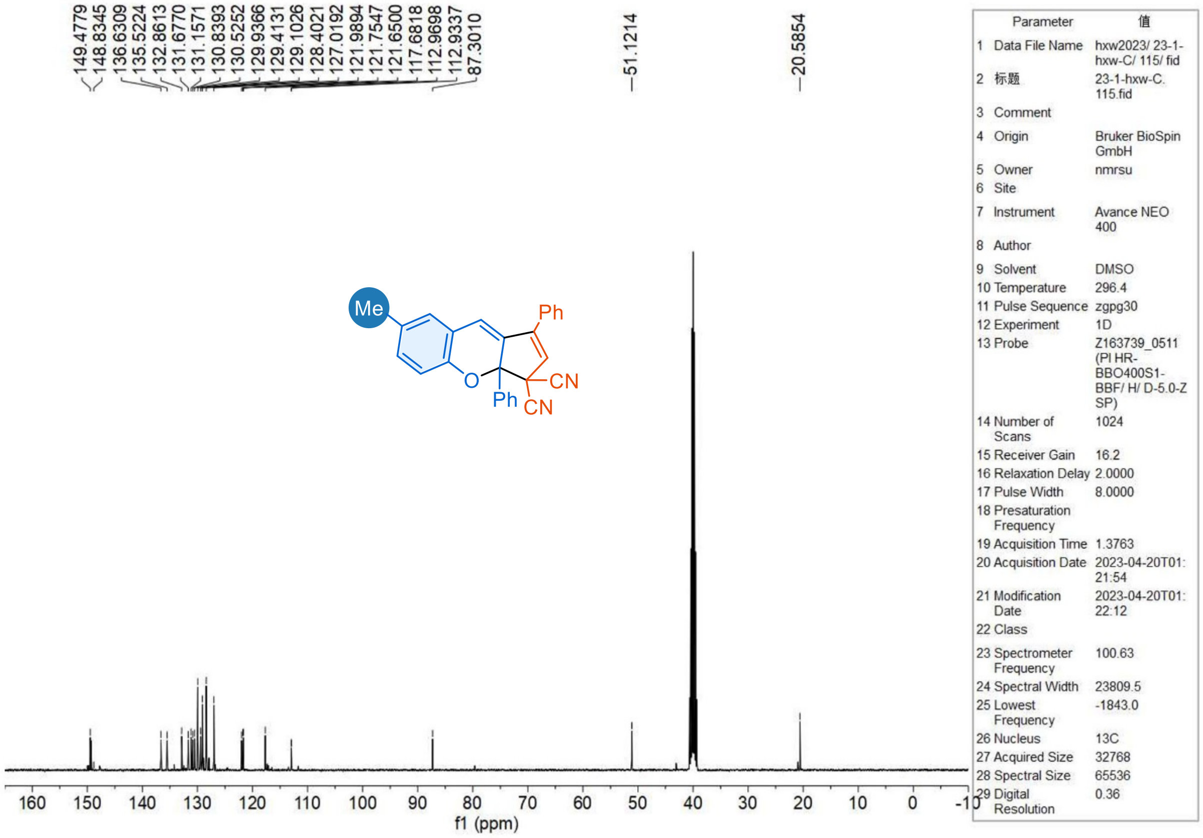

Parameter	值
1 Data File Name	hxw2023/ 23-1-hxw-C/ 115/ fid
2 标题	23-1-hxw-C.115.fid
3 Comment	
4 Origin	Bruker BioSpin GmbH
5 Owner	nmrsu
6 Site	
7 Instrument	Avance NEO 400
8 Author	
9 Solvent	DMSO
10 Temperature	296.4
11 Pulse Sequence	zgpg30
12 Experiment	1D
13 Probe	Z163739_0511 (PI HR-BBO400S1-BBF/ H/ D-5.0-Z SP)
14 Number of Scans	1024
15 Receiver Gain	16.2
16 Relaxation Delay	2.0000
17 Pulse Width	8.0000
18 Presaturation Frequency	
19 Acquisition Time	1.3763
20 Acquisition Date	2023-04-20T01:21:54
21 Modification Date	2023-04-20T01:22:12
22 Class	
23 Spectrometer Frequency	100.63
24 Spectral Width	23809.5
25 Lowest Frequency	-1843.0
26 Nucleus	13C
27 Acquired Size	32768
28 Spectral Size	65536
29 Digital Resolution	0.36

7-Bromo-1,3a-diphenylcyclopenta[*b*]chromene-3,3(3a*H*)-dicarbonitrile (product 3ca)

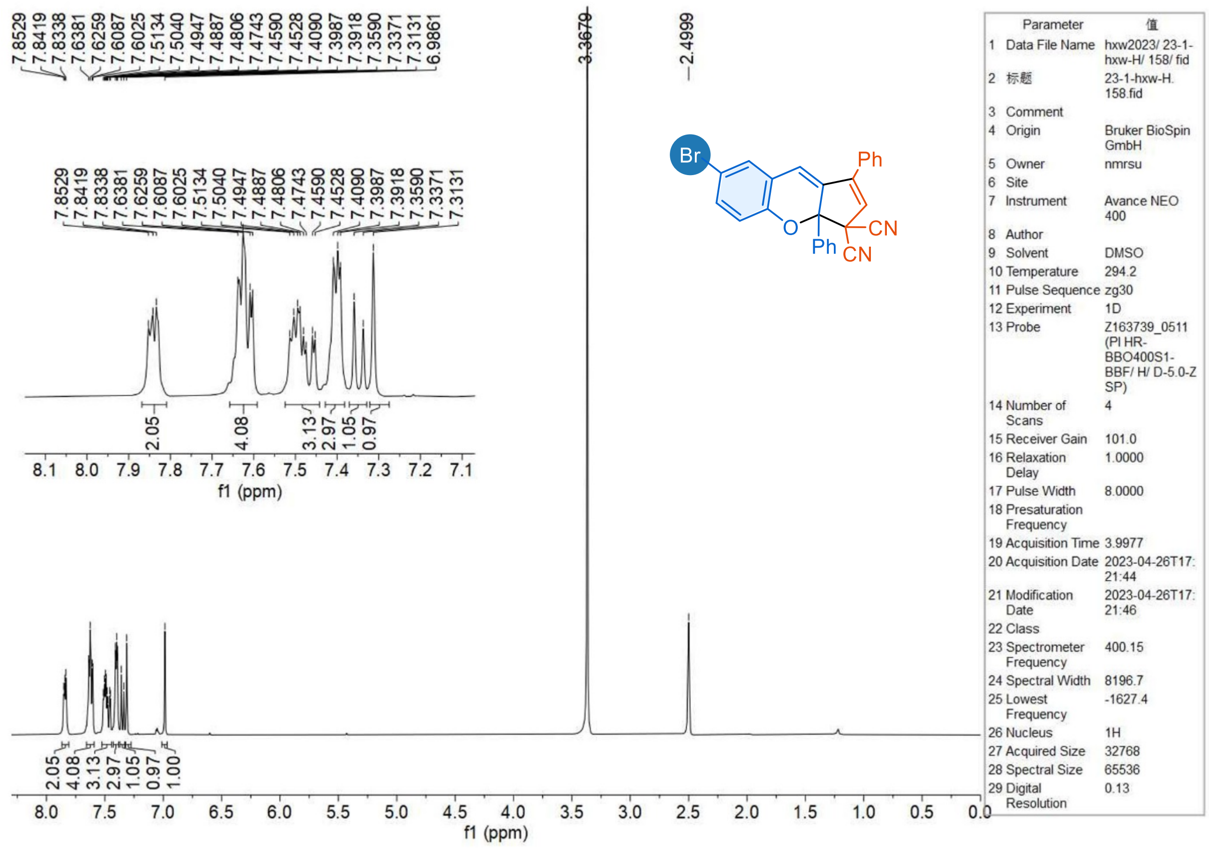

Parameter	值
1 Data File Name	hxw2023/ 23-1-hxw-H/ 158/ fid
2 标题	23-1-hxw-H. 158.fid
3 Comment	
4 Origin	Bruker BioSpin GmbH
5 Owner	nmrsu
6 Site	
7 Instrument	Avance NEO 400
8 Author	
9 Solvent	DMSO
10 Temperature	294.2
11 Pulse Sequence	zg30
12 Experiment	1D
13 Probe	Z163739_0511 (PI HR-BBO400S1-BBF/ H/ D-5.0-Z SP)
14 Number of Scans	4
15 Receiver Gain	101.0
16 Relaxation Delay	1.0000
17 Pulse Width	8.0000
18 Presaturation Frequency	
19 Acquisition Time	3.9977
20 Acquisition Date	2023-04-26T17:21:44
21 Modification Date	2023-04-26T17:21:46
22 Class	
23 Spectrometer Frequency	400.15
24 Spectral Width	8196.7
25 Lowest Frequency	-1627.4
26 Nucleus	1H
27 Acquired Size	32768
28 Spectral Size	65536
29 Digital Resolution	0.13

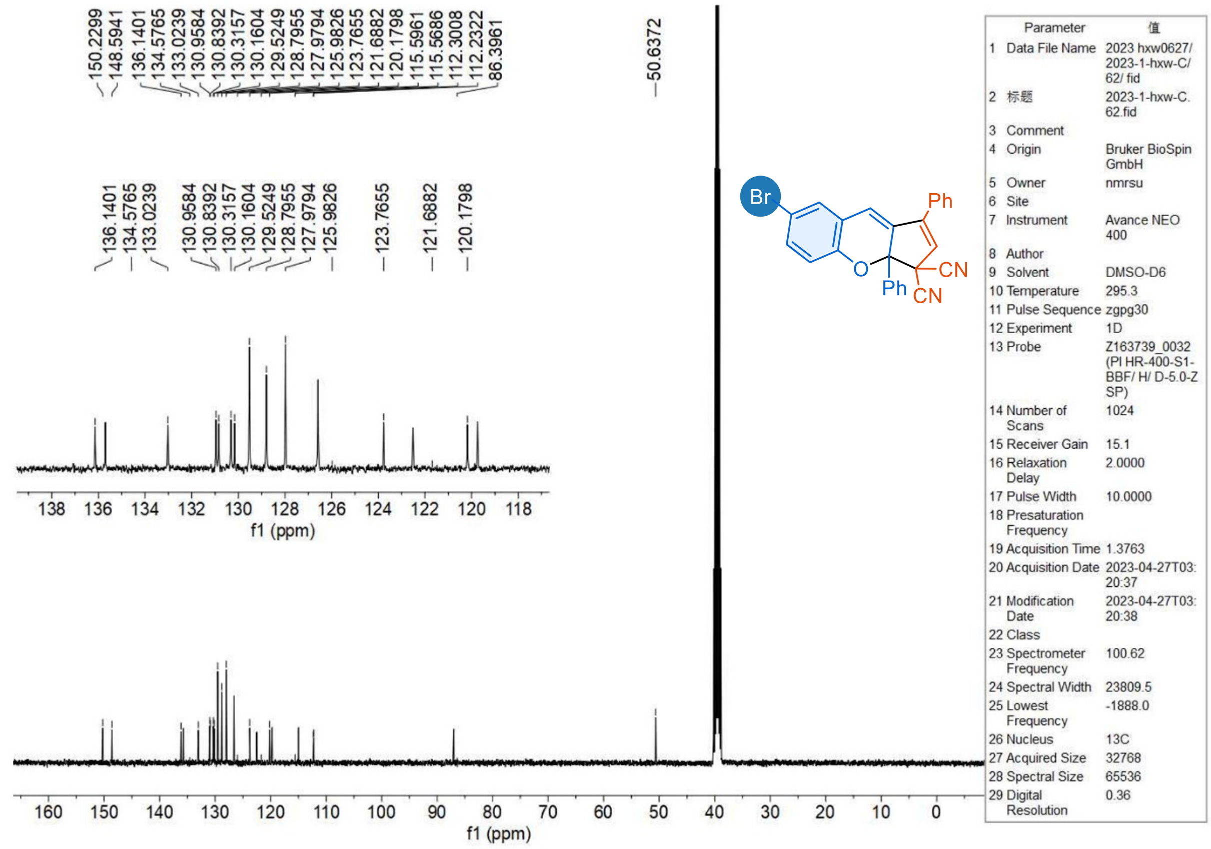

Parameter	值
1 Data File Name	2023 hxw0627/ 2023-1-hxw-C/ 62/ fid
2 标题	2023-1-hxw-C. 62.fid
3 Comment	
4 Origin	Bruker BioSpin GmbH
5 Owner	nmrsu
6 Site	
7 Instrument	Avance NEO 400
8 Author	
9 Solvent	DMSO-D6
10 Temperature	295.3
11 Pulse Sequence	zgpg30
12 Experiment	1D
13 Probe	Z163739_0032 (PI HR-400-S1-BBF/ H/ D-5.0-Z SP)
14 Number of Scans	1024
15 Receiver Gain	15.1
16 Relaxation Delay	2.0000
17 Pulse Width	10.0000
18 Presaturation Frequency	
19 Acquisition Time	1.3763
20 Acquisition Date	2023-04-27T03:20:37
21 Modification Date	2023-04-27T03:20:38
22 Class	
23 Spectrometer Frequency	100.62
24 Spectral Width	23809.5
25 Lowest Frequency	-1888.0
26 Nucleus	13C
27 Acquired Size	32768
28 Spectral Size	65536
29 Digital Resolution	0.36

7-Chloro-1,3a-diphenylcyclopenta[*b*]chromene-3,3(3a*H*)-dicarbonitrile (product 3da)

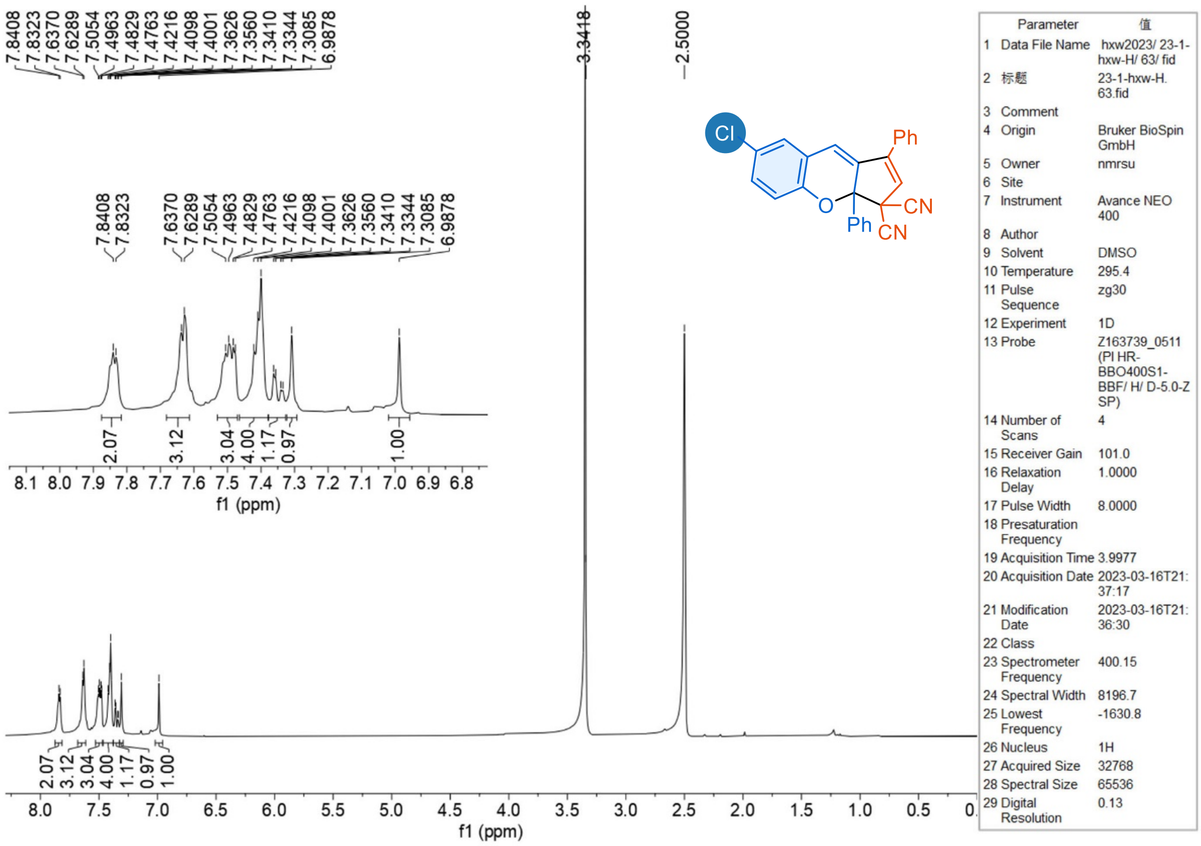

	Parameter	值
1	Data File Name	hxw2023/ 23-1-hxw-H/ 63/ fid
2	标题	23-1-hxw-H.63.fid
3	Comment	
4	Origin	Bruker BioSpin GmbH
5	Owner	nmrsu
6	Site	
7	Instrument	Avance NEO 400
8	Author	
9	Solvent	DMSO
10	Temperature	295.4
11	Pulse Sequence	zg30
12	Experiment	1D
13	Probe	Z163739_0511 (PI HR-BBO400S1-BBF/ H/ D-5.0-Z SP)
14	Number of Scans	4
15	Receiver Gain	101.0
16	Relaxation Delay	1.0000
17	Pulse Width	8.0000
18	Presaturation Frequency	
19	Acquisition Time	3.9977
20	Acquisition Date	2023-03-16T21:37:17
21	Modification Date	2023-03-16T21:36:30
22	Class	
23	Spectrometer Frequency	400.15
24	Spectral Width	8196.7
25	Lowest Frequency	-1630.8
26	Nucleus	1H
27	Acquired Size	32768
28	Spectral Size	65536
29	Digital Resolution	0.13

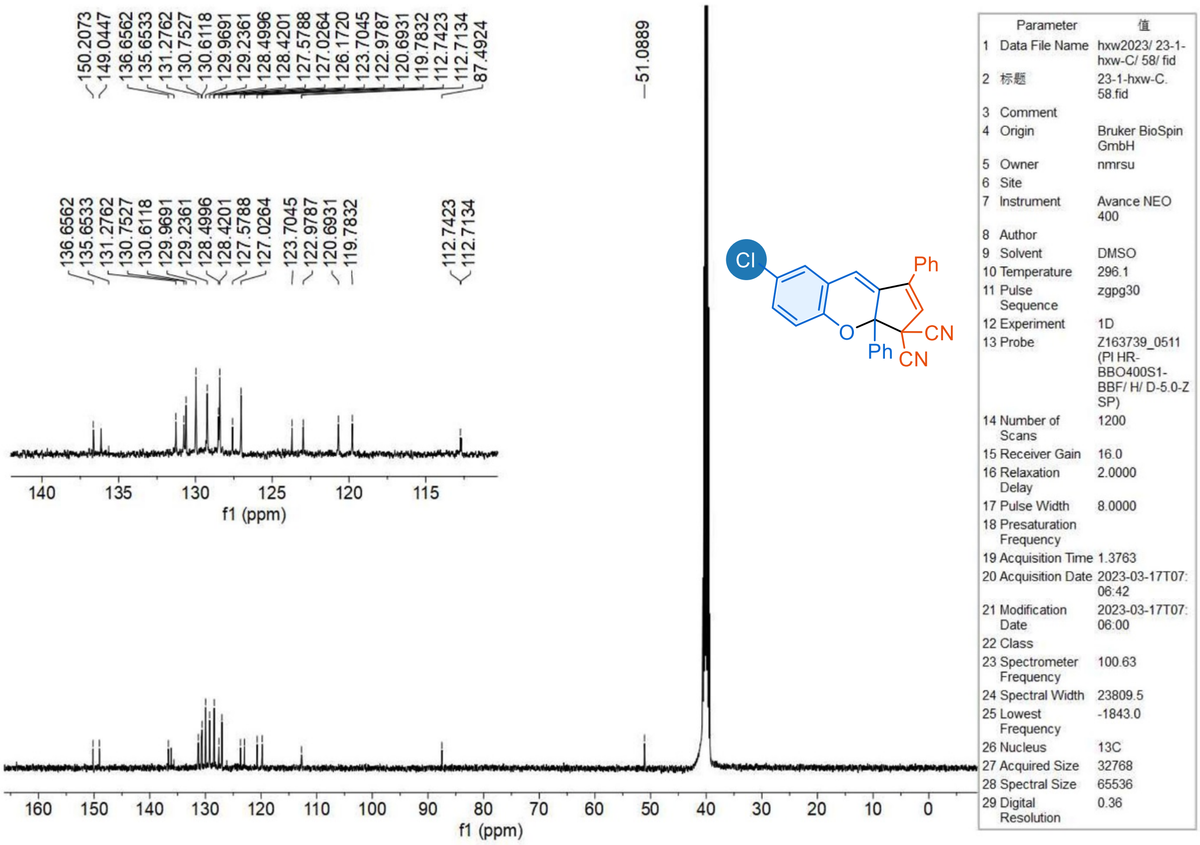

	Parameter	值
1	Data File Name	hxw2023/ 23-1-hxw-C/ 58/ fid
2	标题	23-1-hxw-C.58.fid
3	Comment	
4	Origin	Bruker BioSpin GmbH
5	Owner	nmrsu
6	Site	
7	Instrument	Avance NEO 400
8	Author	
9	Solvent	DMSO
10	Temperature	296.1
11	Pulse Sequence	zgpg30
12	Experiment	1D
13	Probe	Z163739_0511 (PI HR-BBO400S1-BBF/ H/ D-5.0-Z SP)
14	Number of Scans	1200
15	Receiver Gain	16.0
16	Relaxation Delay	2.0000
17	Pulse Width	8.0000
18	Presaturation Frequency	
19	Acquisition Time	1.3763
20	Acquisition Date	2023-03-17T07:06:42
21	Modification Date	2023-03-17T07:06:00
22	Class	
23	Spectrometer Frequency	100.63
24	Spectral Width	23809.5
25	Lowest Frequency	-1843.0
26	Nucleus	13C
27	Acquired Size	32768
28	Spectral Size	65536
29	Digital Resolution	0.36

7-Fluoro-1,3a-diphenylcyclopenta[*b*]chromene-3,3(3a*H*)-dicarbonitrile (product 3ea)

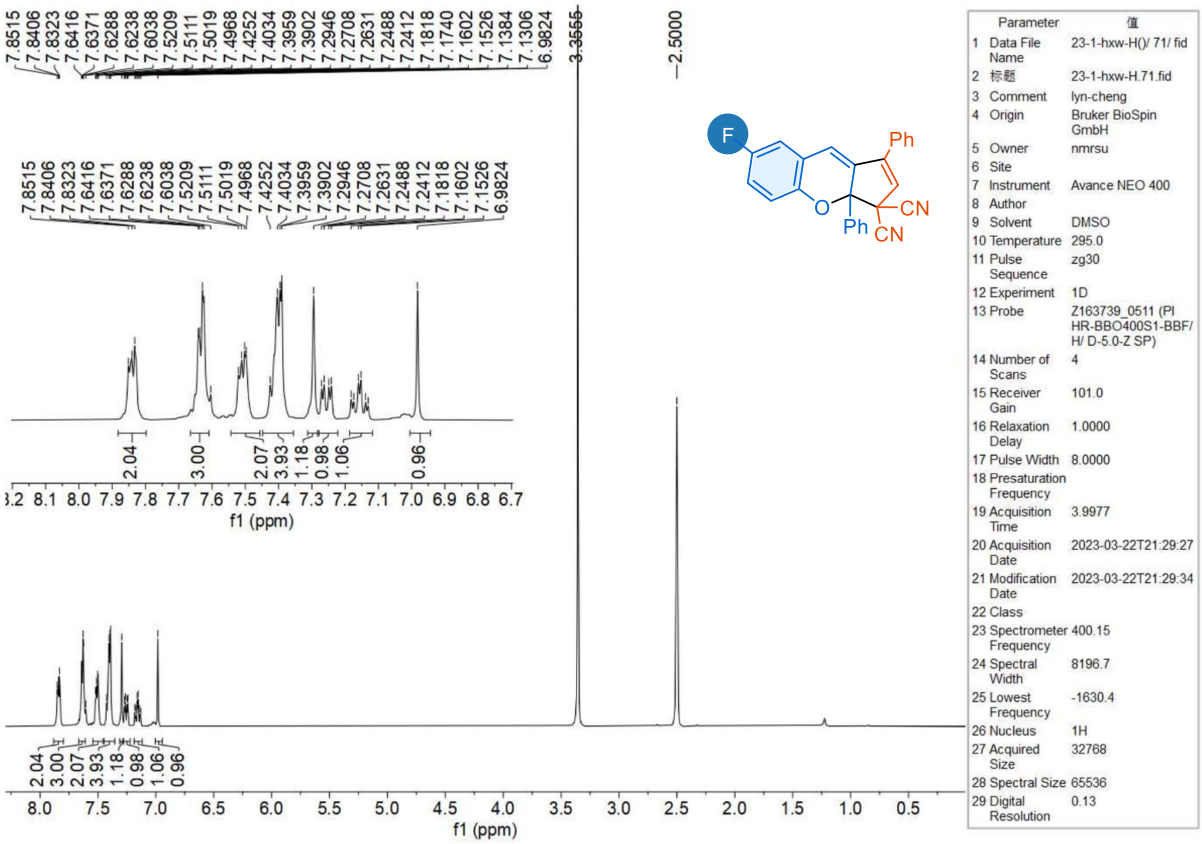

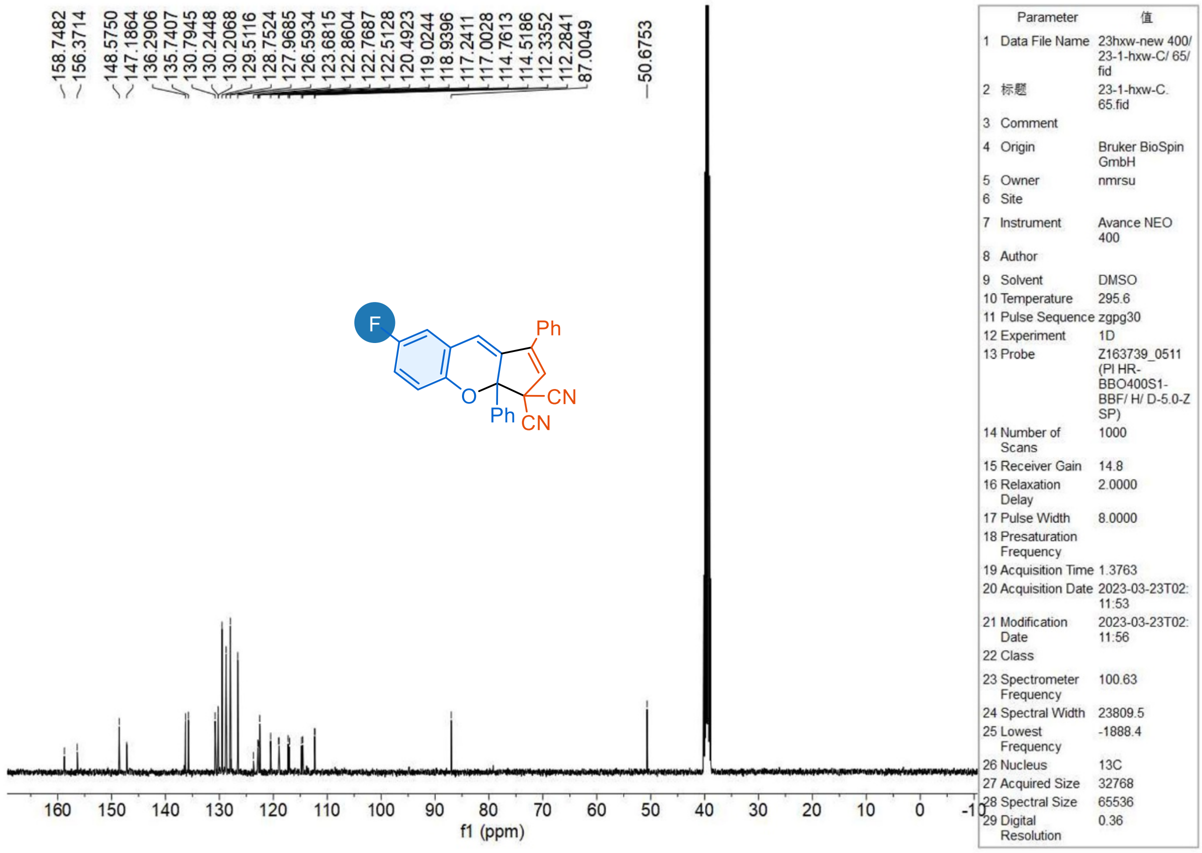

7-Fluoro-1,3a-diphenylcyclopenta[*b*]chromene-3,3(3a*H*)-dicarbonitrile (product 3ea)

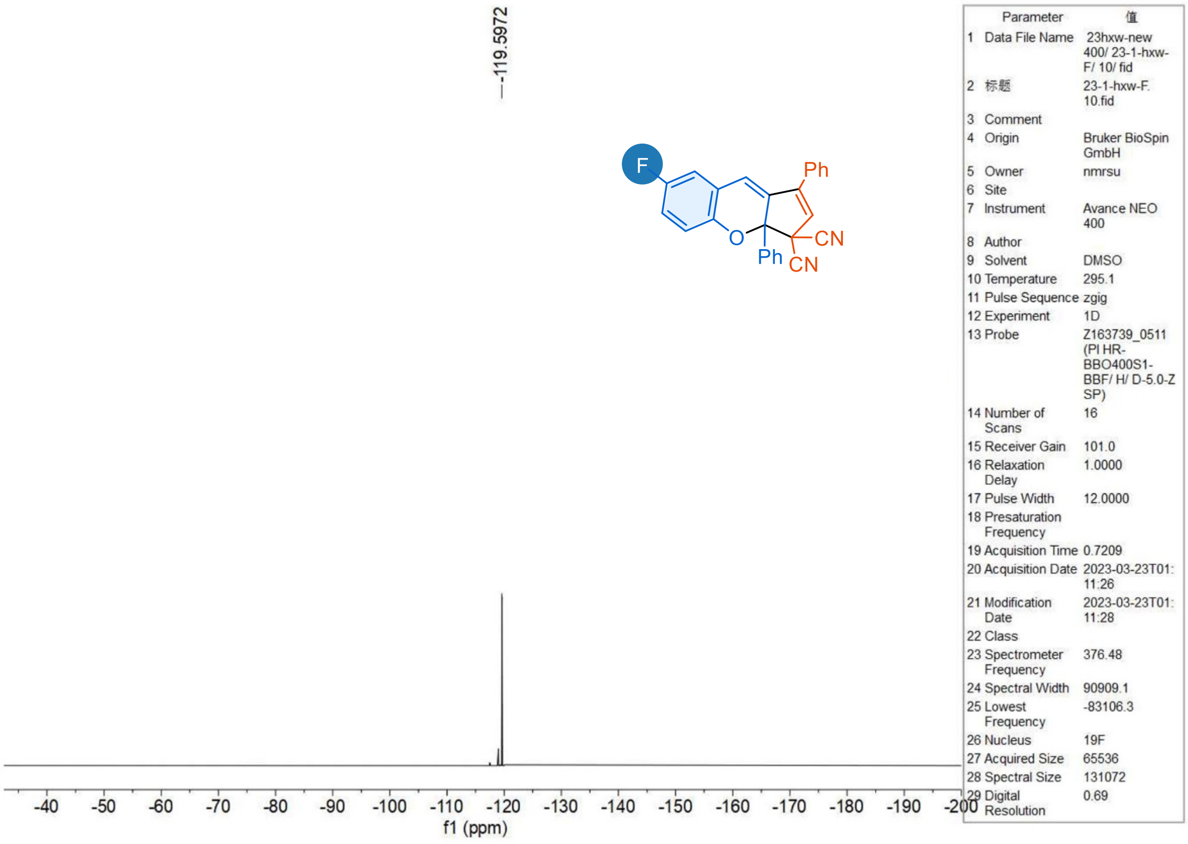

	Parameter	值
1	Data File Name	23hxw-new 400/ 23-1-hxw-F/ 10/ fid
2	标题	23-1-hxw-F. 10.fid
3	Comment	
4	Origin	Bruker BioSpin GmbH
5	Owner	nmrsu
6	Site	
7	Instrument	Avance NEO 400
8	Author	
9	Solvent	DMSO
10	Temperature	295.1
11	Pulse Sequence	zgig
12	Experiment	1D
13	Probe	Z163739_0511 (PI HR-BBO400S1-BBF/ H/ D-5.0-Z SP)
14	Number of Scans	16
15	Receiver Gain	101.0
16	Relaxation Delay	1.0000
17	Pulse Width	12.0000
18	Presaturation Frequency	
19	Acquisition Time	0.7209
20	Acquisition Date	2023-03-23T01:11:26
21	Modification Date	2023-03-23T01:11:28
22	Class	
23	Spectrometer Frequency	376.48
24	Spectral Width	90909.1
25	Lowest Frequency	-83106.3
26	Nucleus	19F
27	Acquired Size	65536
28	Spectral Size	131072
29	Digital Resolution	0.69

5-Fluoro-1,3a-diphenylcyclopenta[*b*]chromene-3,3(3a*H*)-dicarbonitrile (product 3fa)

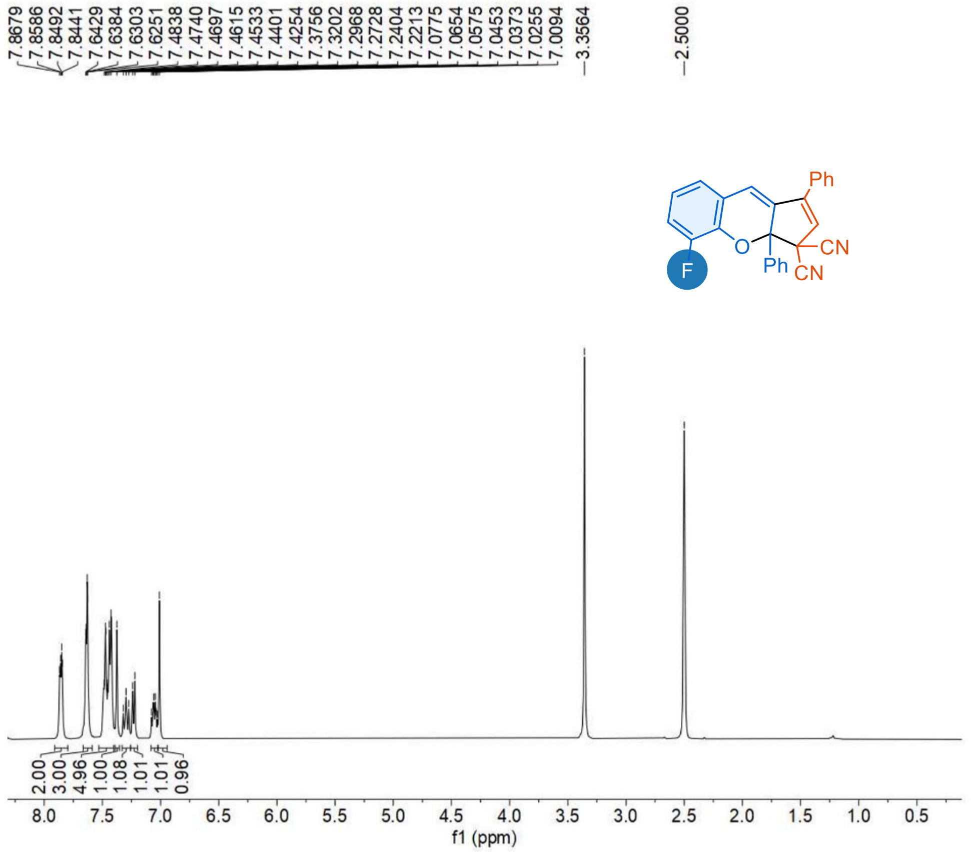

Parameter	值
1 Data File Name	2023 hxw0627/ 23-1-hxw-H/ 103/ fid
2 标题	23-1-hxw-H. 103.fid
3 Comment	
4 Origin	Bruker BioSpin GmbH
5 Owner	nmrsu
6 Site	
7 Instrument	Avance NEO 400
8 Author	
9 Solvent	DMSO
10 Temperature	295.0
11 Pulse Sequence	zg30
12 Experiment	1D
13 Probe	Z163739_0511 (PI HR-BBO400S1-BBF/ H/ D-5.0-Z SP)
14 Number of Scans	4
15 Receiver Gain	101.0
16 Relaxation Delay	1.0000
17 Pulse Width	8.0000
18 Presaturation Frequency	
19 Acquisition Time	3.9977
20 Acquisition Date	2023-04-05T20:02:46
21 Modification Date	2023-04-05T20:02:52
22 Class	
23 Spectrometer Frequency	400.15
24 Spectral Width	8196.7
25 Lowest Frequency	-1630.4
26 Nucleus	1H
27 Acquired Size	32768
28 Spectral Size	65536
29 Digital Resolution	0.13

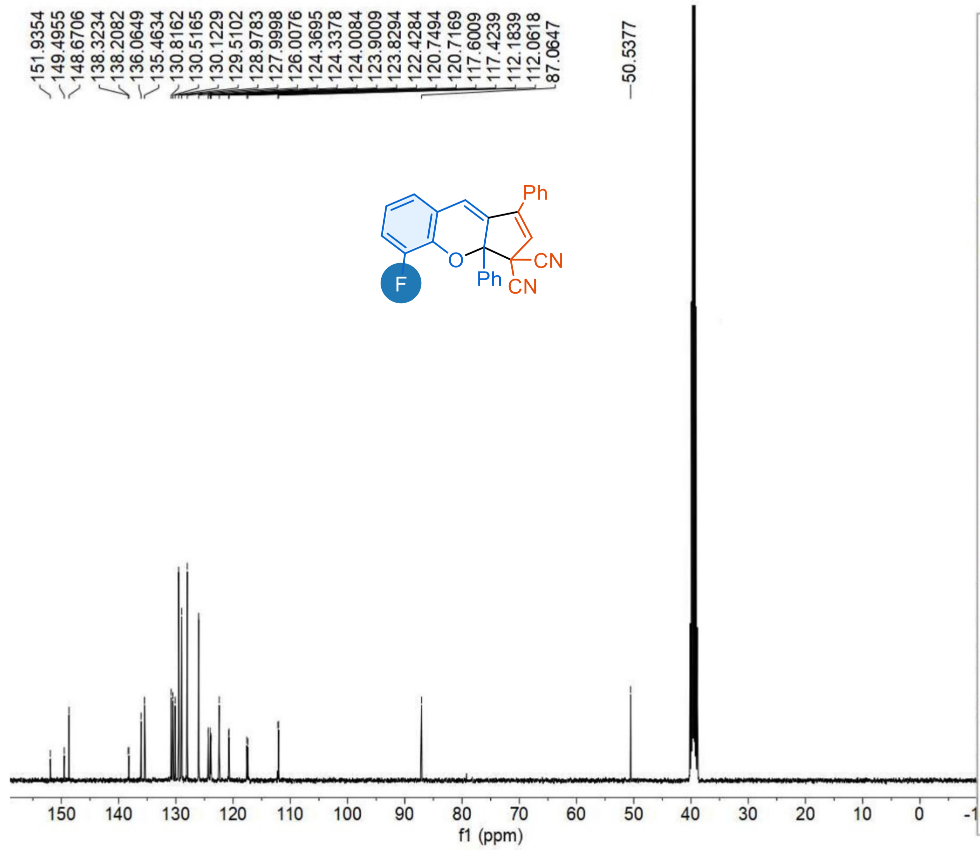

Parameter	值
1 Data File Name	23hxw-new 400/ 23-1-hxw-C/ 86/ fid
2 标题	23-1-hxw-C. 86.fid
3 Comment	
4 Origin	Bruker BioSpin GmbH
5 Owner	nmrsu
6 Site	
7 Instrument	Avance NEO 400
8 Author	
9 Solvent	DMSO
10 Temperature	295.7
11 Pulse Sequence	zgpg30
12 Experiment	1D
13 Probe	Z163739_0511 (PI HR-BBO400S1-BBF/ H/ D-5.0-Z SP)
14 Number of Scans	1024
15 Receiver Gain	16.2
16 Relaxation Delay	2.0000
17 Pulse Width	8.0000
18 Presaturation Frequency	
19 Acquisition Time	1.3763
20 Acquisition Date	2023-04-05T21:18:33
21 Modification Date	2023-04-05T21:18:40
22 Class	
23 Spectrometer Frequency	100.63
24 Spectral Width	23809.5
25 Lowest Frequency	-1889.3
26 Nucleus	13C
27 Acquired Size	32768
28 Spectral Size	65536
29 Digital Resolution	0.36

5-Fluoro-1,3a-diphenylcyclopenta[*b*]chromene-3,3(3a*H*)-dicarbonitrile (product 3fa)

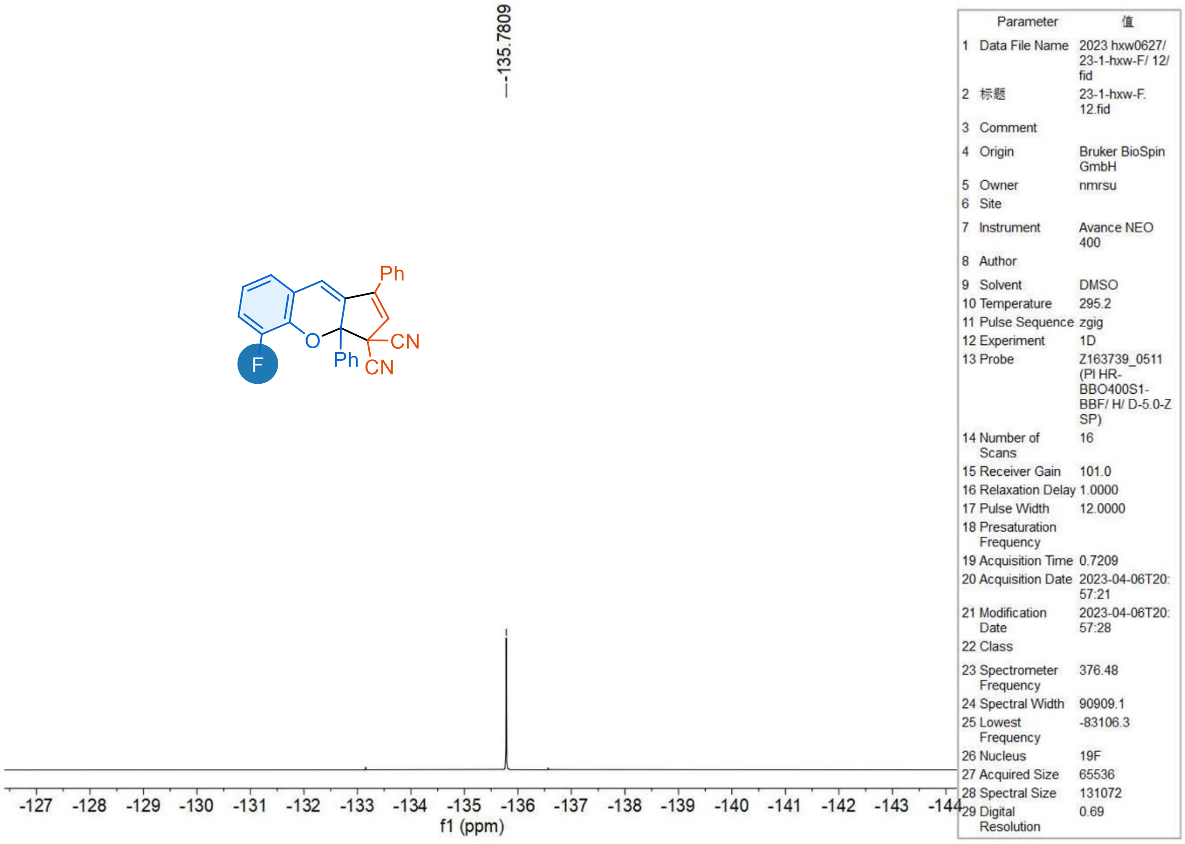

	Parameter	值
1	Data File Name	2023 hxw0627/ 23-1-hxw-F/ 12/ fid
2	标题	23-1-hxw-F. 12.fid
3	Comment	
4	Origin	Bruker BioSpin GmbH
5	Owner	nmrsu
6	Site	
7	Instrument	Avance NEO 400
8	Author	
9	Solvent	DMSO
10	Temperature	295.2
11	Pulse Sequence	zgig
12	Experiment	1D
13	Probe	Z163739_0511 (PI HR-BBO400S1-BBF/ H/ D-5.0-Z SP)
14	Number of Scans	16
15	Receiver Gain	101.0
16	Relaxation Delay	1.0000
17	Pulse Width	12.0000
18	Presaturation Frequency	
19	Acquisition Time	0.7209
20	Acquisition Date	2023-04-06T20:57:21
21	Modification Date	2023-04-06T20:57:28
22	Class	
23	Spectrometer Frequency	376.48
24	Spectral Width	90909.1
25	Lowest Frequency	-83106.3
26	Nucleus	19F
27	Acquired Size	65536
28	Spectral Size	131072
29	Digital Resolution	0.69

5-Bromo-1,3a-diphenylcyclopenta[*b*]chromene-3,3(3a*H*)-dicarbonitrile (product 3ga)

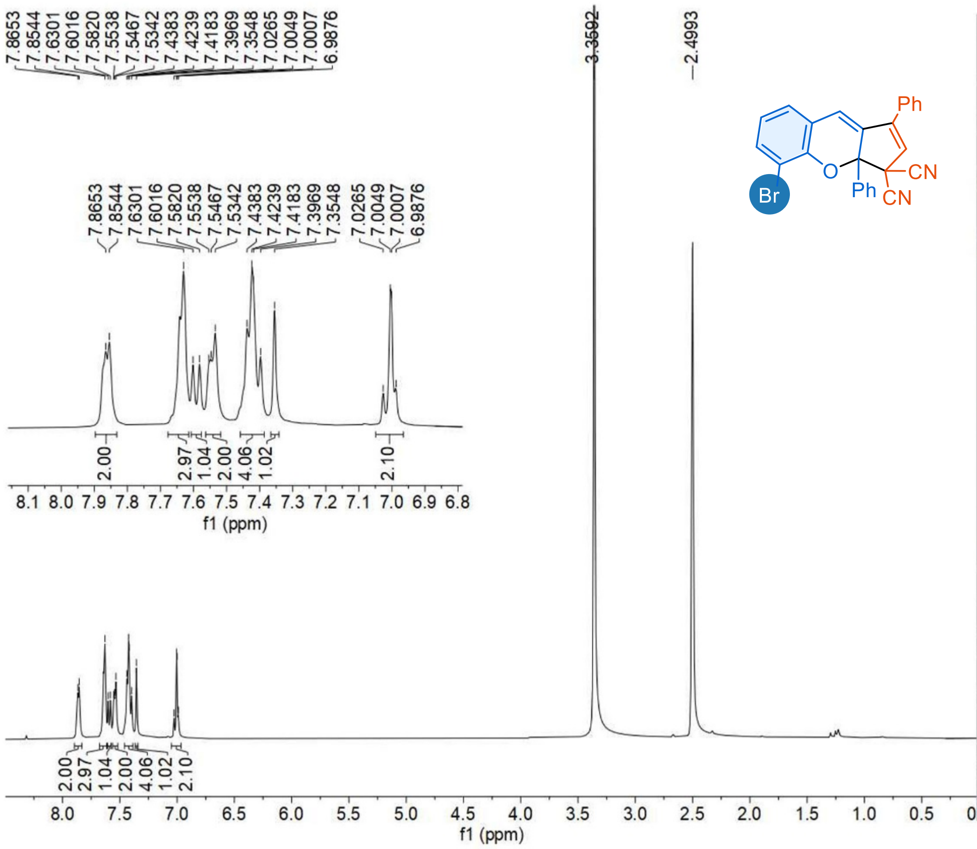

Parameter	值
1 Data File Name	23-1-hxw-H/ 82/ fid
2 标题	23-1-hxw-H. 82.fid
3 Comment	LAN
4 Origin	Bruker BioSpin GmbH
5 Owner	nmrsu
6 Site	
7 Instrument	Avance NEO 400
8 Author	
9 Solvent	DMSO
10 Temperature	295.0
11 Pulse Sequence	zg30
12 Experiment	1D
13 Probe	Z163739_0511 (PI HR-BBO400S1-BBF/ H/ D-5.0-Z SP)
14 Number of Scans	4
15 Receiver Gain	101.0
16 Relaxation Delay	1.0000
17 Pulse Width	8.0000
18 Presaturation Frequency	
19 Acquisition Time	3.9977
20 Acquisition Date	2023-03-23T21:36:37
21 Modification Date	2023-03-23T21:36:40
22 Class	
23 Spectrometer Frequency	400.15
24 Spectral Width	8196.7
25 Lowest Frequency	-1630.2
26 Nucleus	1H
27 Acquired Size	32768
28 Spectral Size	65536
29 Digital Resolution	0.13

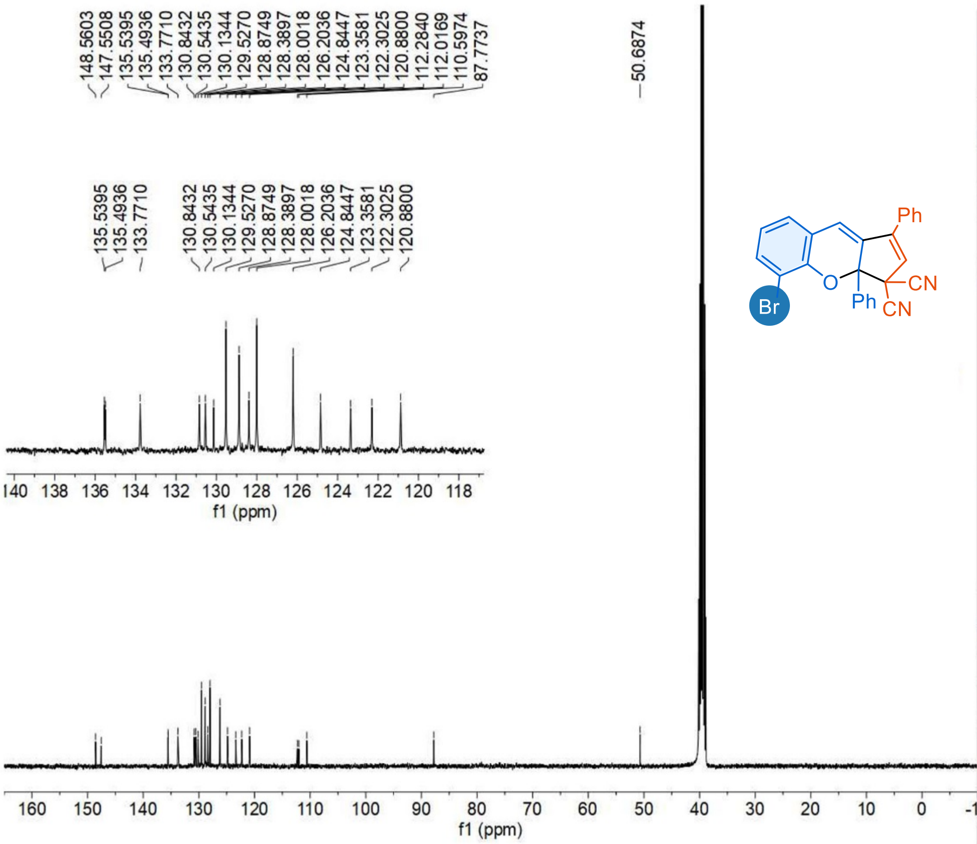

Parameter	值
1 Data File Name	2023 hxw0627/ 23-1-hxw-C/ 69/ fid
2 标题	23-1-hxw-C. 69.fid
3 Comment	LAN
4 Origin	Bruker BioSpin GmbH
5 Owner	nmrsu
6 Site	
7 Instrument	Avance NEO 400
8 Author	
9 Solvent	DMSO
10 Temperature	295.6
11 Pulse Sequence	zgpg30
12 Experiment	1D
13 Probe	Z163739_0511 (PI HR-BBO400S1-BBF/ H/ D-5.0-Z SP)
14 Number of Scans	1400
15 Receiver Gain	16.3
16 Relaxation Delay	2.0000
17 Pulse Width	8.0000
18 Presaturation Frequency	
19 Acquisition Time	1.3763
20 Acquisition Date	2023-03-24T01:07:45
21 Modification Date	2023-03-24T01:07:52
22 Class	
23 Spectrometer Frequency	100.63
24 Spectral Width	23809.5
25 Lowest Frequency	-1888.0
26 Nucleus	13C
27 Acquired Size	32768
28 Spectral Size	65536
29 Digital Resolution	0.36

5-Chloro-1,3a-diphenylcyclopenta[*b*]chromene-3,3(3a*H*)-dicarbonitrile (product 3ha)

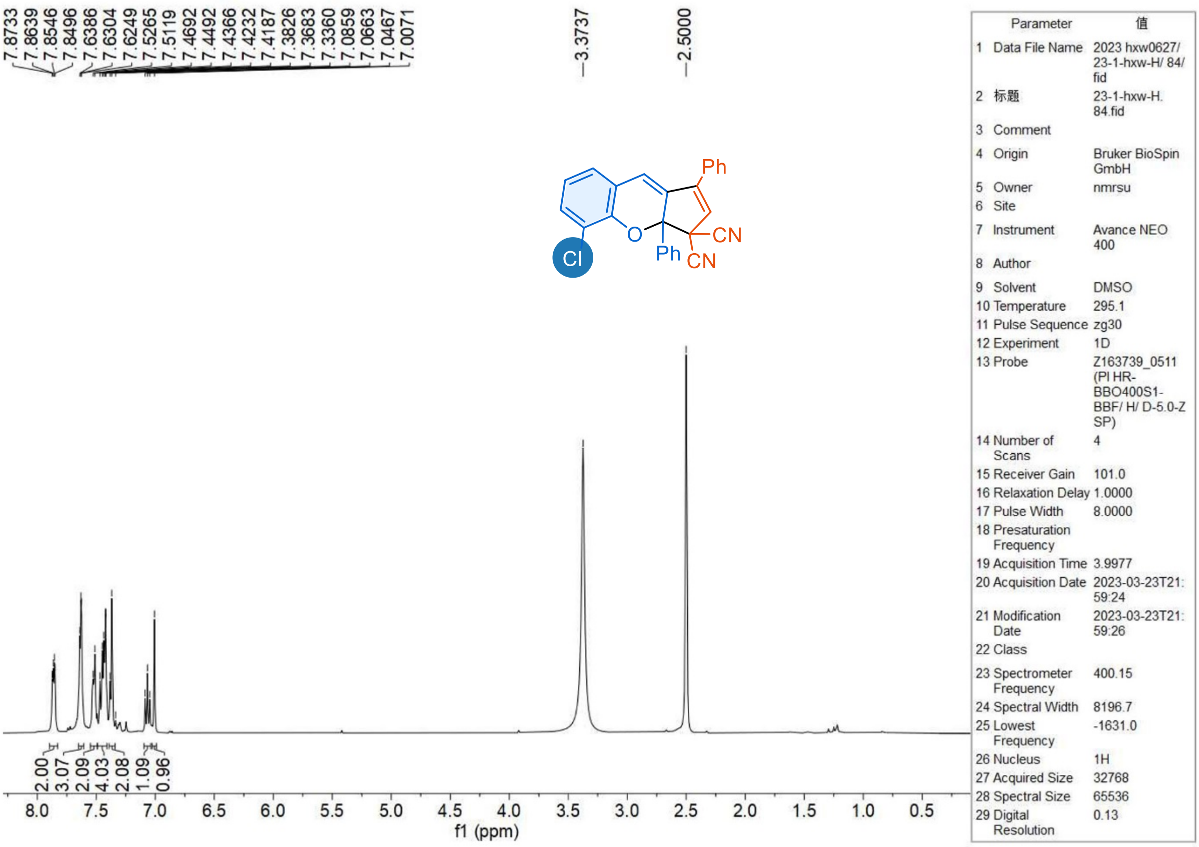

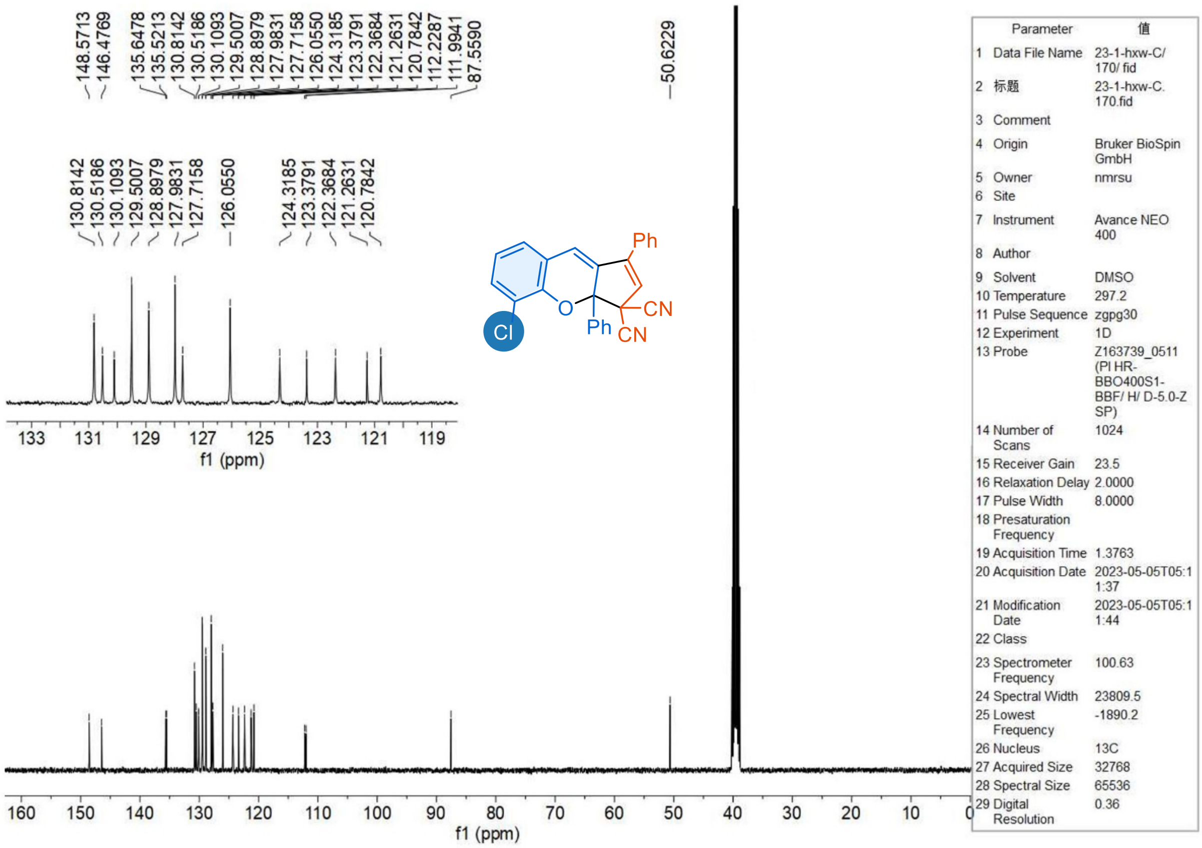

5-Methoxy-1,3a-diphenylcyclopenta[*b*]chromene-3,3(3a*H*)-dicarbonitrile (product 3ia)

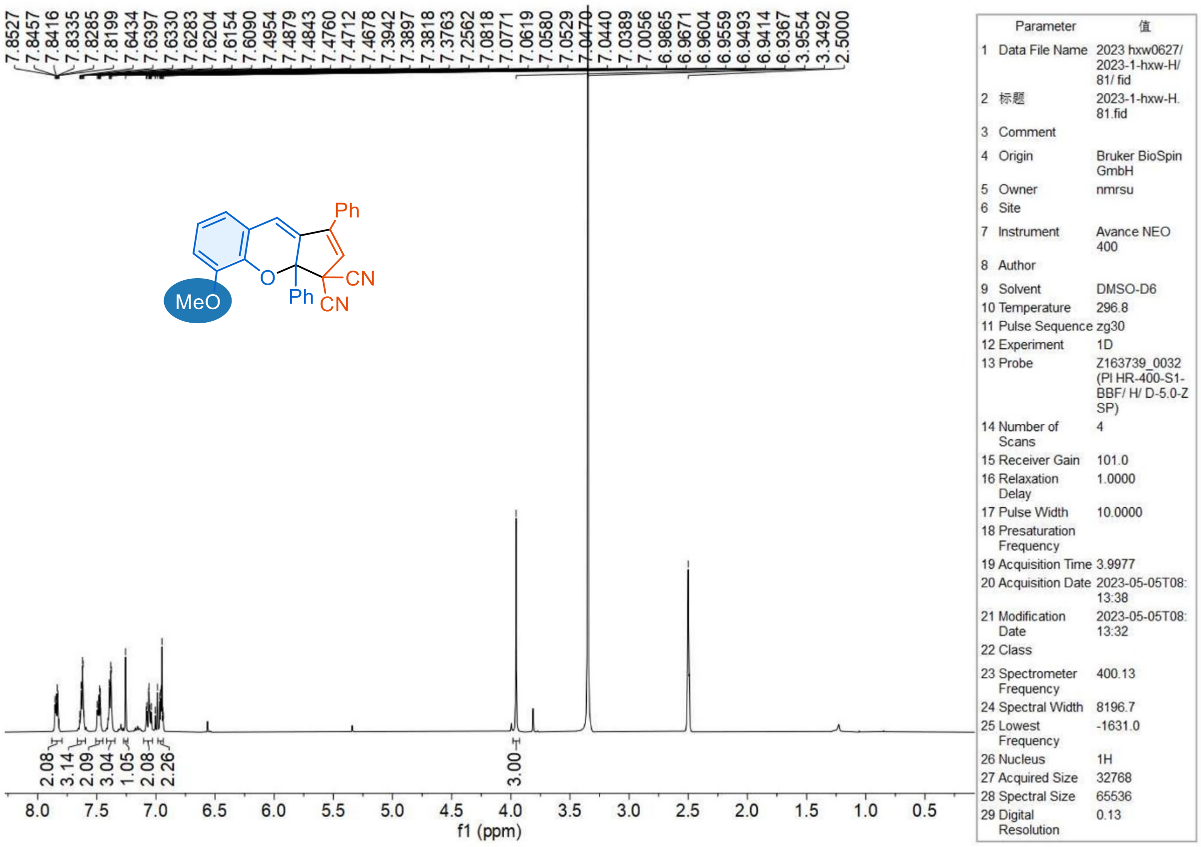

	Parameter	值
1	Data File Name	2023 hxw0627/ 2023-1-hxw-H/ 81/ fid
2	标题	2023-1-hxw-H. 81.fid
3	Comment	
4	Origin	Bruker BioSpin GmbH
5	Owner	nmrsu
6	Site	
7	Instrument	Avance NEO 400
8	Author	
9	Solvent	DMSO-D6
10	Temperature	296.8
11	Pulse Sequence	zg30
12	Experiment	1D
13	Probe	Z163739_0032 (PI HR-400-S1-BBF/ H/ D-5.0-Z SP)
14	Number of Scans	4
15	Receiver Gain	101.0
16	Relaxation Delay	1.0000
17	Pulse Width	10.0000
18	Presaturation Frequency	
19	Acquisition Time	3.9977
20	Acquisition Date	2023-05-05T08:13:38
21	Modification Date	2023-05-05T08:13:32
22	Class	
23	Spectrometer Frequency	400.13
24	Spectral Width	8196.7
25	Lowest Frequency	-1631.0
26	Nucleus	1H
27	Acquired Size	32768
28	Spectral Size	65536
29	Digital Resolution	0.13

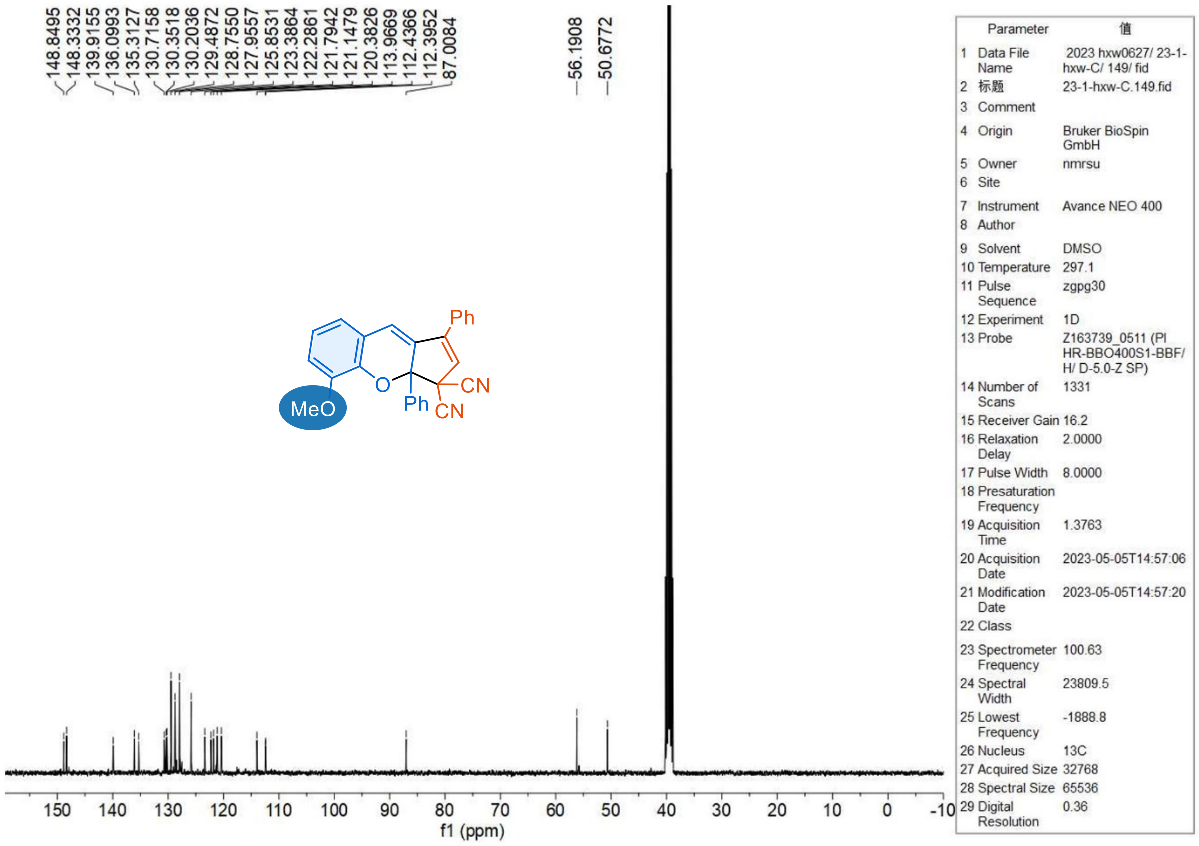

	Parameter	值
1	Data File Name	2023 hxw0627/ 23-1-hxw-C/ 149/ fid
2	标题	23-1-hxw-C.149.fid
3	Comment	
4	Origin	Bruker BioSpin GmbH
5	Owner	nmrsu
6	Site	
7	Instrument	Avance NEO 400
8	Author	
9	Solvent	DMSO
10	Temperature	297.1
11	Pulse Sequence	zgpg30
12	Experiment	1D
13	Probe	Z163739_0511 (PI HR-BBO400S1-BBF/ H/ D-5.0-Z SP)
14	Number of Scans	1331
15	Receiver Gain	16.2
16	Relaxation Delay	2.0000
17	Pulse Width	8.0000
18	Presaturation Frequency	
19	Acquisition Time	1.3763
20	Acquisition Date	2023-05-05T14:57:06
21	Modification Date	2023-05-05T14:57:20
22	Class	
23	Spectrometer Frequency	100.63
24	Spectral Width	23809.5
25	Lowest Frequency	-1888.8
26	Nucleus	13C
27	Acquired Size	32768
28	Spectral Size	65536
29	Digital Resolution	0.36

5,7-Dibromo-1,3a-diphenylcyclopenta[*b*]chromene-3,3(3a*H*)-dicarbonitrile (product 3ja)

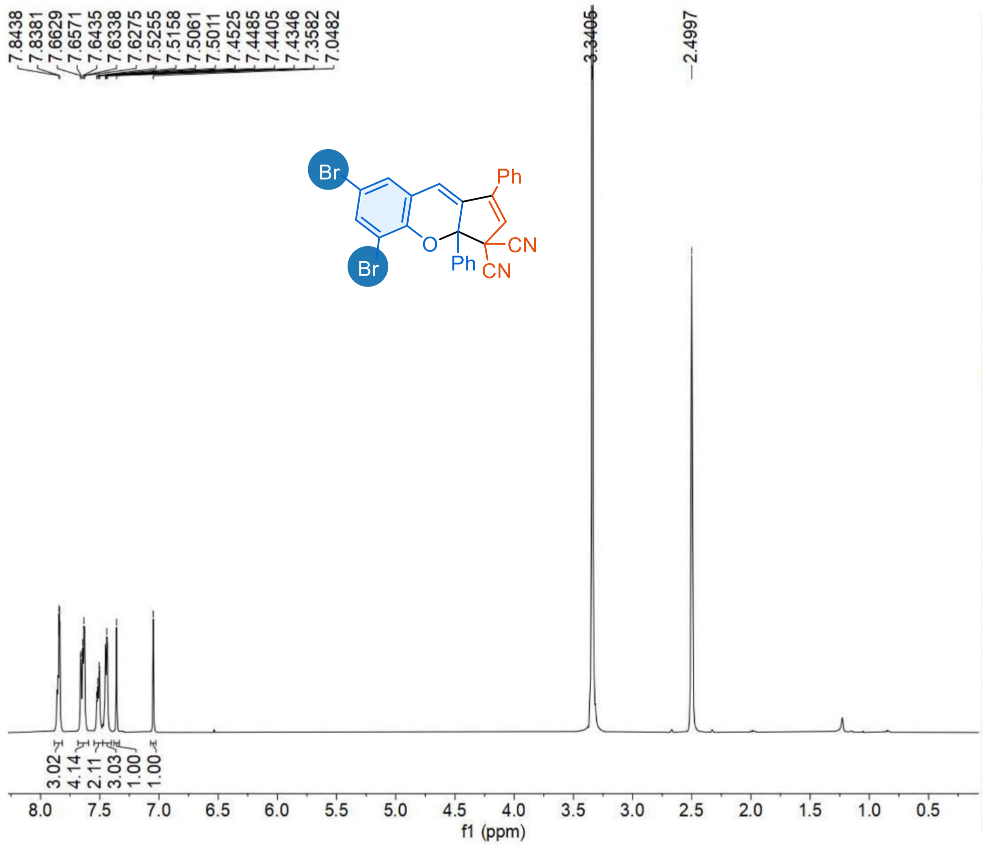

	Parameter	值
1	Data File Name	23-1-hxw-H/ 177/ fid
2	标题	23-1-hxw-H. 177.fid
3	Comment	LYN-ZI
4	Origin	Bruker BioSpin GmbH
5	Owner	nmrsu
6	Site	
7	Instrument	Avance NEO 400
8	Author	
9	Solvent	DMSO
10	Temperature	296.8
11	Pulse Sequence	zg30
12	Experiment	1D
13	Probe	Z163739_0511 (PI HR-BBO400S1-BBF/ H/ D-5.0-Z SP)
14	Number of Scans	4
15	Receiver Gain	101.0
16	Relaxation Delay	1.0000
17	Pulse Width	8.0000
18	Presaturation Frequency	
19	Acquisition Time	3.9977
20	Acquisition Date	2023-05-16T21:02:37
21	Modification Date	2023-05-16T21:02:44
22	Class	
23	Spectrometer Frequency	400.15
24	Spectral Width	8196.7
25	Lowest Frequency	-1630.5
26	Nucleus	1H
27	Acquired Size	32768
28	Spectral Size	65536
29	Digital Resolution	0.13

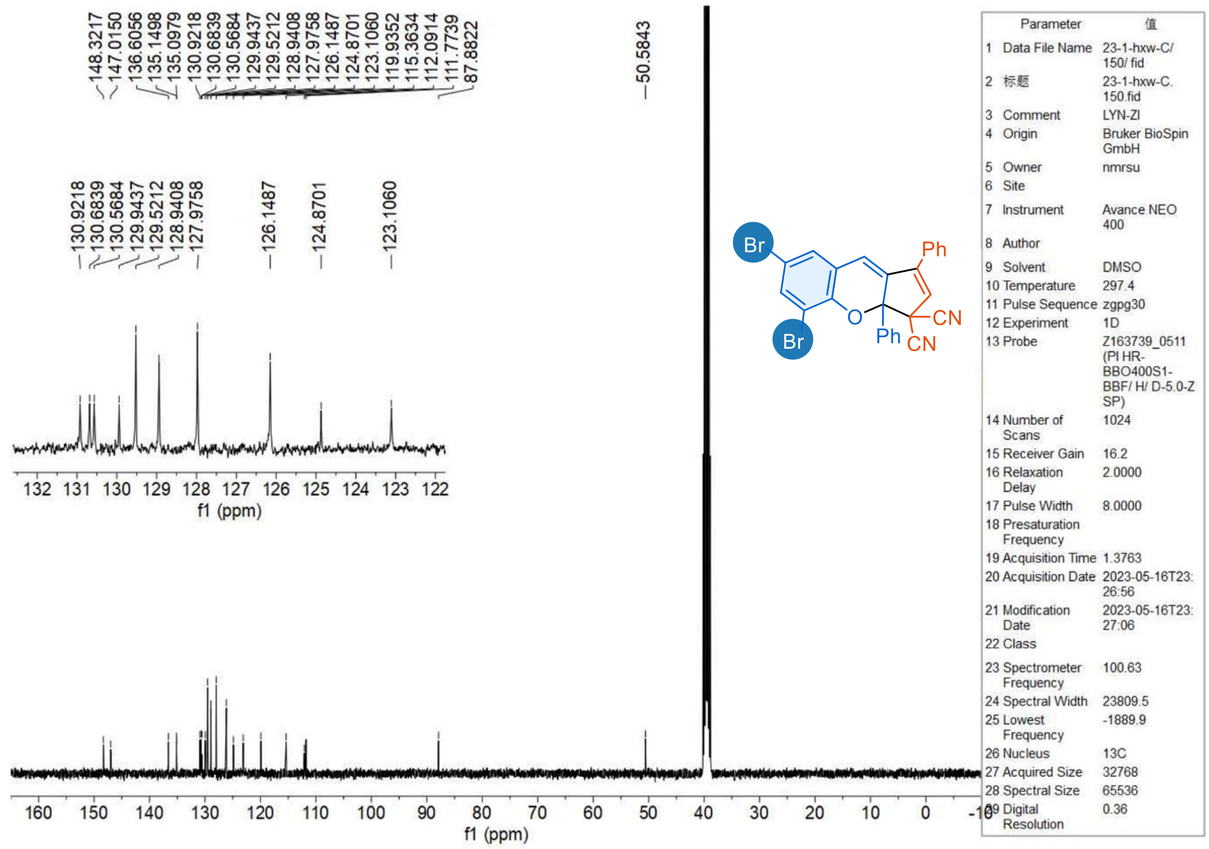

	Parameter	值
1	Data File Name	23-1-hxw-C/ 150/ fid
2	标题	23-1-hxw-C. 150.fid
3	Comment	LYN-ZI
4	Origin	Bruker BioSpin GmbH
5	Owner	nmrsu
6	Site	
7	Instrument	Avance NEO 400
8	Author	
9	Solvent	DMSO
10	Temperature	297.4
11	Pulse Sequence	zgpg30
12	Experiment	1D
13	Probe	Z163739_0511 (PI HR-BBO400S1-BBF/ H/ D-5.0-Z SP)
14	Number of Scans	1024
15	Receiver Gain	16.2
16	Relaxation Delay	2.0000
17	Pulse Width	8.0000
18	Presaturation Frequency	
19	Acquisition Time	1.3763
20	Acquisition Date	2023-05-16T23:26:56
21	Modification Date	2023-05-16T23:27:06
22	Class	
23	Spectrometer Frequency	100.63
24	Spectral Width	23809.5
25	Lowest Frequency	-1889.9
26	Nucleus	13C
27	Acquired Size	32768
28	Spectral Size	65536
29	Digital Resolution	0.36

6-Chloro-1,3a-diphenylcyclopenta[*b*]chromene-3,3(3a*H*)-dicarbonitrile (product 3ka)

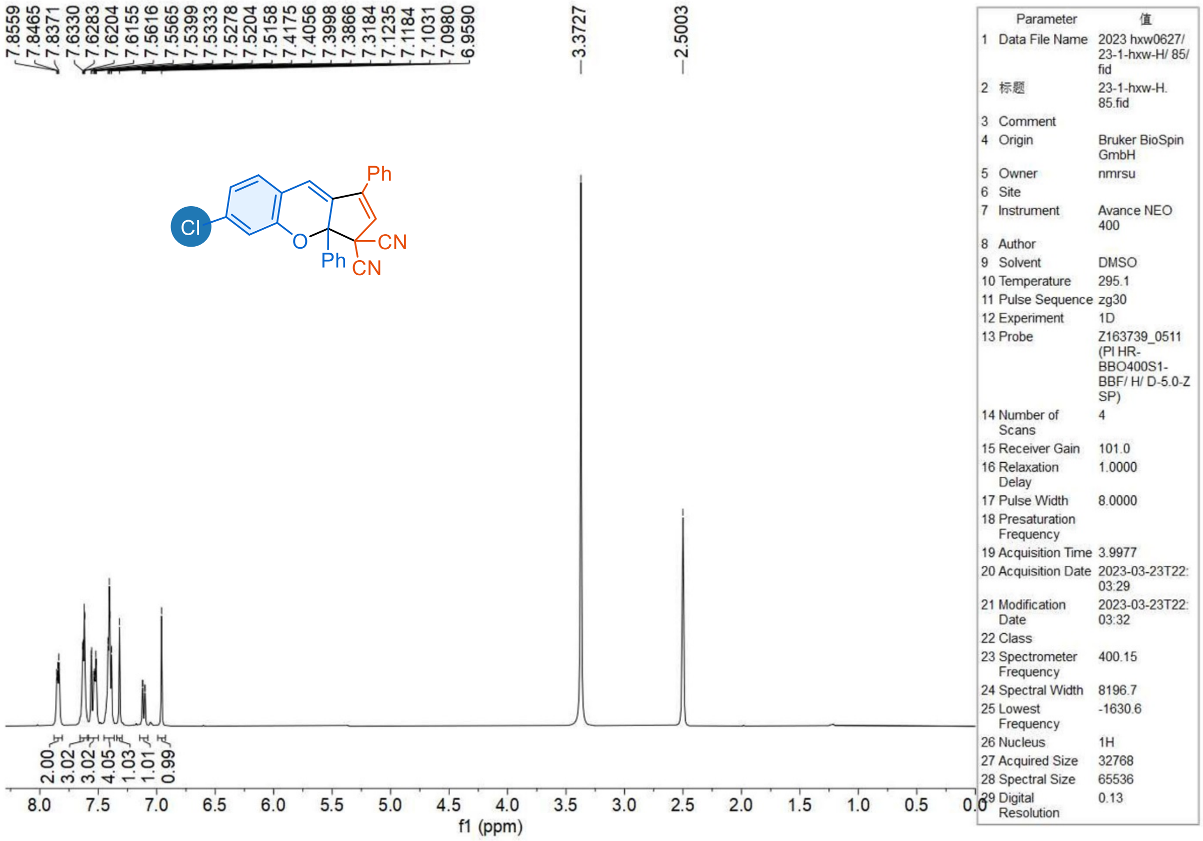

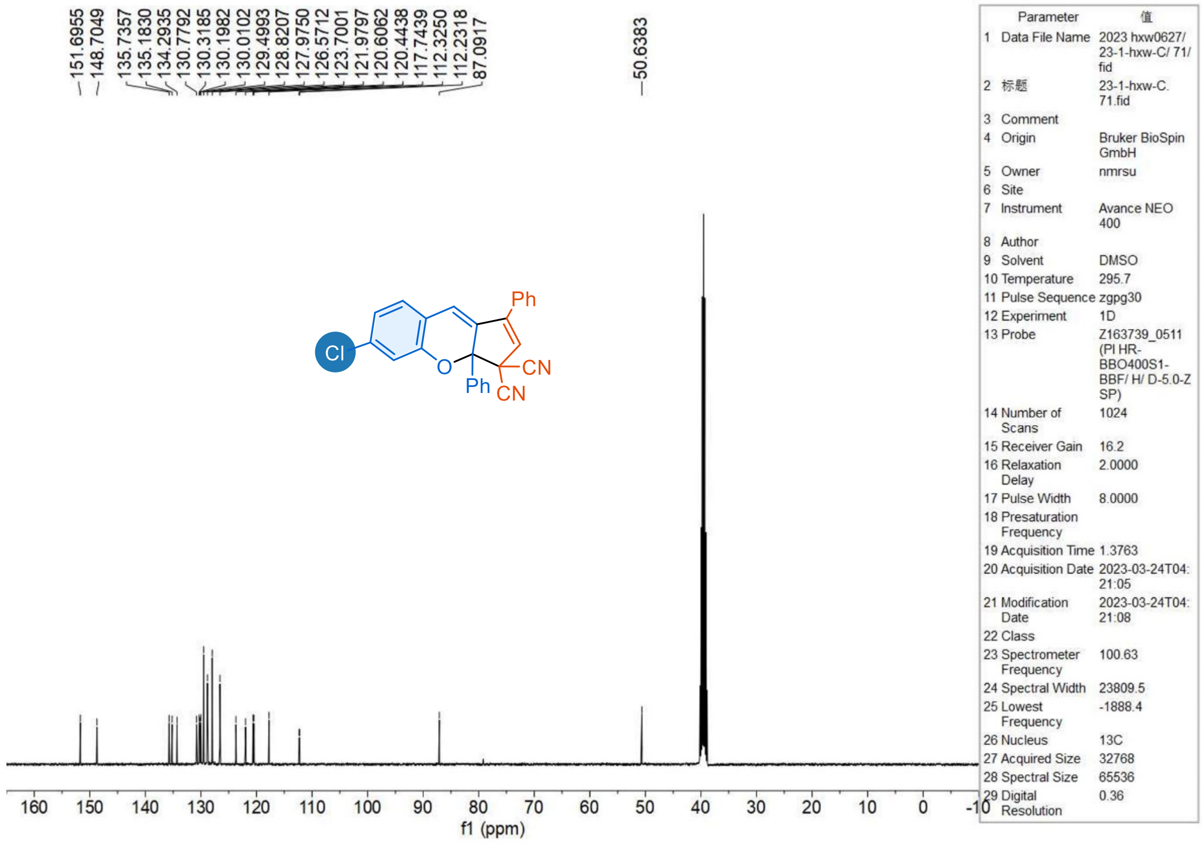

1-Phenyl-3a-(*m*-tolyl)cyclopenta[*b*]chromene-3,3(3a*H*)-dicarbonitrile (product 3la)

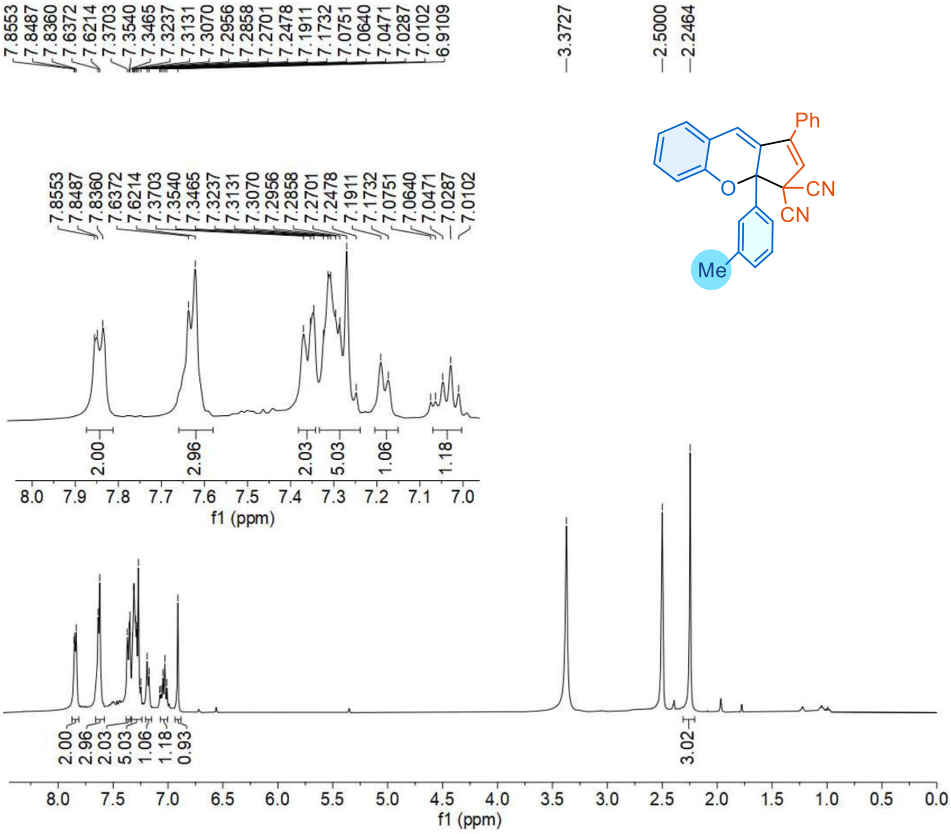

	Parameter	值
1	Data File Name	23-1-hxw-H/ 73/ fid
2	标题	23-1-hxw-H. 73.fid
3	Comment	lyn-hong
4	Origin	Bruker BioSpin GmbH
5	Owner	nmrsu
6	Site	
7	Instrument	Avance NEO 400
8	Author	
9	Solvent	DMSO
10	Temperature	295.0
11	Pulse Sequence	zg30
12	Experiment	1D
13	Probe	Z163739_0511 (PI HR-BBO400S1-BBF/ H/ D-5.0-Z SP)
14	Number of Scans	4
15	Receiver Gain	99.7
16	Relaxation Delay	1.0000
17	Pulse Width	8.0000
18	Presaturation Frequency	
19	Acquisition Time	3.9977
20	Acquisition Date	2023-03-22T21:33:34
21	Modification Date	2023-03-22T21:33:42
22	Class	
23	Spectrometer Frequency	400.15
24	Spectral Width	8196.7
25	Lowest Frequency	-1630.5
26	Nucleus	1H
27	Acquired Size	32768
28	Spectral Size	65536
29	Digital Resolution	0.13

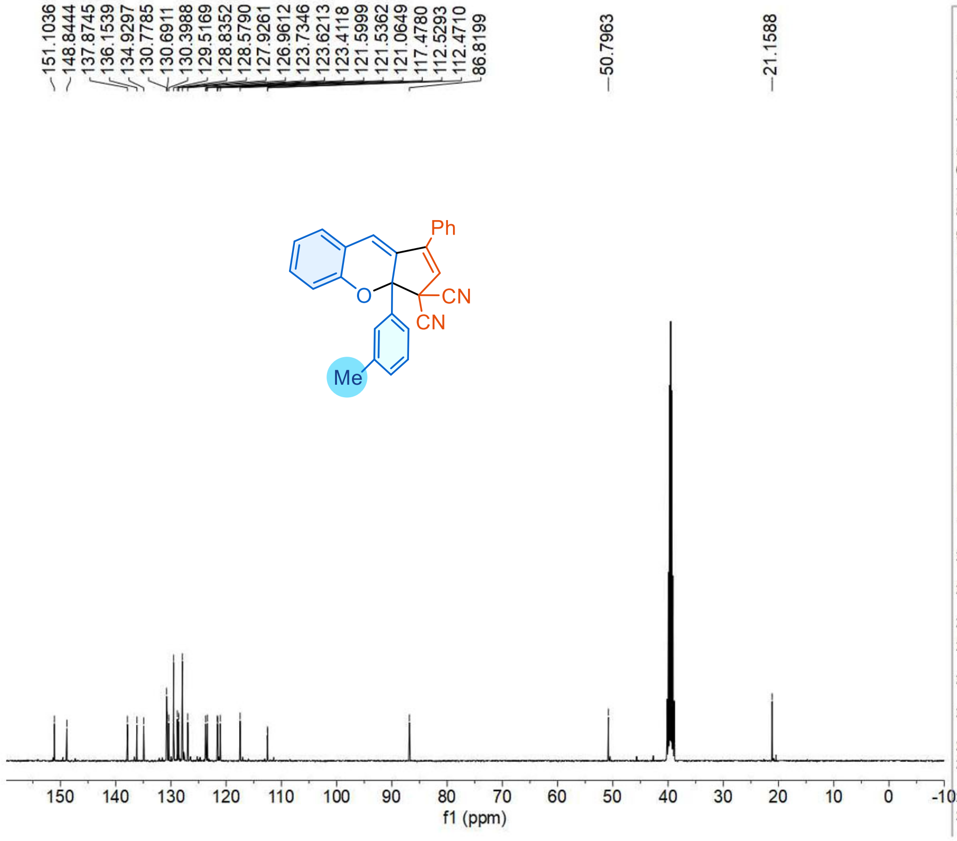

	Parameter	值
1	Data File Name	23-1-hxw-C/ 66/ fid
2	标题	23-1-hxw-C.66.fid
3	Comment	
4	Origin	Bruker BioSpin GmbH
5	Owner	nmrsu
6	Site	
7	Instrument	Avance NEO 400
8	Author	
9	Solvent	DMSO
10	Temperature	295.5
11	Pulse Sequence	zgpg30
12	Experiment	1D
13	Probe	Z163739_0511 (PI HR-BBO400S1-BBF/ H/ D-5.0-Z SP)
14	Number of Scans	1800
15	Receiver Gain	16.2
16	Relaxation Delay	2.0000
17	Pulse Width	8.0000
18	Presaturation Frequency	
19	Acquisition Time	1.3763
20	Acquisition Date	2023-03-23T03:59:02
21	Modification Date	2023-03-23T03:59:04
22	Class	
23	Spectrometer Frequency	100.63
24	Spectral Width	23809.5
25	Lowest Frequency	-1888.8
26	Nucleus	13C
27	Acquired Size	32768
28	Spectral Size	65536
29	Digital Resolution	0.36

3a-(4-Chlorophenyl)-1-phenylcyclopenta[*b*]chromene-3,3(3a*H*)-dicarbonitrile (product 3ma)

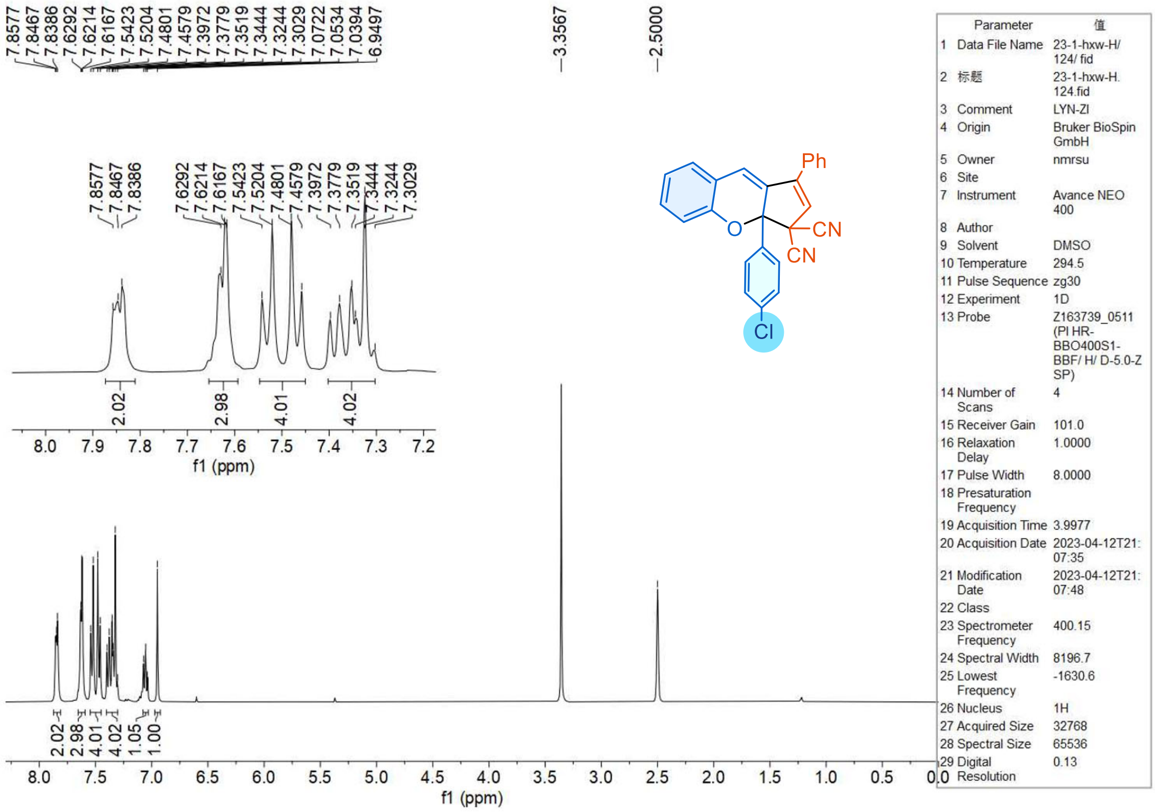

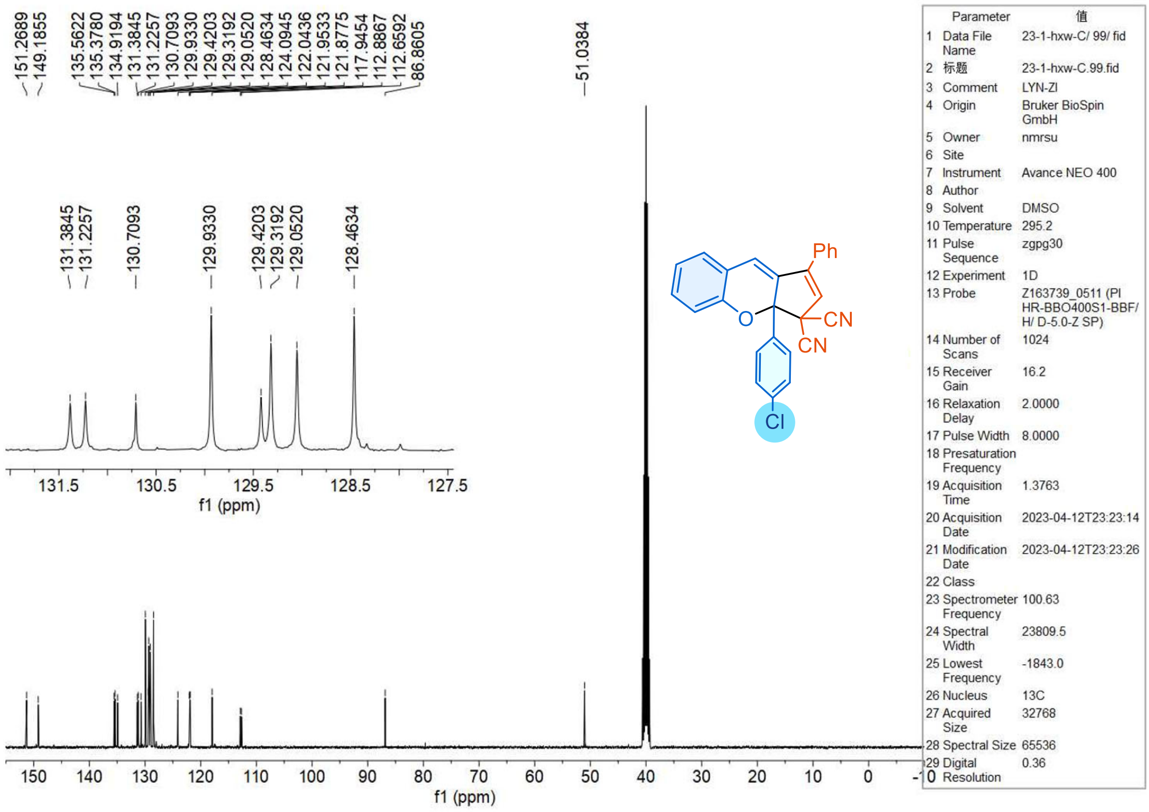

3a-(4-Bromophenyl)-1-phenylcyclopenta[*b*]chromene-3,3(3a*H*)-dicarbonitrile (product 3na)

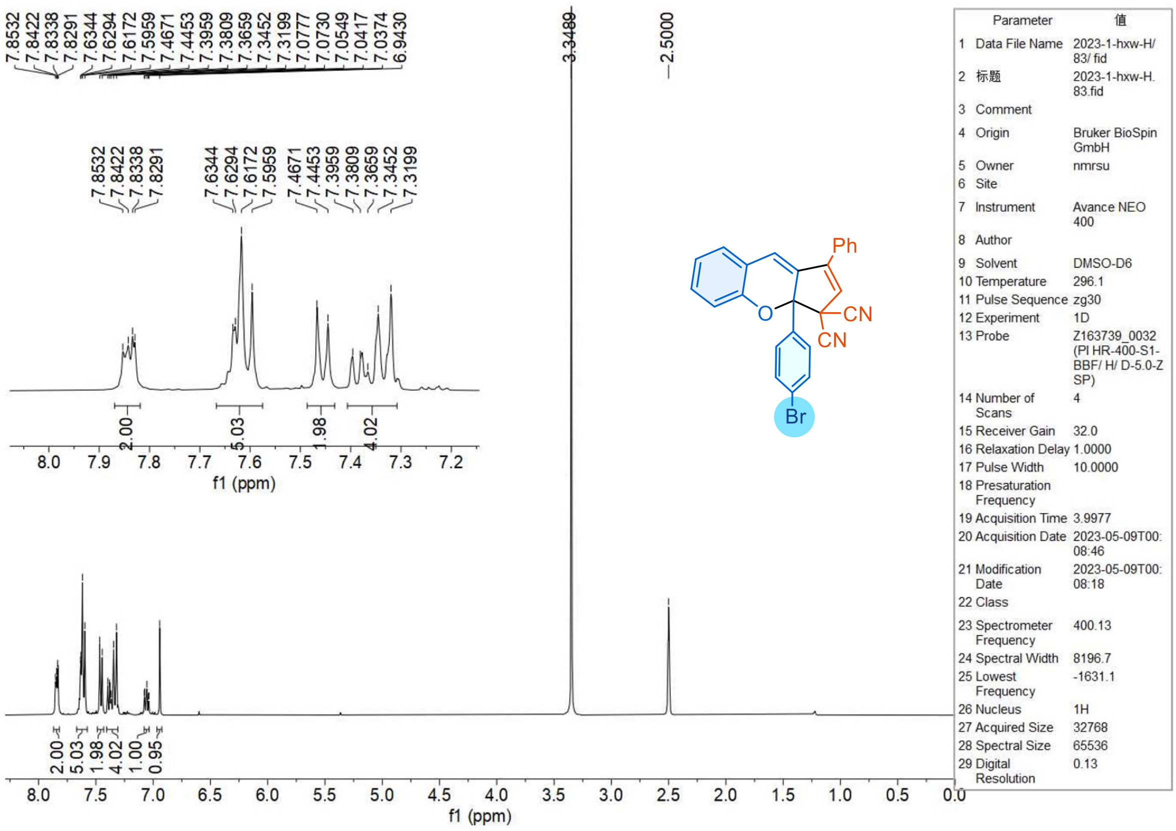

Parameter	值
1 Data File Name	2023-1-hxw-H/ 83/ fid
2 标题	2023-1-hxw-H. 83.fid
3 Comment	
4 Origin	Bruker BioSpin GmbH
5 Owner	nmrsu
6 Site	
7 Instrument	Avance NEO 400
8 Author	
9 Solvent	DMSO-D6
10 Temperature	296.1
11 Pulse Sequence	zg30
12 Experiment	1D
13 Probe	Z163739_0032 (PI HR-400-S1-BBF/ H/ D-5.0-Z SP)
14 Number of Scans	4
15 Receiver Gain	32.0
16 Relaxation Delay	1.0000
17 Pulse Width	10.0000
18 Presaturation Frequency	
19 Acquisition Time	3.9977
20 Acquisition Date	2023-05-09T00:08:46
21 Modification Date	2023-05-09T00:08:18
22 Class	
23 Spectrometer Frequency	400.13
24 Spectral Width	8196.7
25 Lowest Frequency	-1631.1
26 Nucleus	1H
27 Acquired Size	32768
28 Spectral Size	65536
29 Digital Resolution	0.13

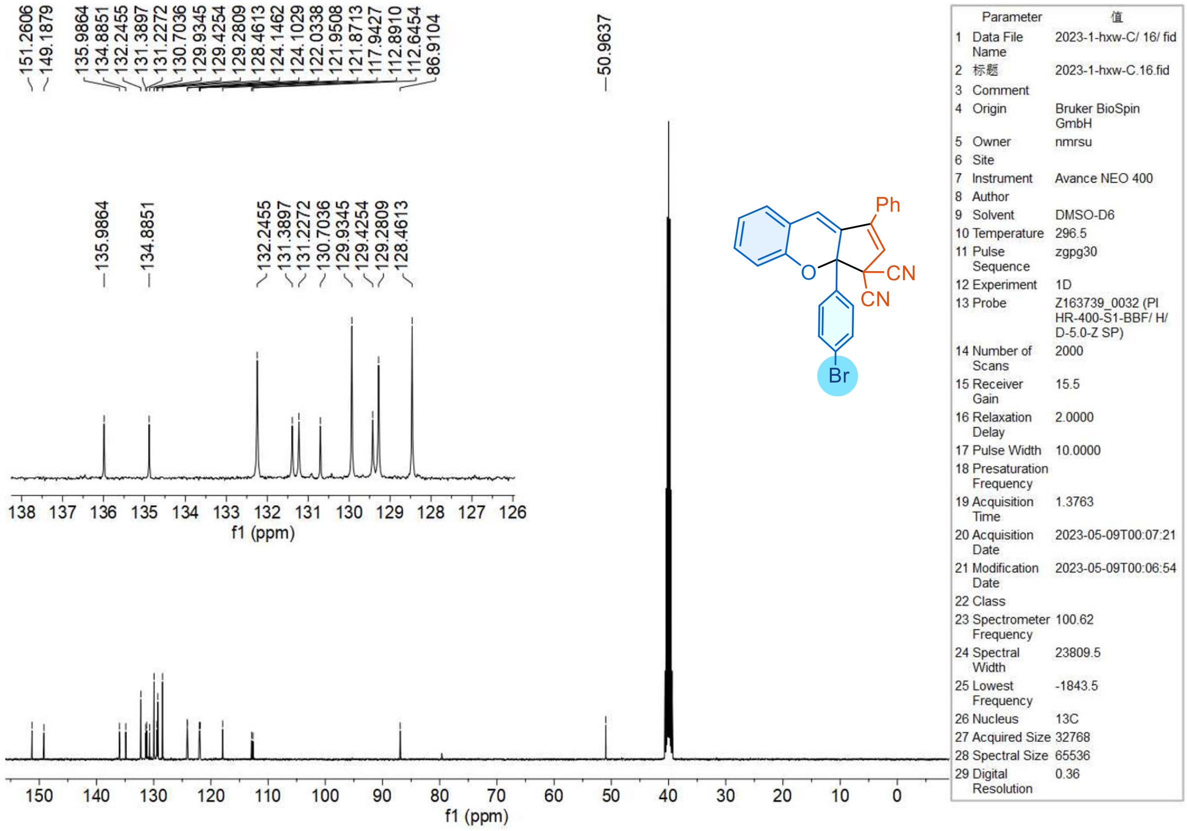

Parameter	值
1 Data File Name	2023-1-hxw-C/ 16/ fid
2 标题	2023-1-hxw-C.16.fid
3 Comment	
4 Origin	Bruker BioSpin GmbH
5 Owner	nmrsu
6 Site	
7 Instrument	Avance NEO 400
8 Author	
9 Solvent	DMSO-D6
10 Temperature	296.5
11 Pulse Sequence	zgpg30
12 Experiment	1D
13 Probe	Z163739_0032 (PI HR-400-S1-BBF/ H/ D-5.0-Z SP)
14 Number of Scans	2000
15 Receiver Gain	15.5
16 Relaxation Delay	2.0000
17 Pulse Width	10.0000
18 Presaturation Frequency	
19 Acquisition Time	1.3763
20 Acquisition Date	2023-05-09T00:07:21
21 Modification Date	2023-05-09T00:06:54
22 Class	
23 Spectrometer Frequency	100.62
24 Spectral Width	23809.5
25 Lowest Frequency	-1843.5
26 Nucleus	13C
27 Acquired Size	32768
28 Spectral Size	65536
29 Digital Resolution	0.36

3a-(4-Fluorophenyl)-1-phenylcyclopenta[*b*]chromene-3,3(3a*H*)-dicarbonitrile (product 3oa)

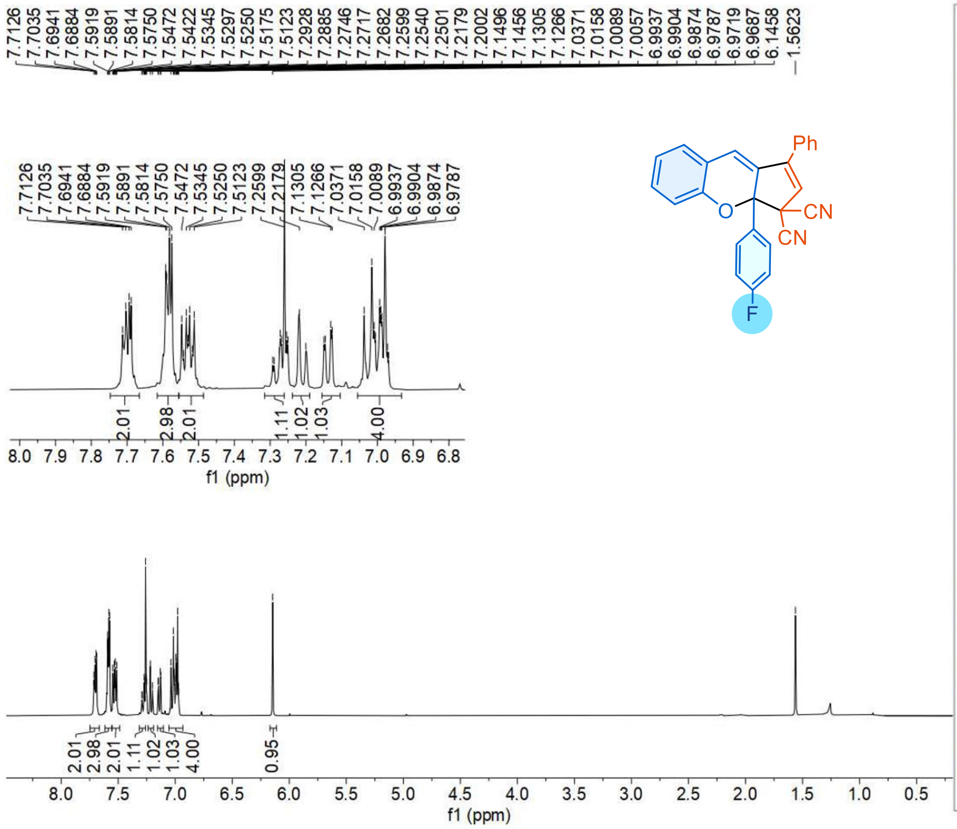

Parameter	值
1 Data File Name	2023hxw old 400/ 2023hxw old 400/ 2023-1-hxw-H/ 8/ fid
2 标题	2023-1-hxw-H.8.fid
3 Comment	
4 Origin	Bruker BioSpin GmbH
5 Owner	nmrsu
6 Site	
7 Instrument	Avance NEO 400
8 Author	
9 Solvent	CDCl3
10 Temperature	293.8
11 Pulse Sequence	zg30
12 Experiment	1D
13 Probe	Z163739_0032 (PI HR-400-S1-BBF/ H/ D-5.0-Z SP)
14 Number of Scans	4
15 Receiver Gain	101.0
16 Relaxation Delay	1.0000
17 Pulse Width	10.0000
18 Presaturation Frequency	
19 Acquisition Time	3.9977
20 Acquisition Date	2023-02-16T10:58:03
21 Modification Date	2023-02-16T10:57:48
22 Class	
23 Spectrometer Frequency	400.13
24 Spectral Width	8196.7
25 Lowest Frequency	-1637.3
26 Nucleus	1H
27 Acquired Size	32768
28 Spectral Size	65536
29 Digital Resolution	0.13

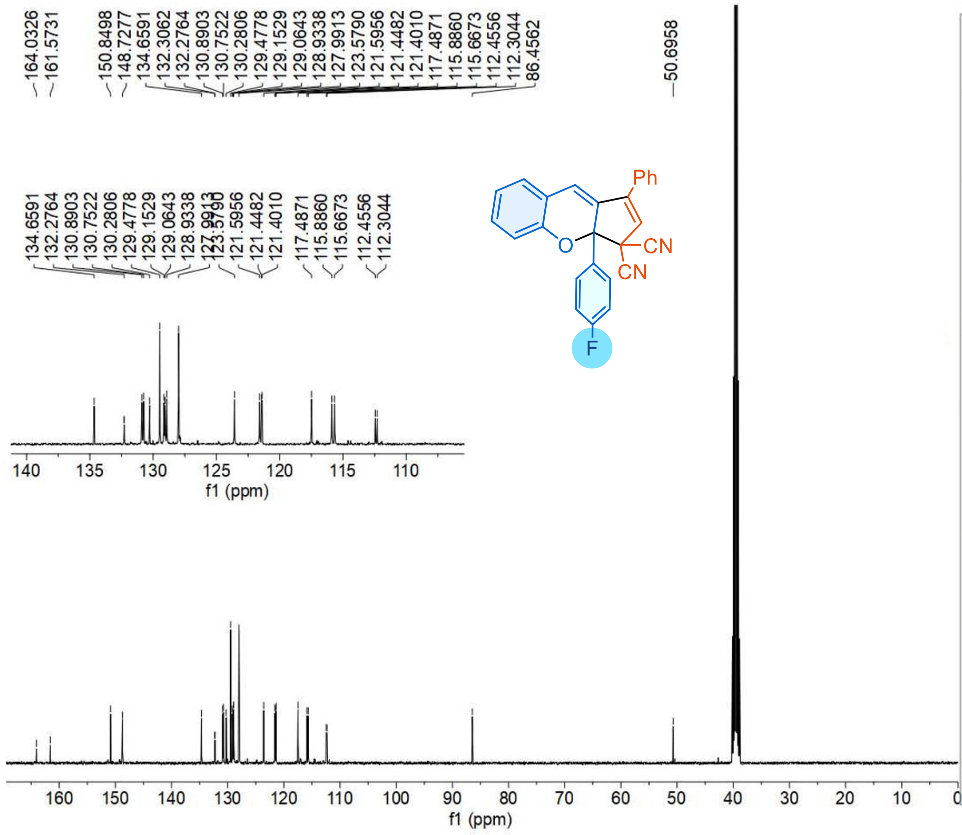

Parameter	值
1 Data File Name	23hxw-new 400/ 23-1-hxw-C/ 109/ fid
2 标题	23-1-hxw-C. 109.fid
3 Comment	
4 Origin	Bruker BioSpin GmbH
5 Owner	nmrsu
6 Site	
7 Instrument	Avance NEO 400
8 Author	
9 Solvent	DMSO
10 Temperature	295.6
11 Pulse Sequence	zgpg30
12 Experiment	1D
13 Probe	Z163739_0511 (PI HR-BBO400S1-BBF/ H/ D-5.0-Z SP)
14 Number of Scans	2000
15 Receiver Gain	16.2
16 Relaxation Delay	2.0000
17 Pulse Width	8.0000
18 Presaturation Frequency	
19 Acquisition Time	1.3763
20 Acquisition Date	2023-04-06T02:46:18
21 Modification Date	2023-04-06T02:46:24
22 Class	
23 Spectrometer Frequency	100.63
24 Spectral Width	23809.5
25 Lowest Frequency	-1888.8
26 Nucleus	13C
27 Acquired Size	32768
28 Spectral Size	65536
29 Digital Resolution	0.36

3a-(4-Fluorophenyl)-1-phenylcyclopenta[*b*]chromene-3,3(3a*H*)-dicarbonitrile (product 3oa)

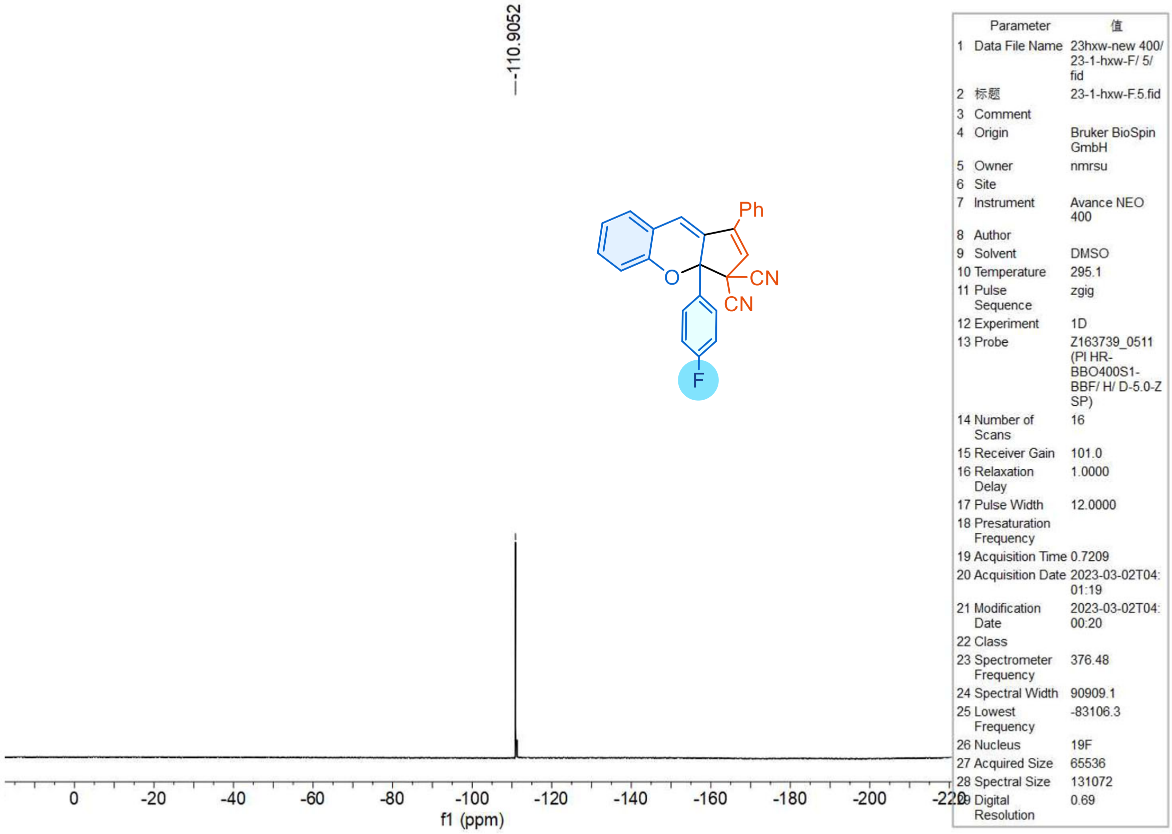

1-Phenyl-3a-(*p*-tolyl)cyclopenta[*b*]chromene-3,3(3a*H*)-dicarbonitrile (product 3pa)

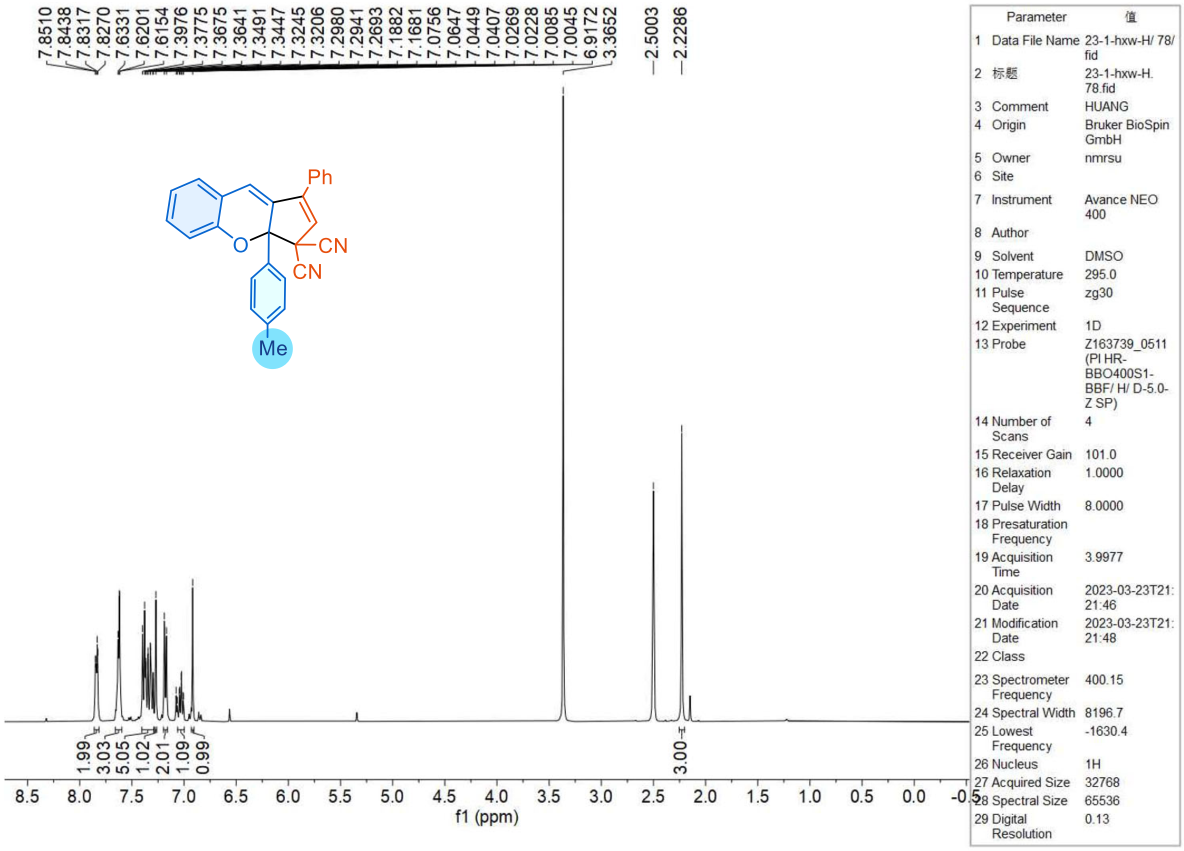

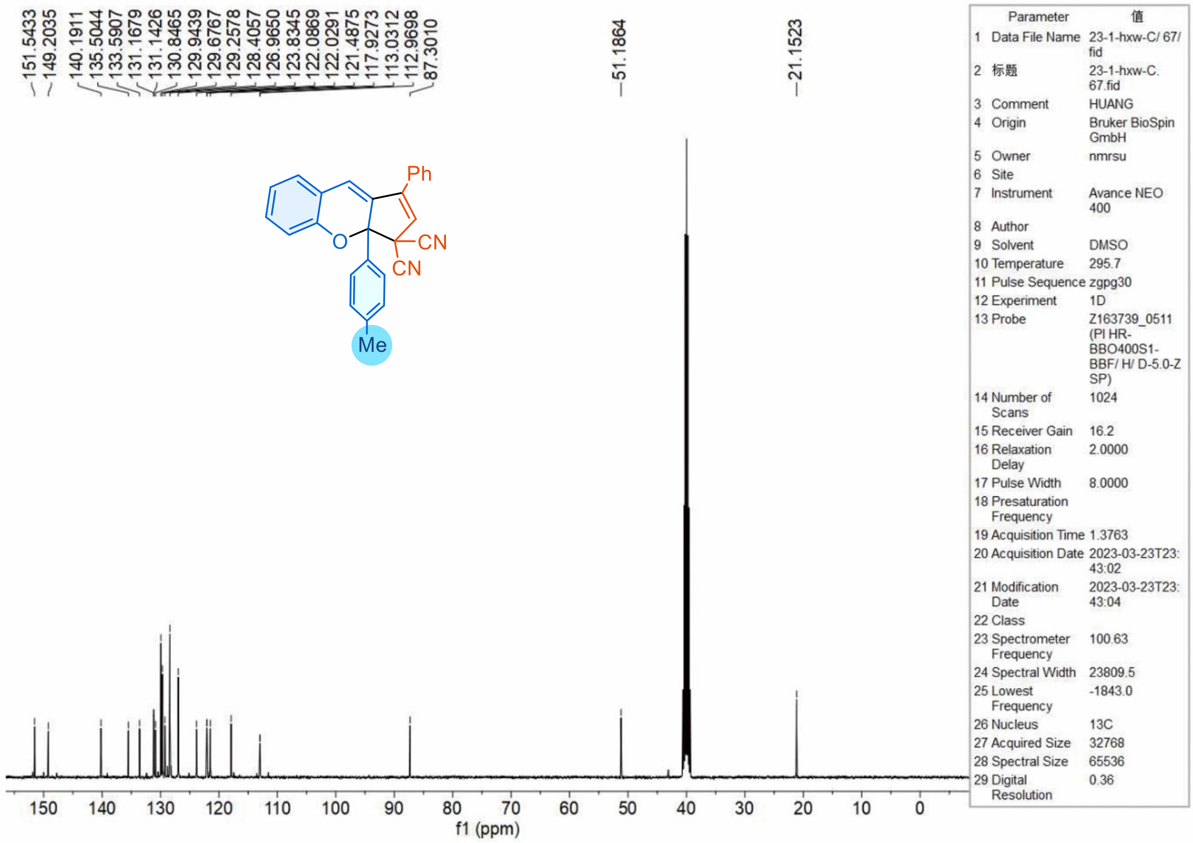

3a-([1,1'-Biphenyl]-4-yl)-1-phenylcyclopenta[*b*]chromene-3,3(3a*H*)-dicarbonitrile (product 3qa)

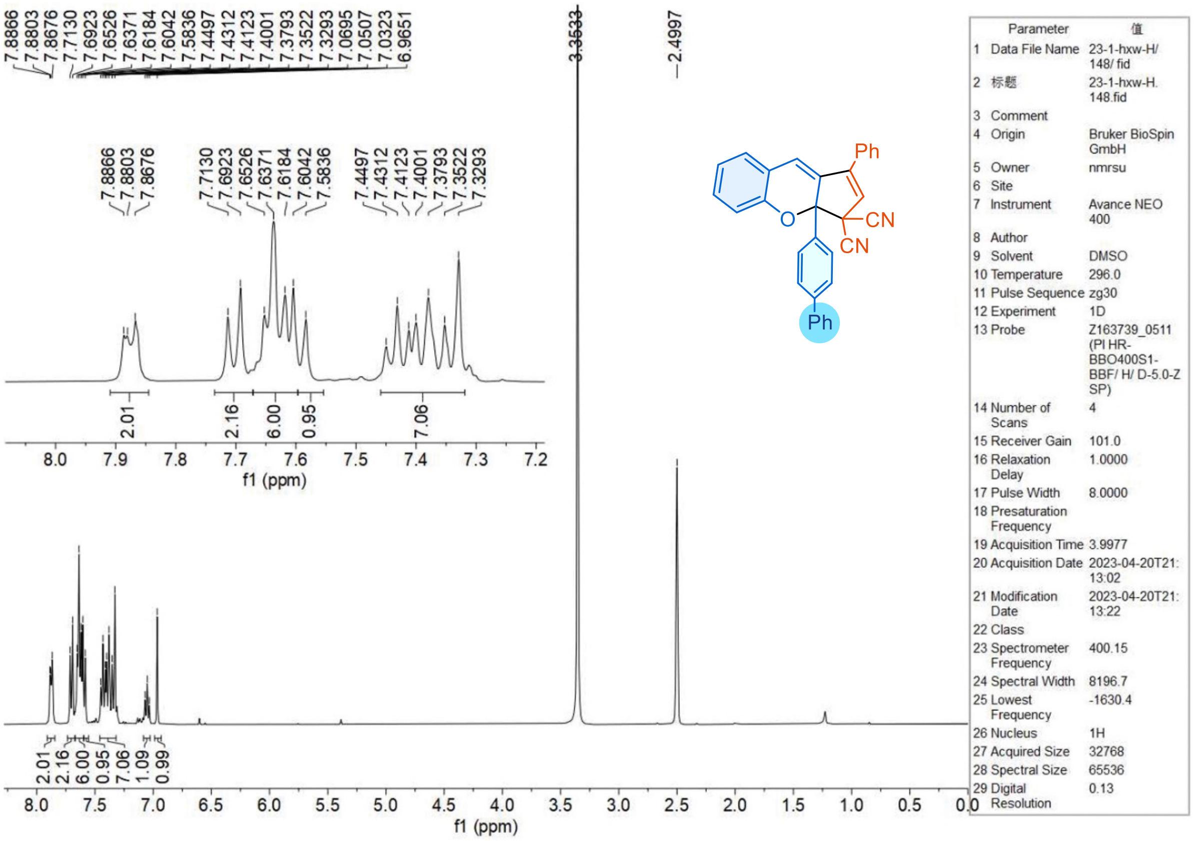

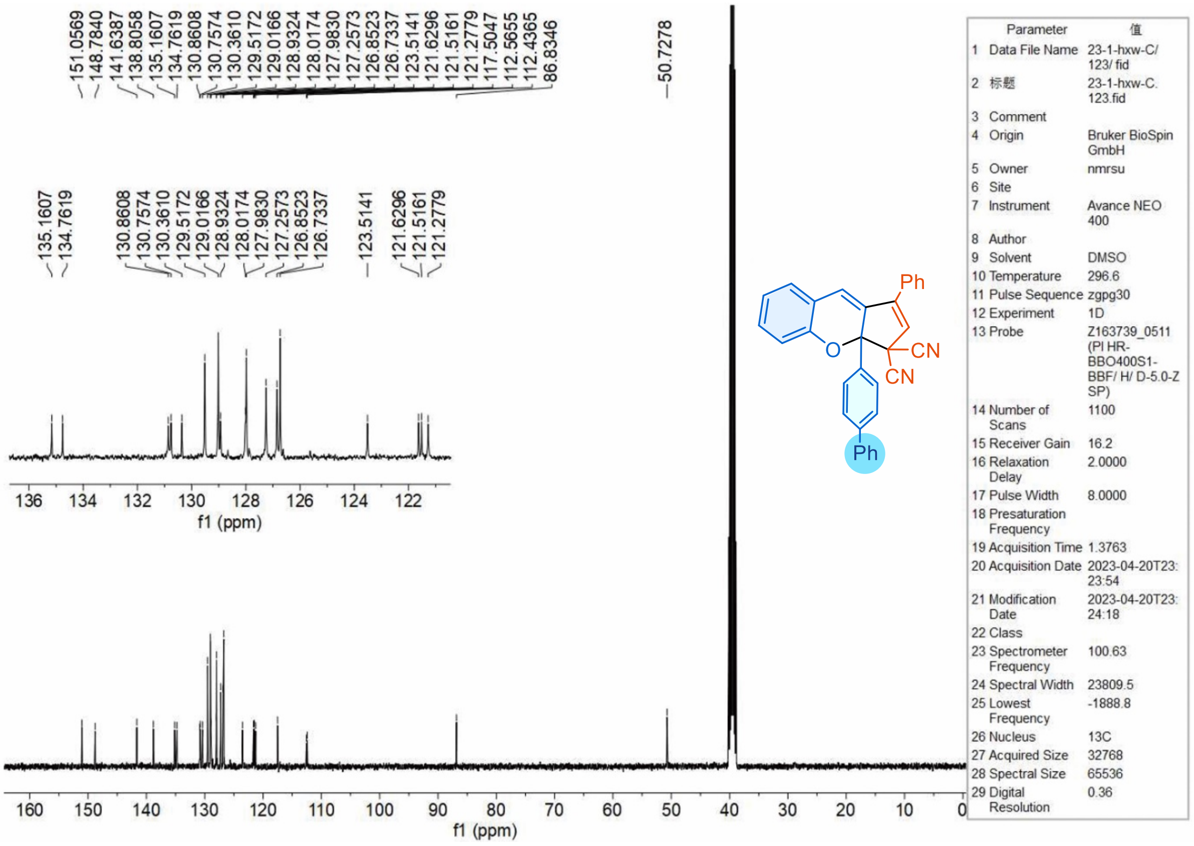

1-Phenyl-3a-(4'-propyl-[1,1'-biphenyl]-4-yl)cyclopenta[*b*]chromene-3,3(3a*H*)-dicarbonitrile (product 3ra)

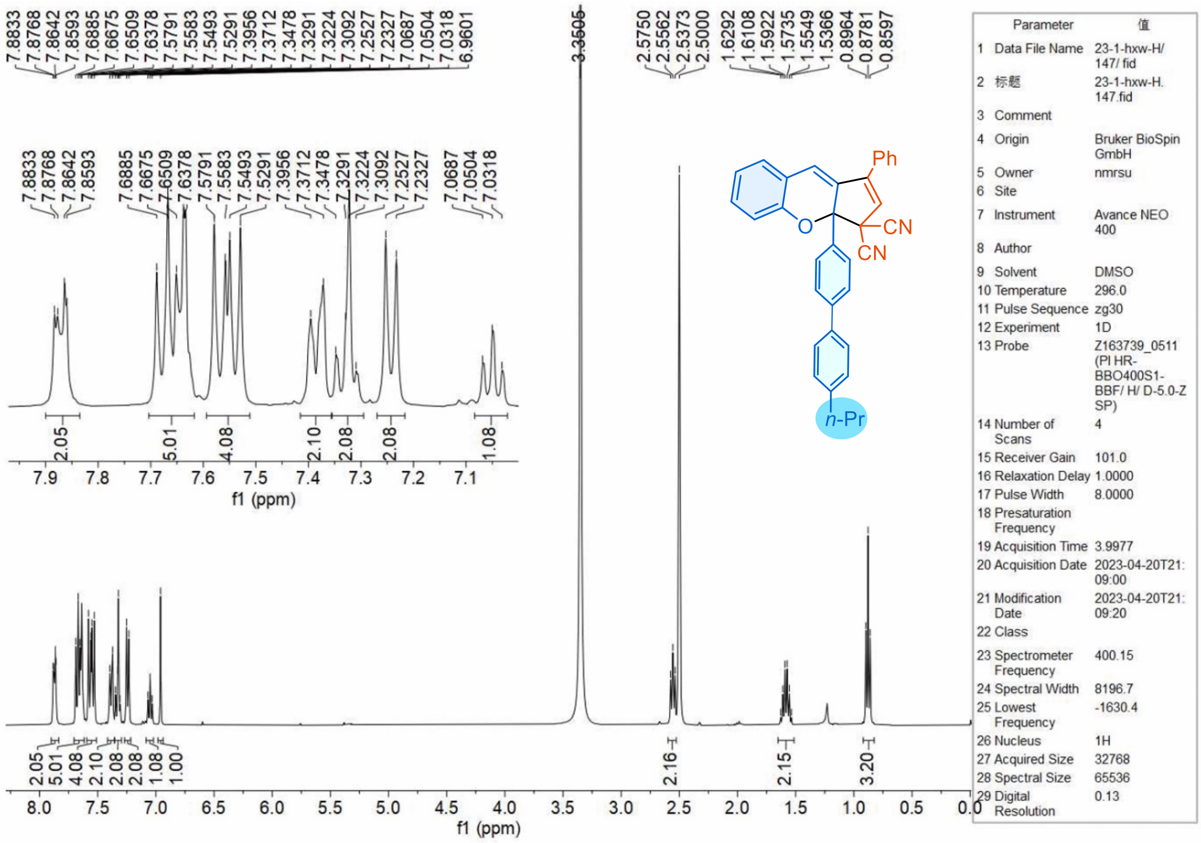

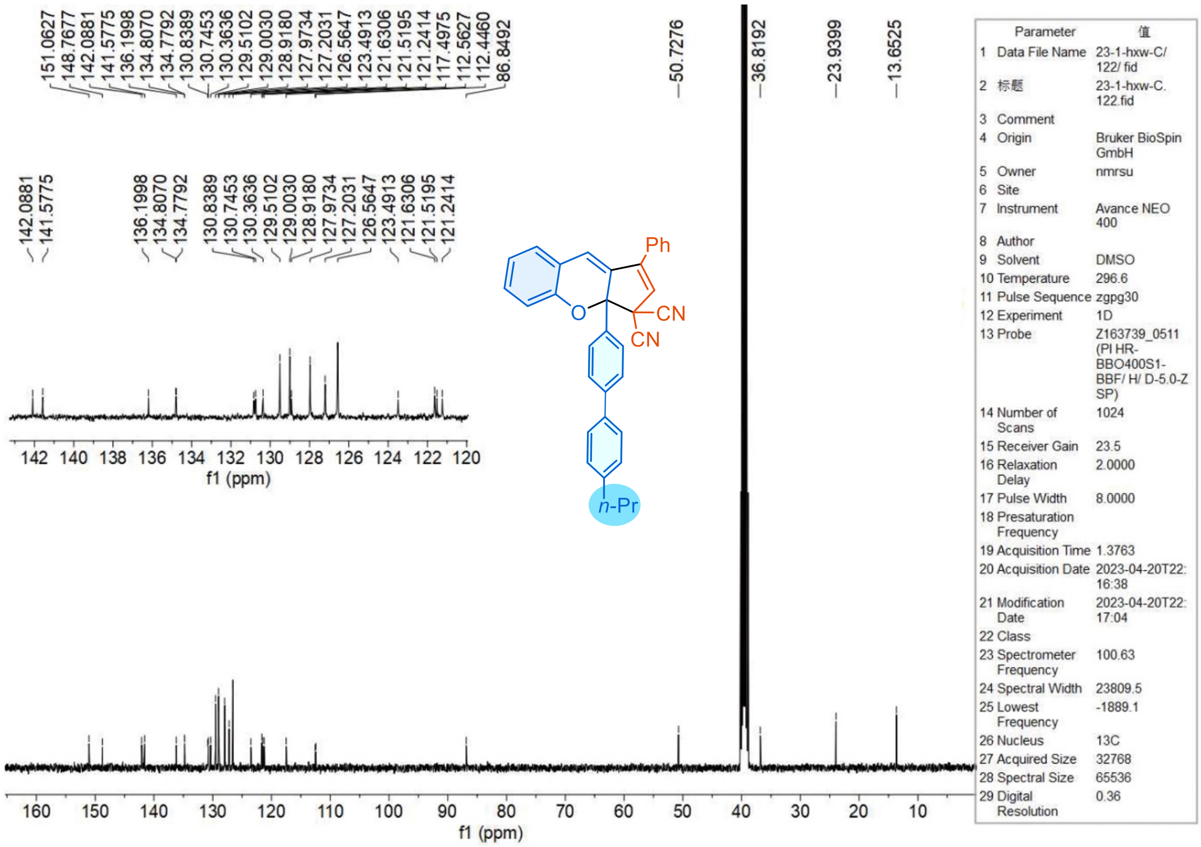

5-Methoxy-1-phenyl-3a-(*p*-tolyl)cyclopenta[*b*]chromene-3,3(3a*H*)-dicarbonitrile (product 3sa)

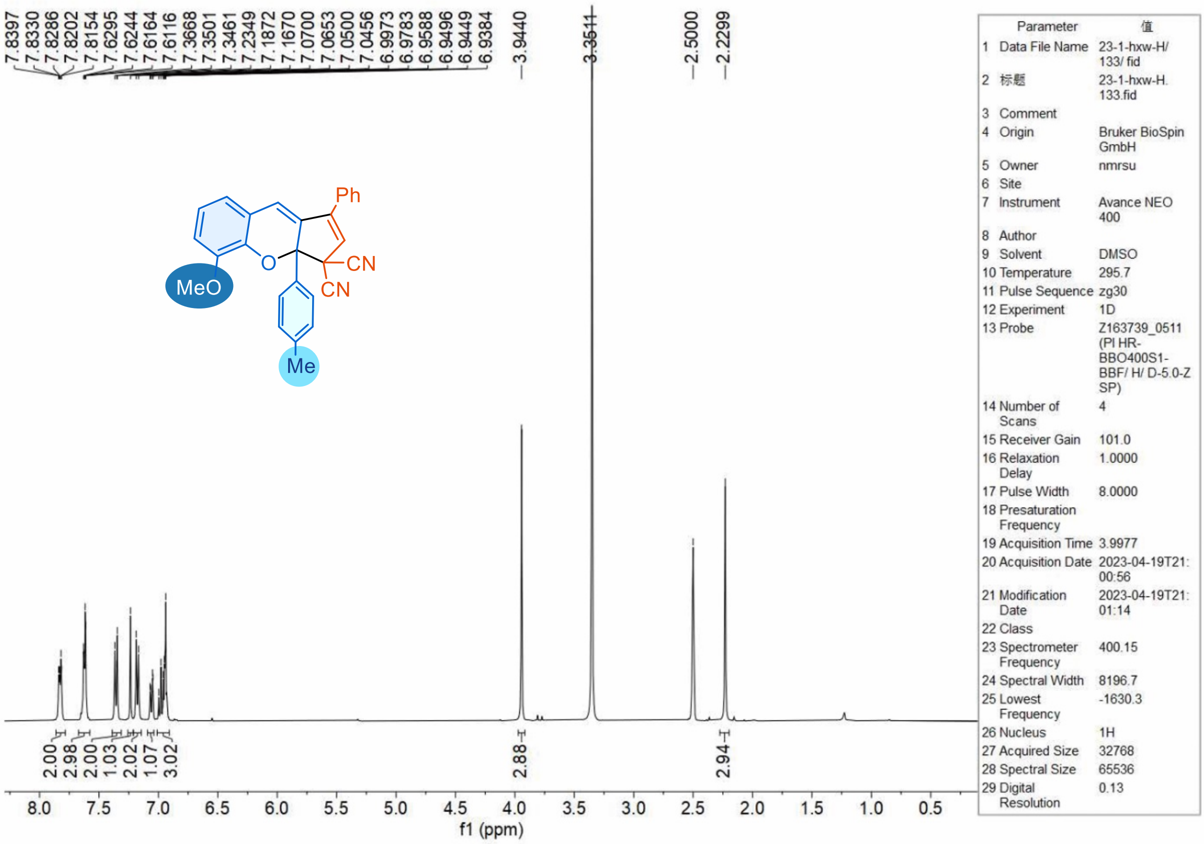

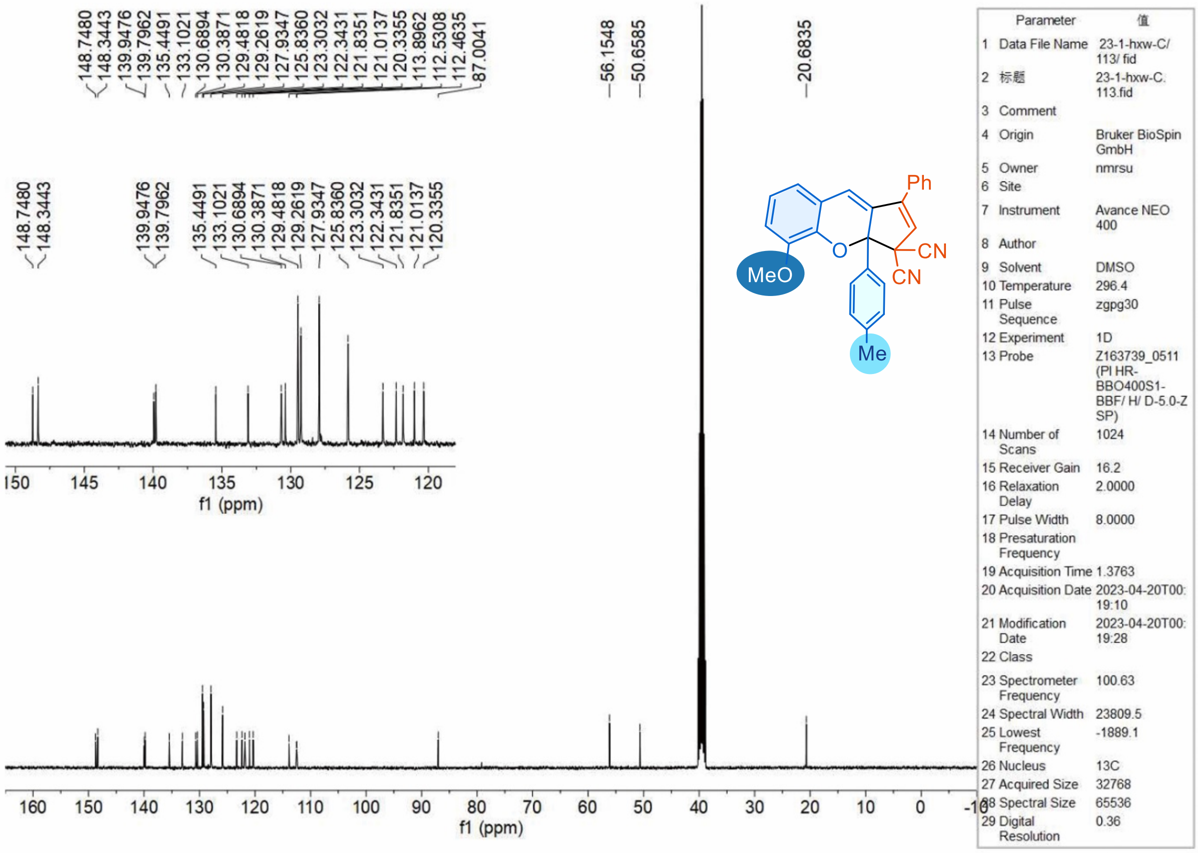

7-Bromo-3a-(4-ethylphenyl)-1-phenylcyclopenta[*b*]chromene-3,3(3a*H*)-dicarbonitrile (product 3ta)

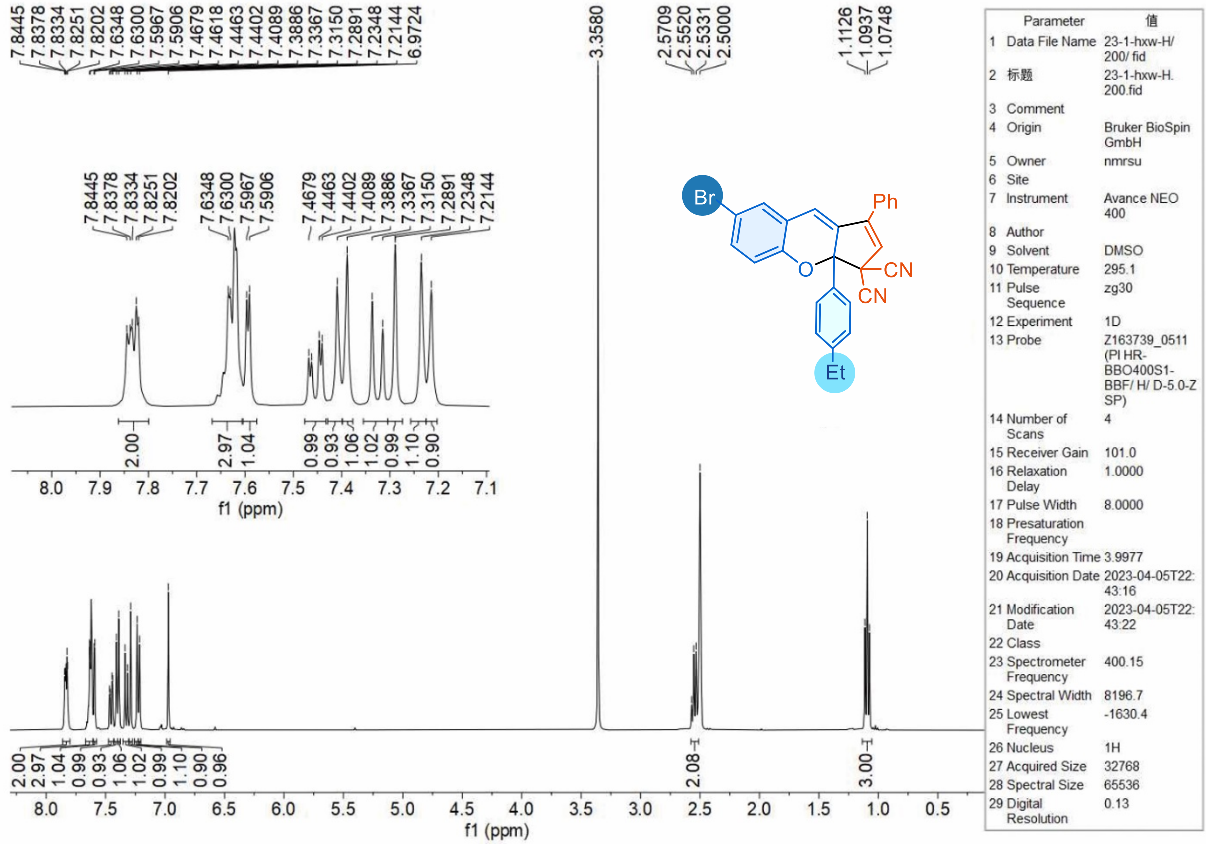

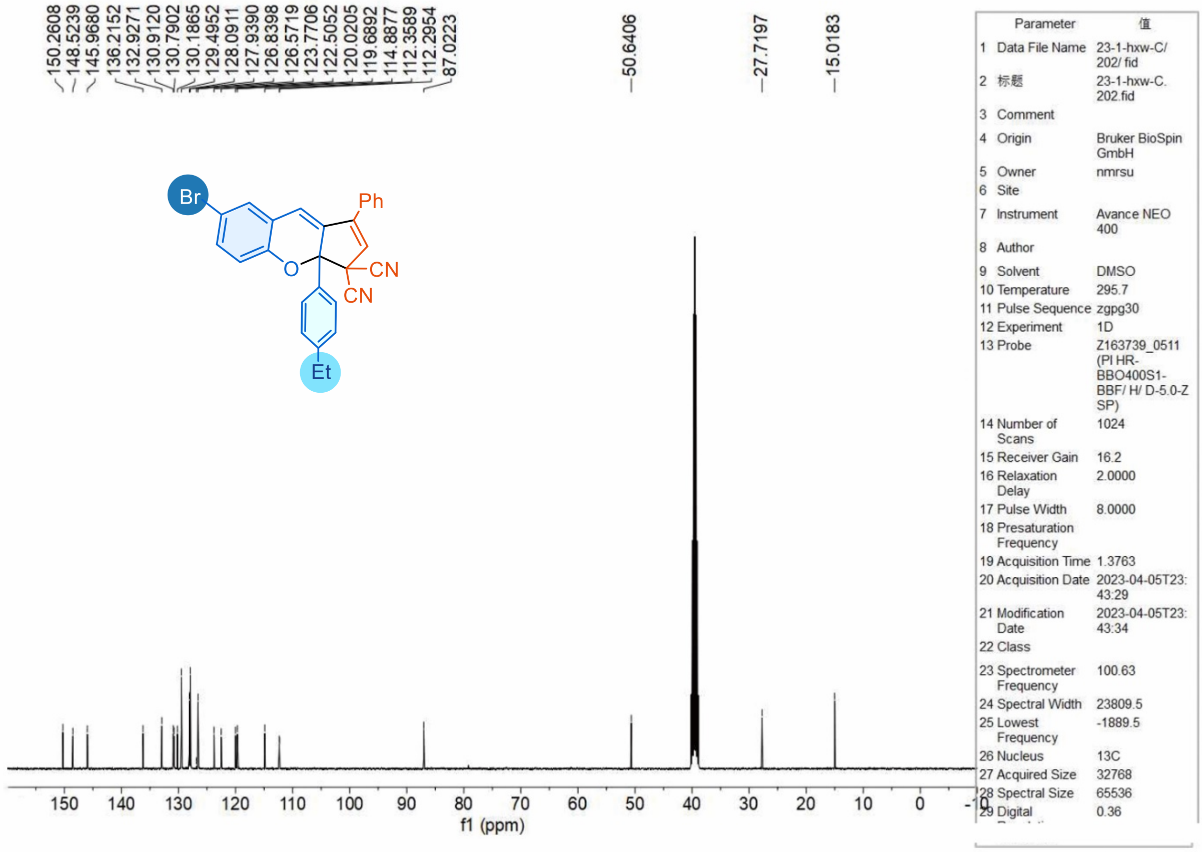

3a-(4-Chlorophenyl)-7-methoxy-1-phenylcyclopenta[*b*]chromene-3,3(3a*H*)-dicarbonitrile (product 3ua)

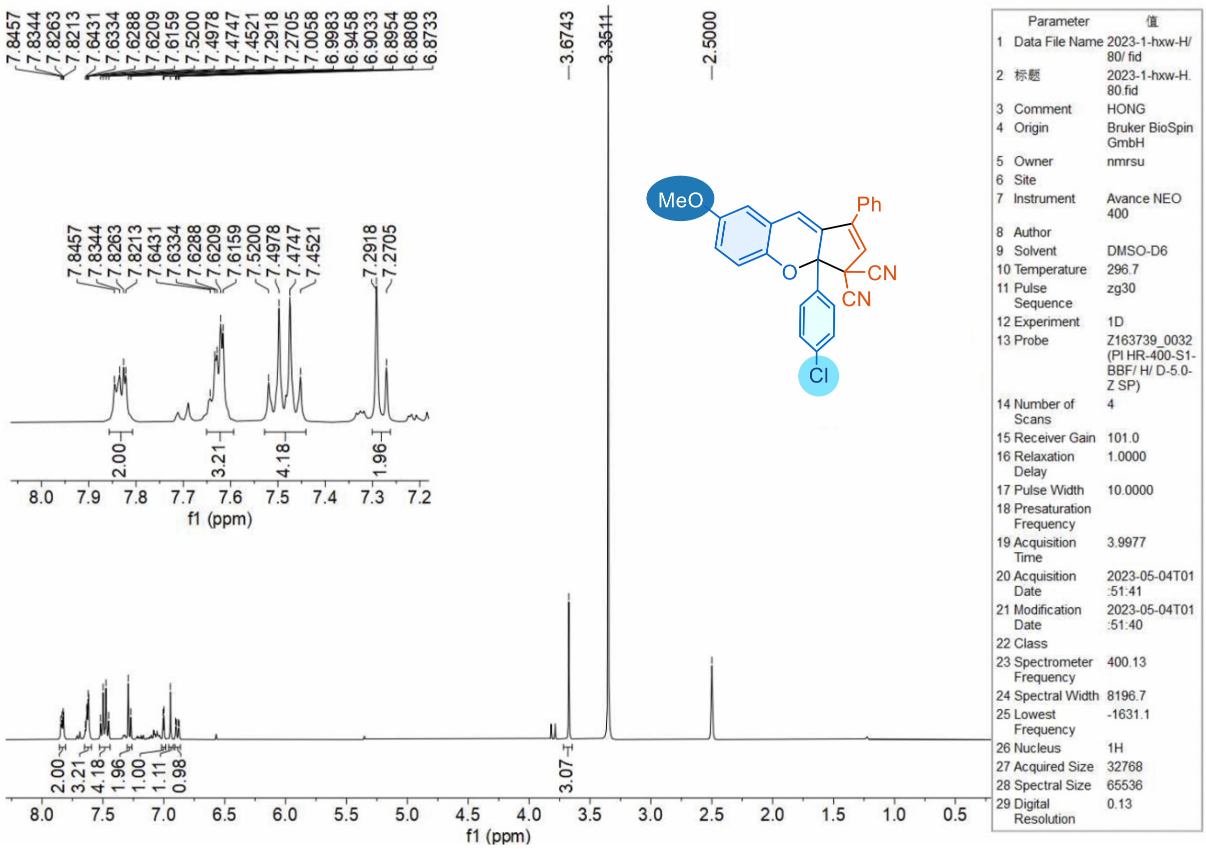

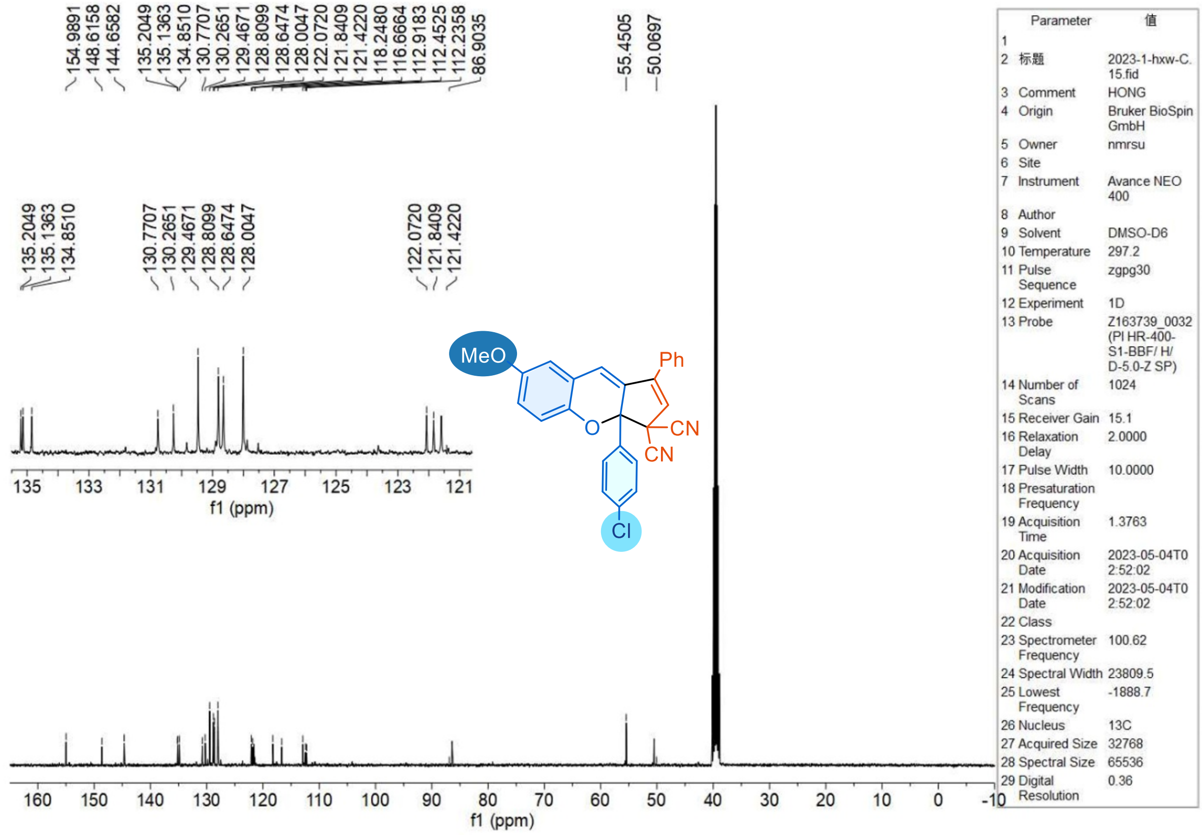

3a-(4-Chlorophenyl)-7-methyl-1-phenylcyclopenta[*b*]chromene-3,3(3a*H*)-dicarbonitrile (product 3va)

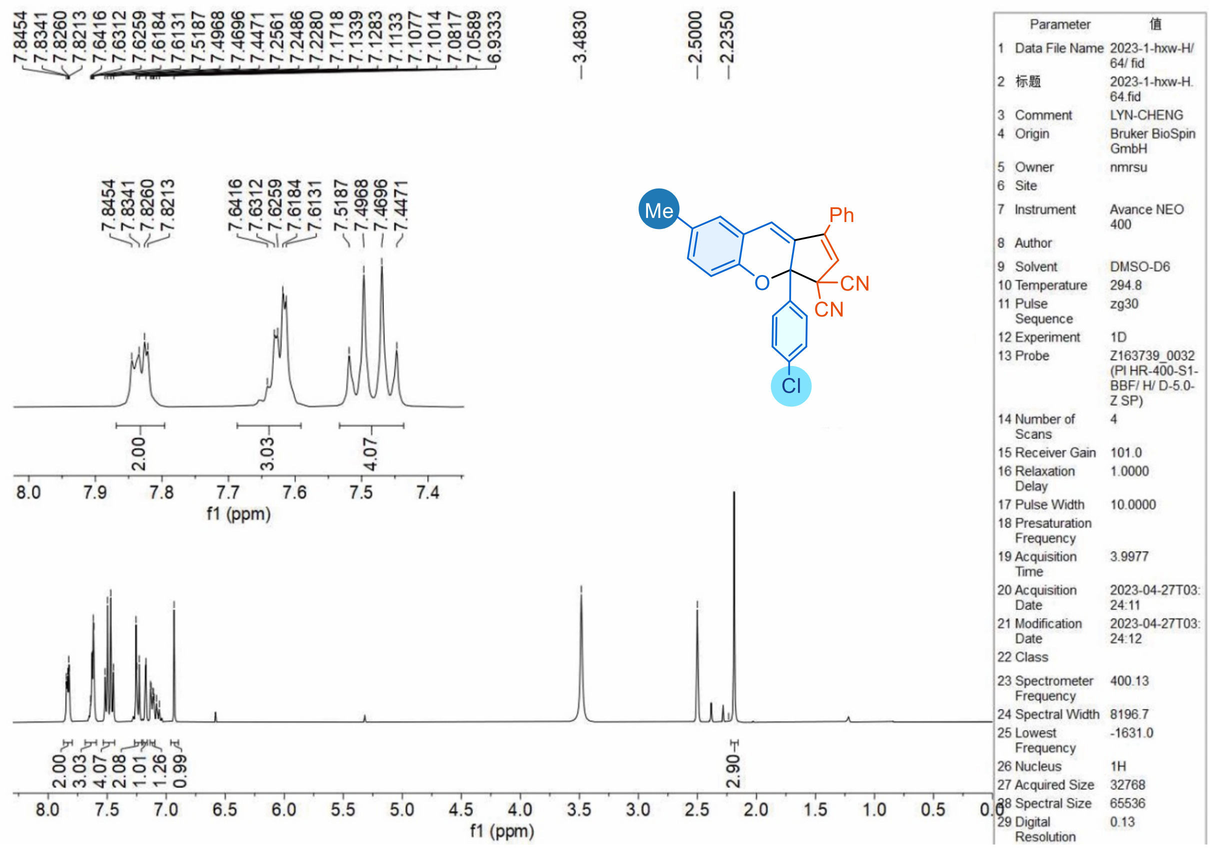

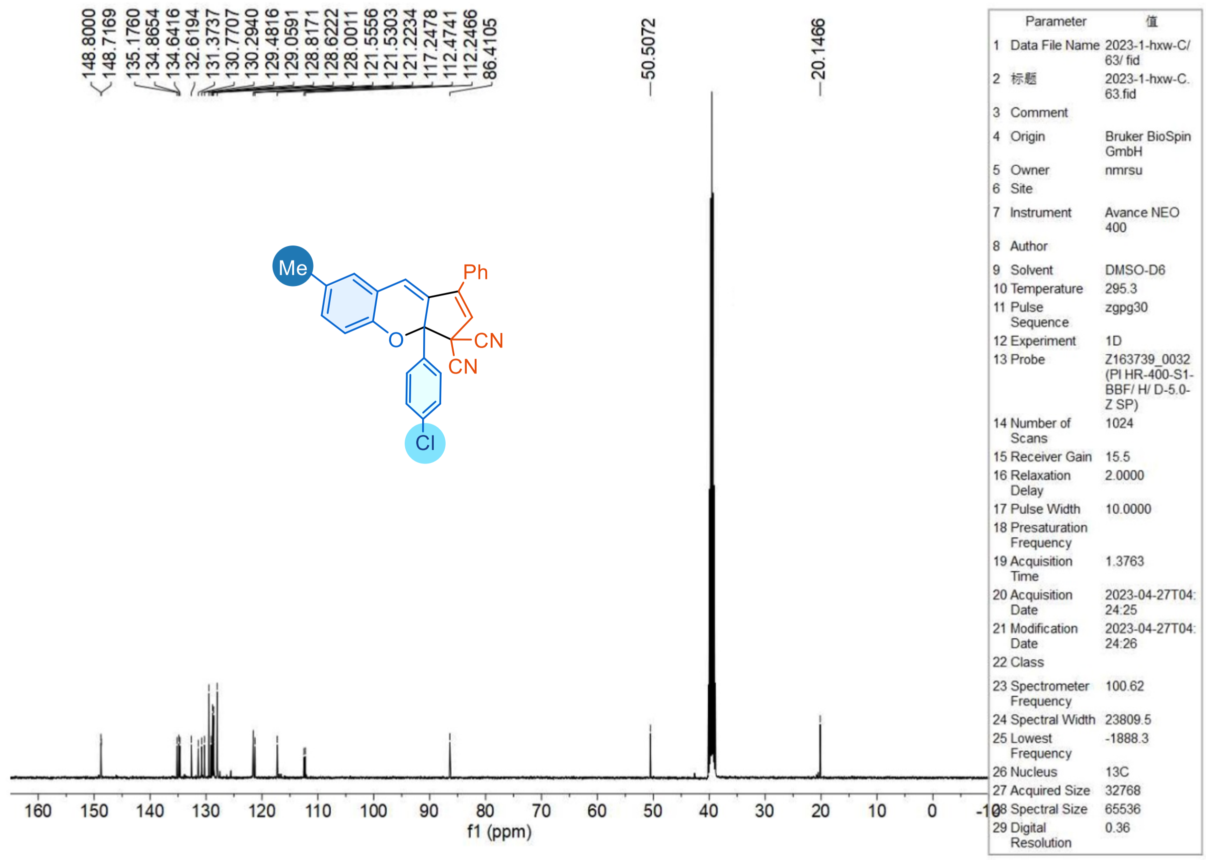

7-Chloro-3a-(4-methoxyphenyl)-1-phenylcyclopenta[*b*]chromene-3,3(3a*H*)-dicarbonitrile (product 3wa)

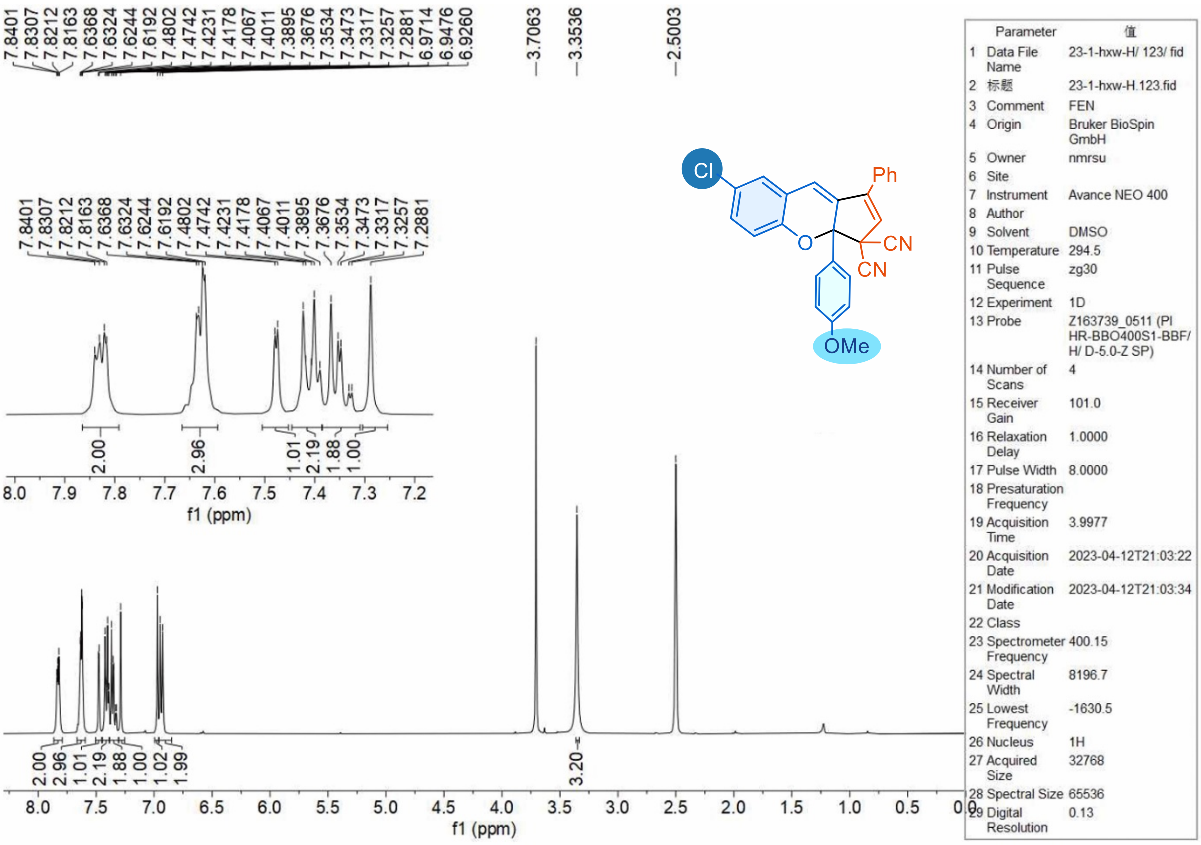

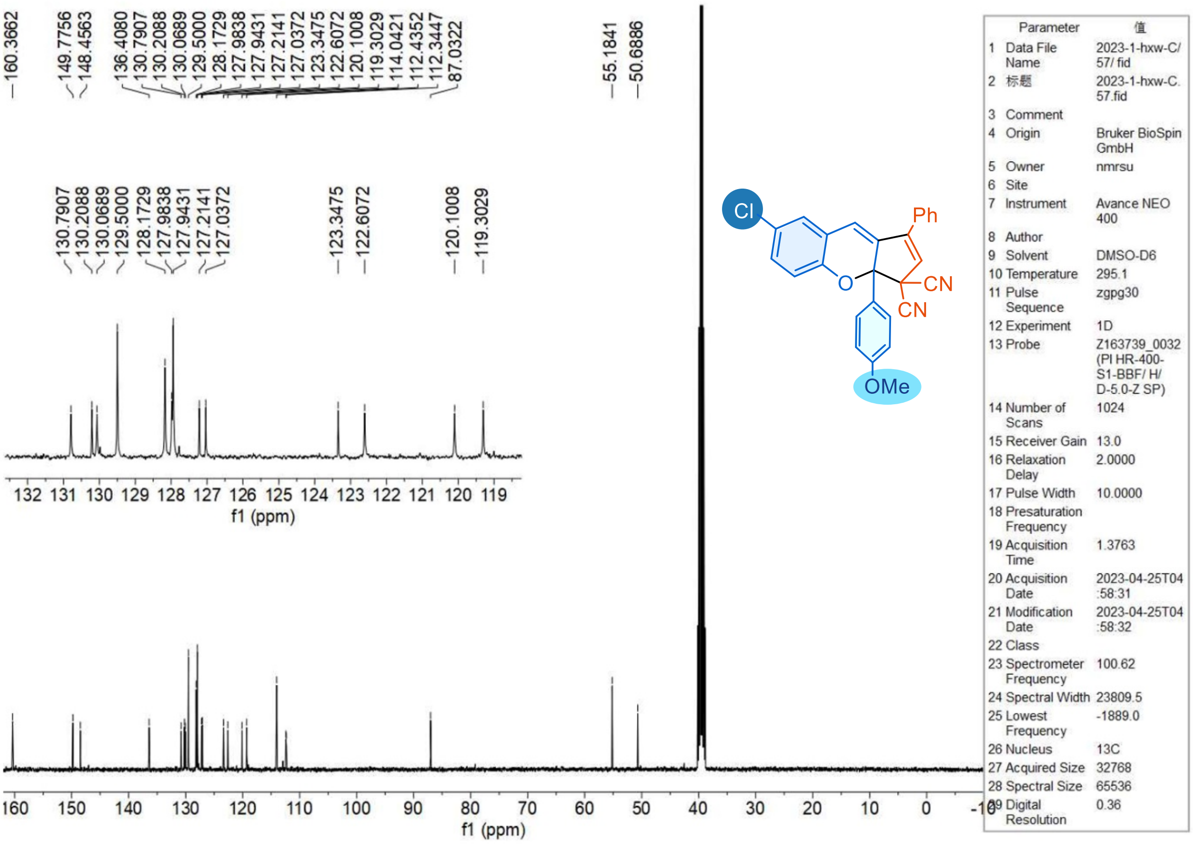

1-Phenyl-3a-(thiophen-3-yl)cyclopenta[*b*]chromene-3,3(3a*H*)-dicarbonitrile (product 3xa)

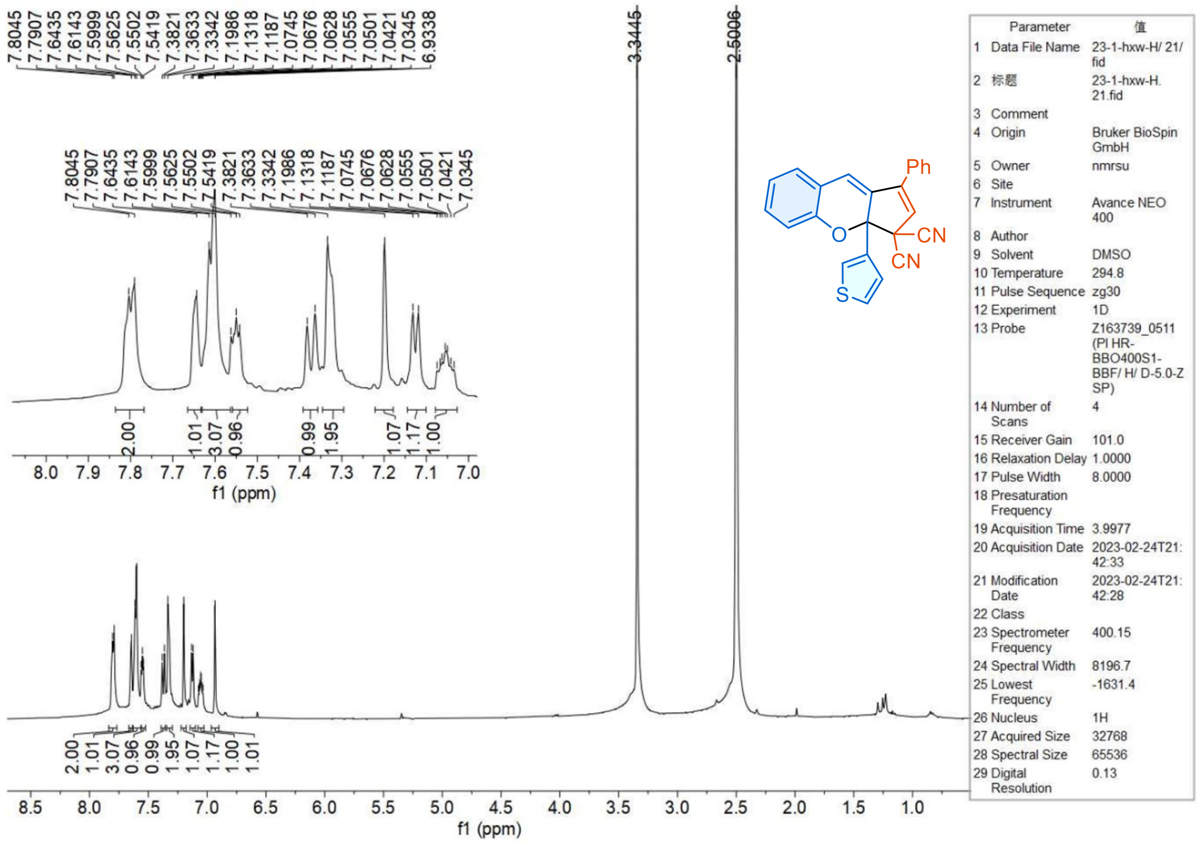

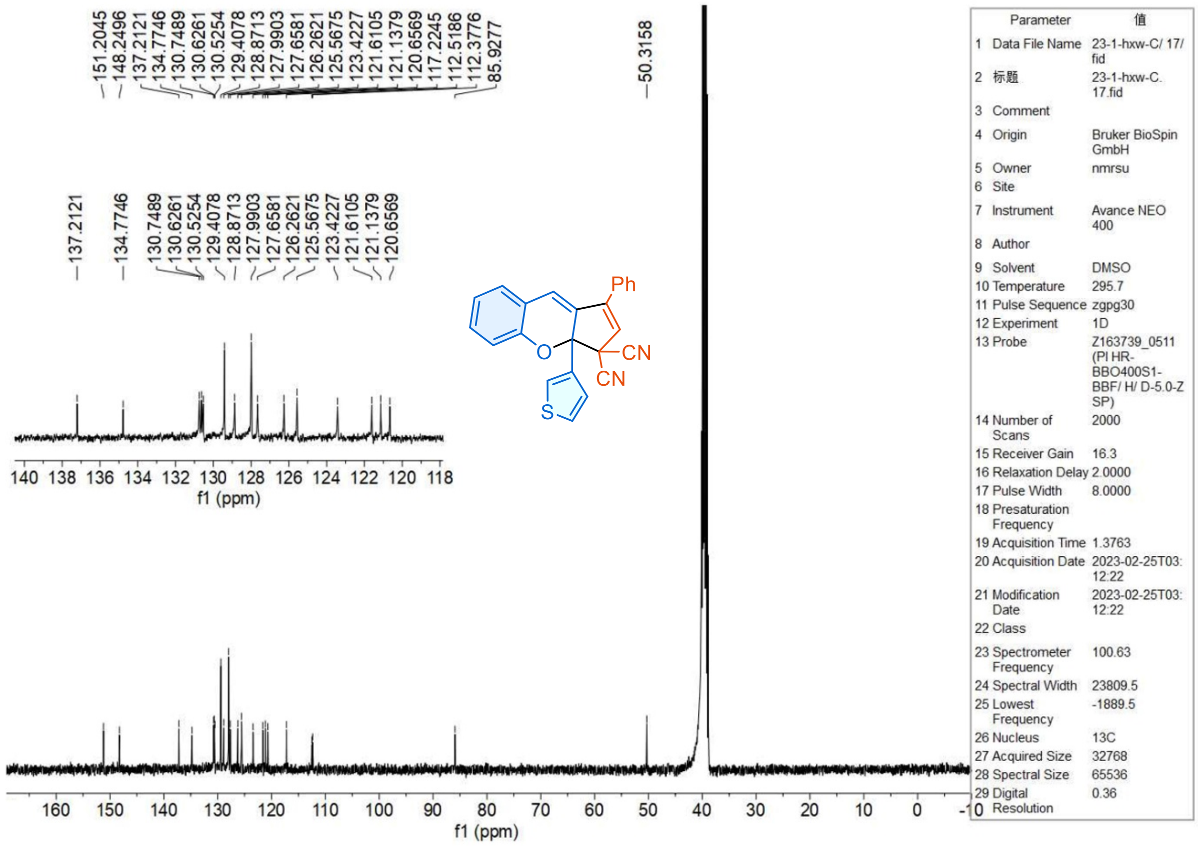

1-Phenyl-3a-(thiophen-2-yl)cyclopenta[*b*]chromene-3,3(3a*H*)-dicarbonitrile (product 3ya)

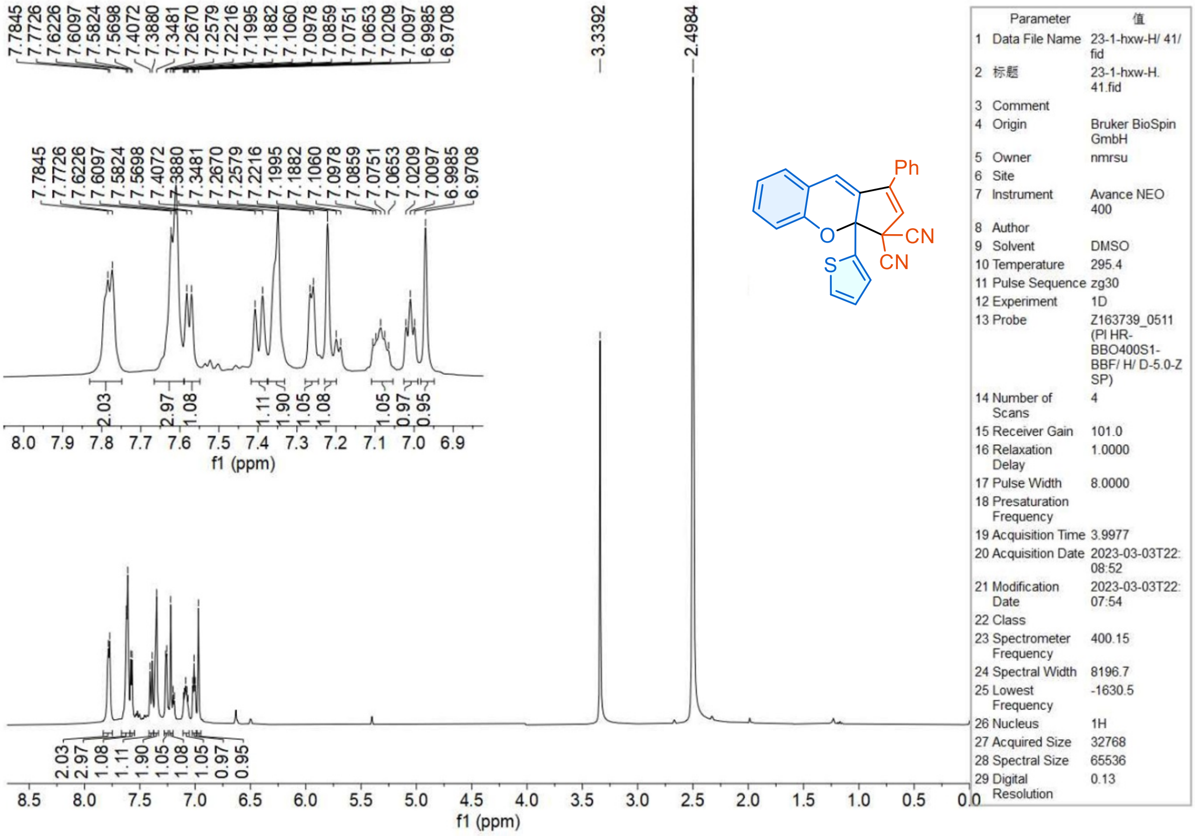

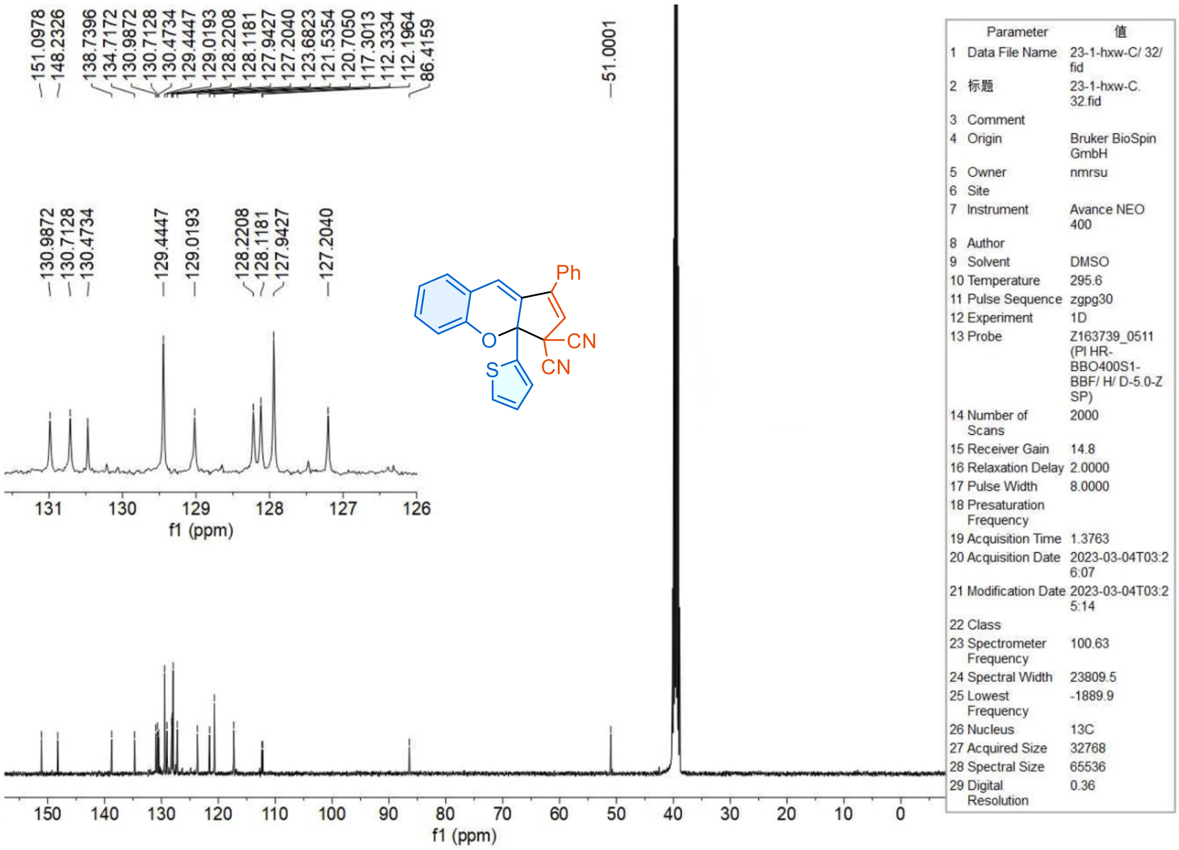

1-(4-Fluorophenyl)-3a-phenylcyclopenta[*b*]chromene-3,3(3a*H*)-dicarbonitrile (product 3ab)

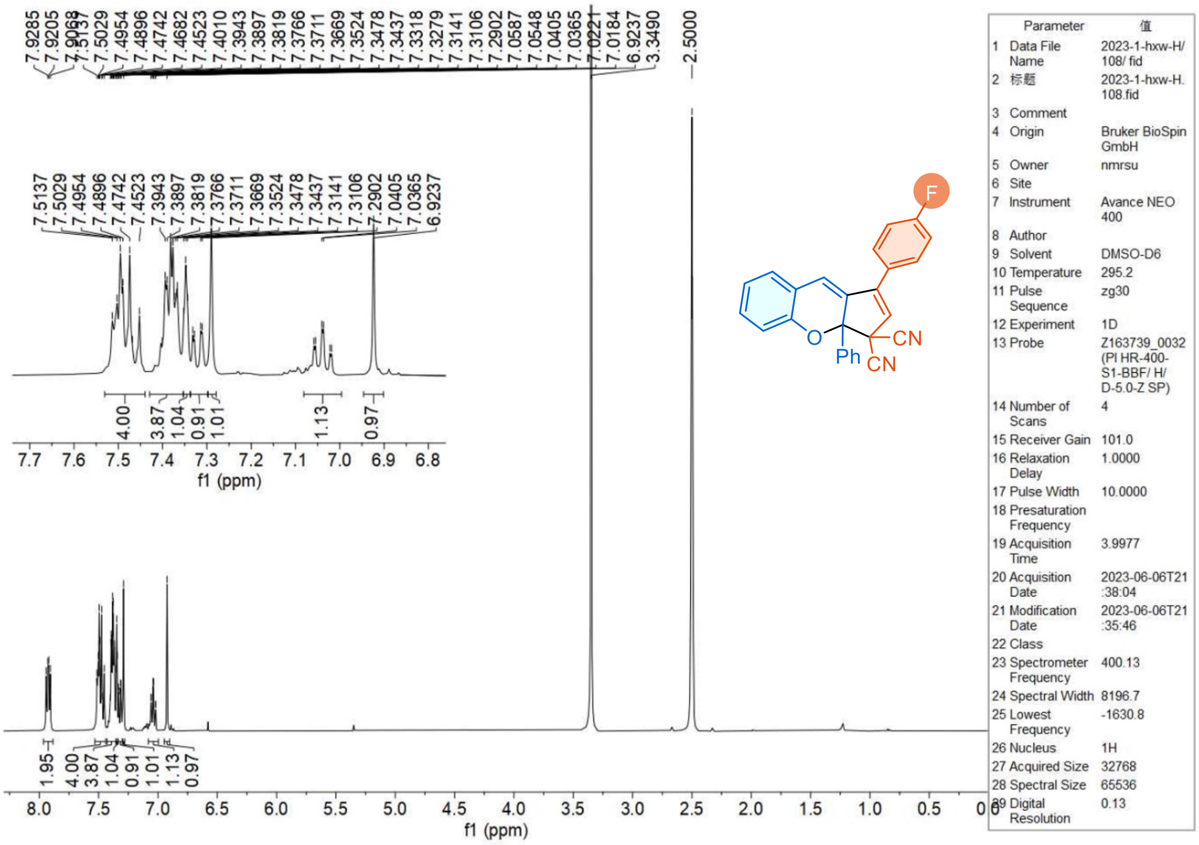

Parameter	值
1 Data File Name	2023-1-hxw-H/ 108/ fid
2 标题	2023-1-hxw-H. 108.fid
3 Comment	
4 Origin	Bruker BioSpin GmbH
5 Owner	nmrsu
6 Site	
7 Instrument	Avance NEO 400
8 Author	
9 Solvent	DMSO-D6
10 Temperature	295.2
11 Pulse Sequence	zg30
12 Experiment	1D
13 Probe	Z163739_0032 (PI HR-400-S1-BBF/ H/ D-5.0-Z SP)
14 Number of Scans	4
15 Receiver Gain	101.0
16 Relaxation Delay	1.0000
17 Pulse Width	10.0000
18 Presaturation Frequency	
19 Acquisition Time	3.9977
20 Acquisition Date	2023-06-06T21:38:04
21 Modification Date	2023-06-06T21:35:46
22 Class	
23 Spectrometer Frequency	400.13
24 Spectral Width	8196.7
25 Lowest Frequency	-1630.8
26 Nucleus	1H
27 Acquired Size	32768
28 Spectral Size	65536
29 Digital Resolution	0.13

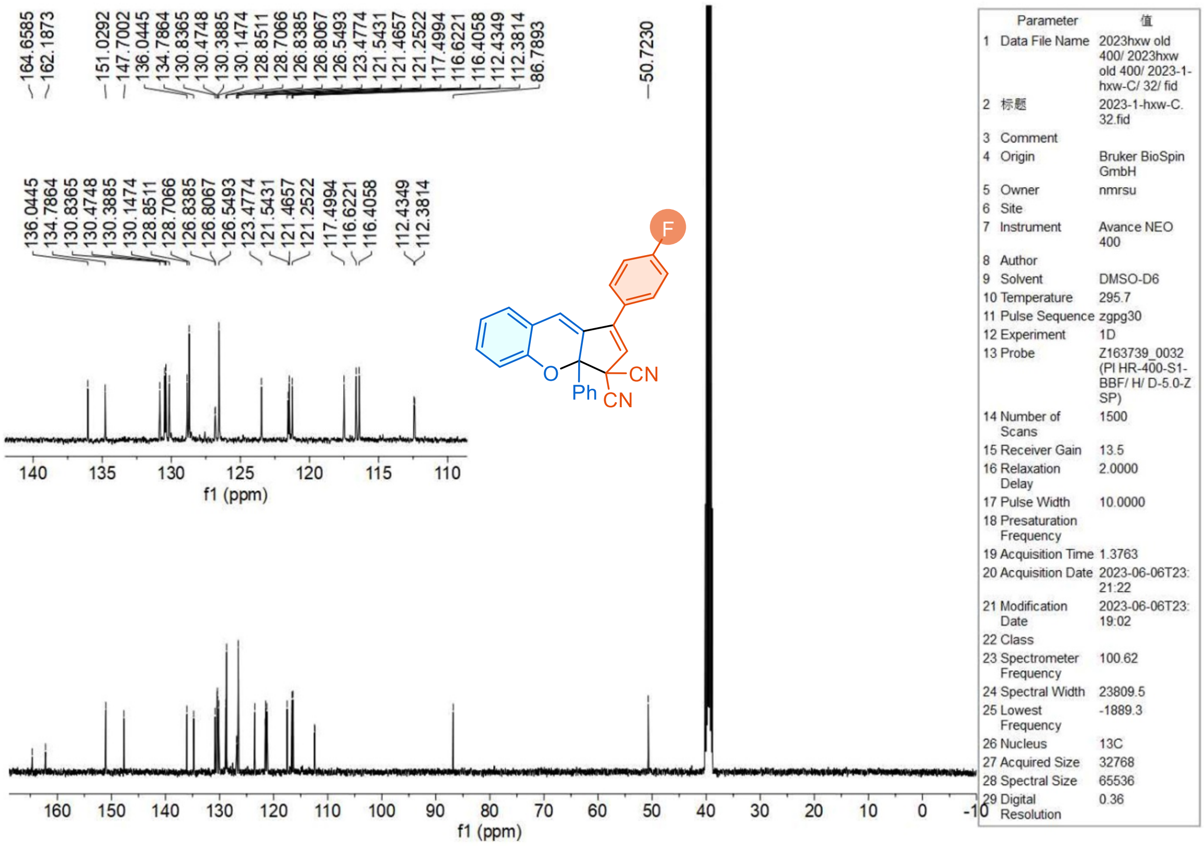

Parameter	值
1 Data File Name	2023hxw old 400/ 2023hxw old 400/ 2023-1-hxw-C/ 32/ fid
2 标题	2023-1-hxw-C. 32.fid
3 Comment	
4 Origin	Bruker BioSpin GmbH
5 Owner	nmrsu
6 Site	
7 Instrument	Avance NEO 400
8 Author	
9 Solvent	DMSO-D6
10 Temperature	295.7
11 Pulse Sequence	zgpg30
12 Experiment	1D
13 Probe	Z163739_0032 (PI HR-400-S1-BBF/ H/ D-5.0-Z SP)
14 Number of Scans	1500
15 Receiver Gain	13.5
16 Relaxation Delay	2.0000
17 Pulse Width	10.0000
18 Presaturation Frequency	
19 Acquisition Time	1.3763
20 Acquisition Date	2023-06-06T23:21:22
21 Modification Date	2023-06-06T23:19:02
22 Class	
23 Spectrometer Frequency	100.62
24 Spectral Width	23809.5
25 Lowest Frequency	-1889.3
26 Nucleus	13C
27 Acquired Size	32768
28 Spectral Size	65536
29 Digital Resolution	0.36

1-(4-Fluorophenyl)-3a-phenylcyclopenta[*b*]chromene-3,3(3a*H*)-dicarbonitrile (product 3ab)

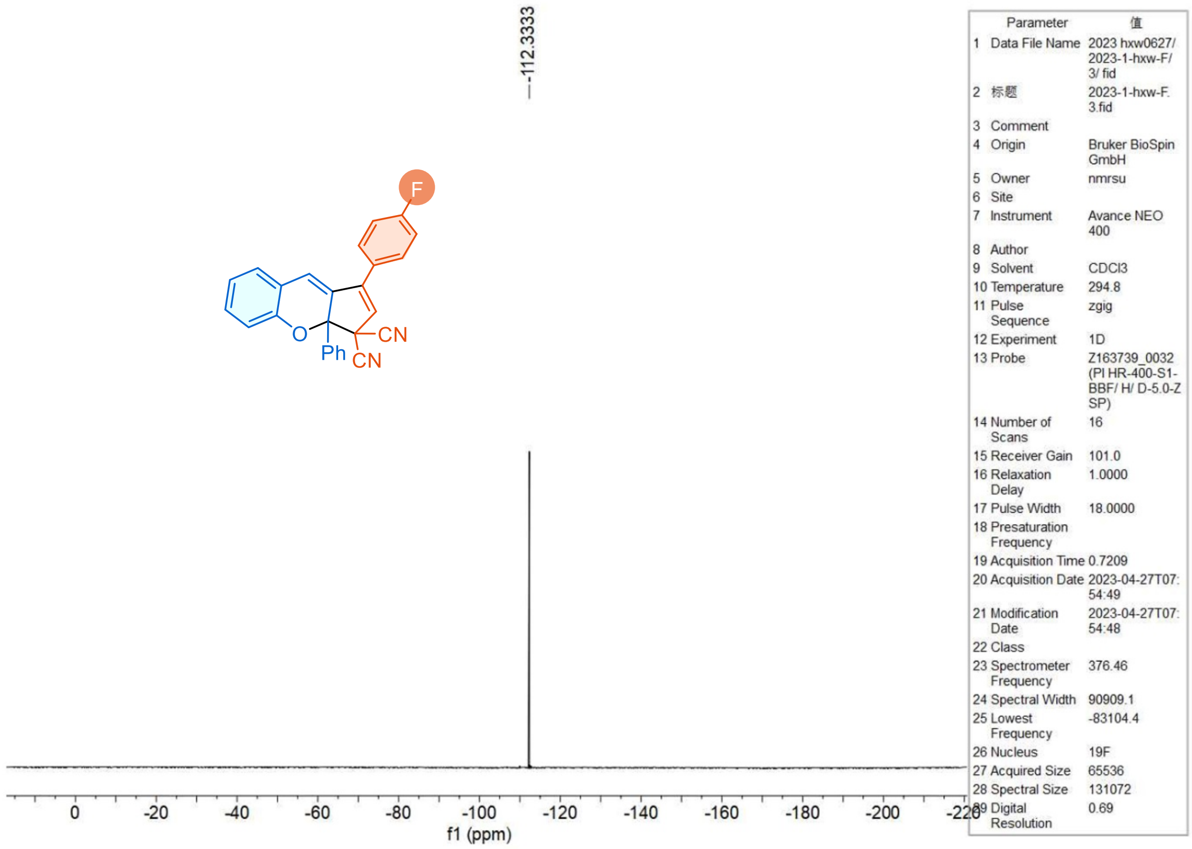

	Parameter	值
1	Data File Name	2023 hxw0627/ 2023-1-hxw-F/ 3/ fid
2	标题	2023-1-hxw-F. 3.fid
3	Comment	
4	Origin	Bruker BioSpin GmbH
5	Owner	nmrsu
6	Site	
7	Instrument	Avance NEO 400
8	Author	
9	Solvent	CDCl3
10	Temperature	294.8
11	Pulse Sequence	zgig
12	Experiment	1D
13	Probe	Z163739_0032 (PI HR-400-S1-BBF/ H/ D-5.0-Z SP)
14	Number of Scans	16
15	Receiver Gain	101.0
16	Relaxation Delay	1.0000
17	Pulse Width	18.0000
18	Presaturation Frequency	
19	Acquisition Time	0.7209
20	Acquisition Date	2023-04-27T07: 54:49
21	Modification Date	2023-04-27T07: 54:48
22	Class	
23	Spectrometer Frequency	376.46
24	Spectral Width	90909.1
25	Lowest Frequency	-83104.4
26	Nucleus	19F
27	Acquired Size	65536
28	Spectral Size	131072
29	Digital Resolution	0.69

1-([1,1'-Biphenyl]-4-yl)-3a-phenylcyclopenta[*b*]chromene-3,3(3a*H*)-dicarbonitrile (product 3ac)

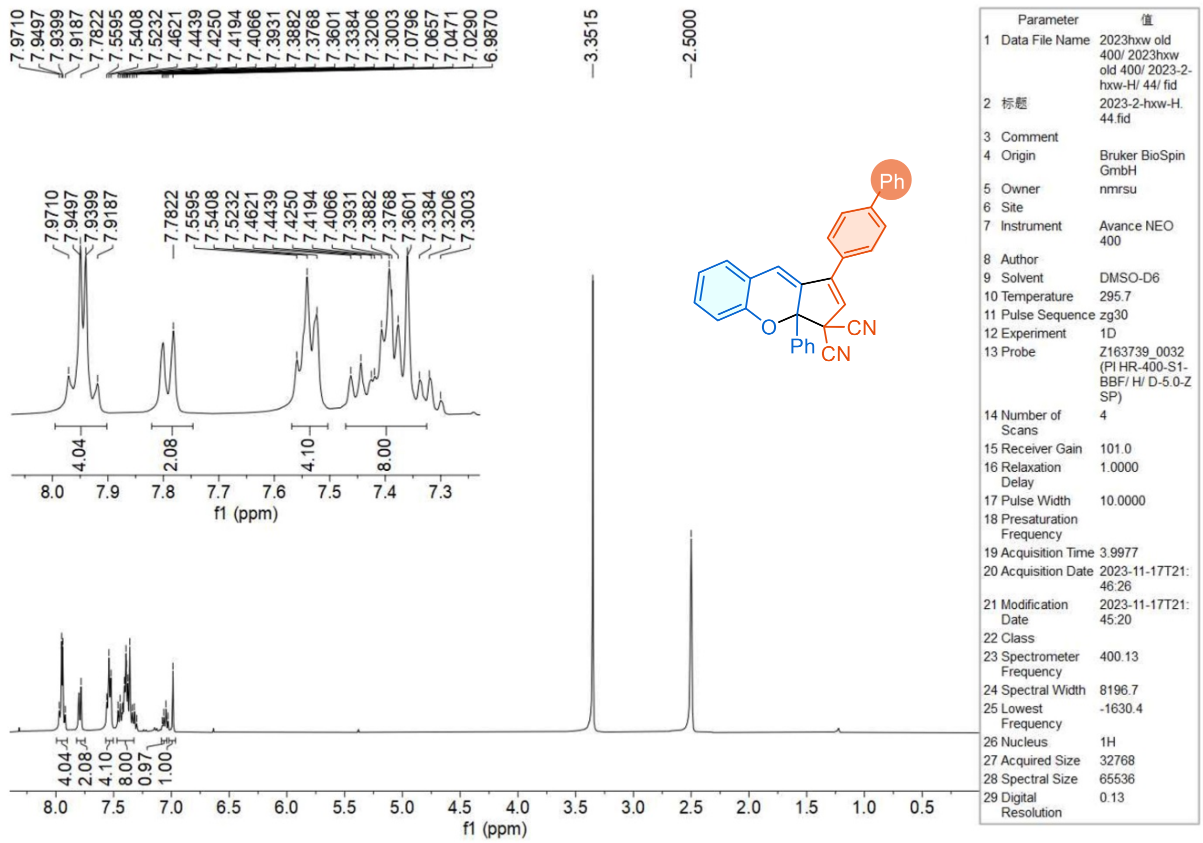

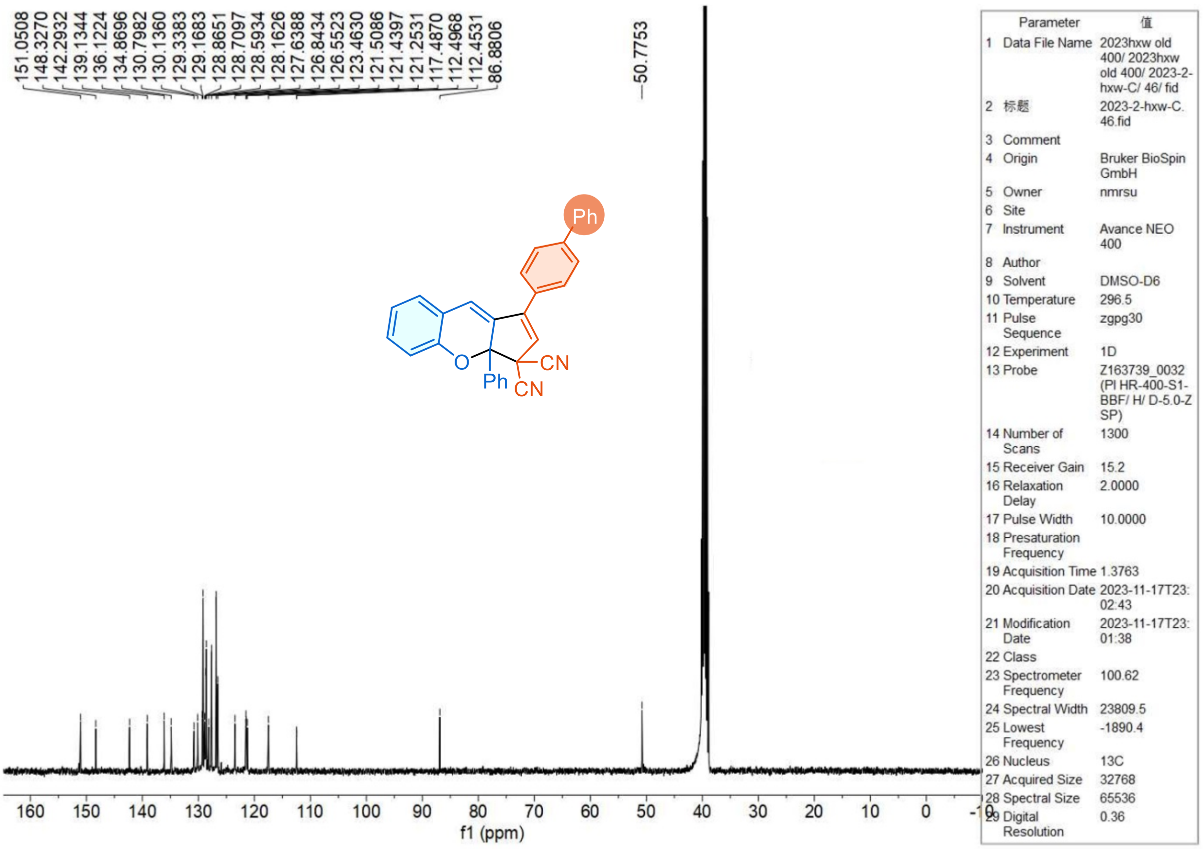

3a-Phenyl-1-(*p*-tolyl)cyclopenta[*b*]chromene-3,3(3a*H*)-dicarbonitrile (product 3ad)

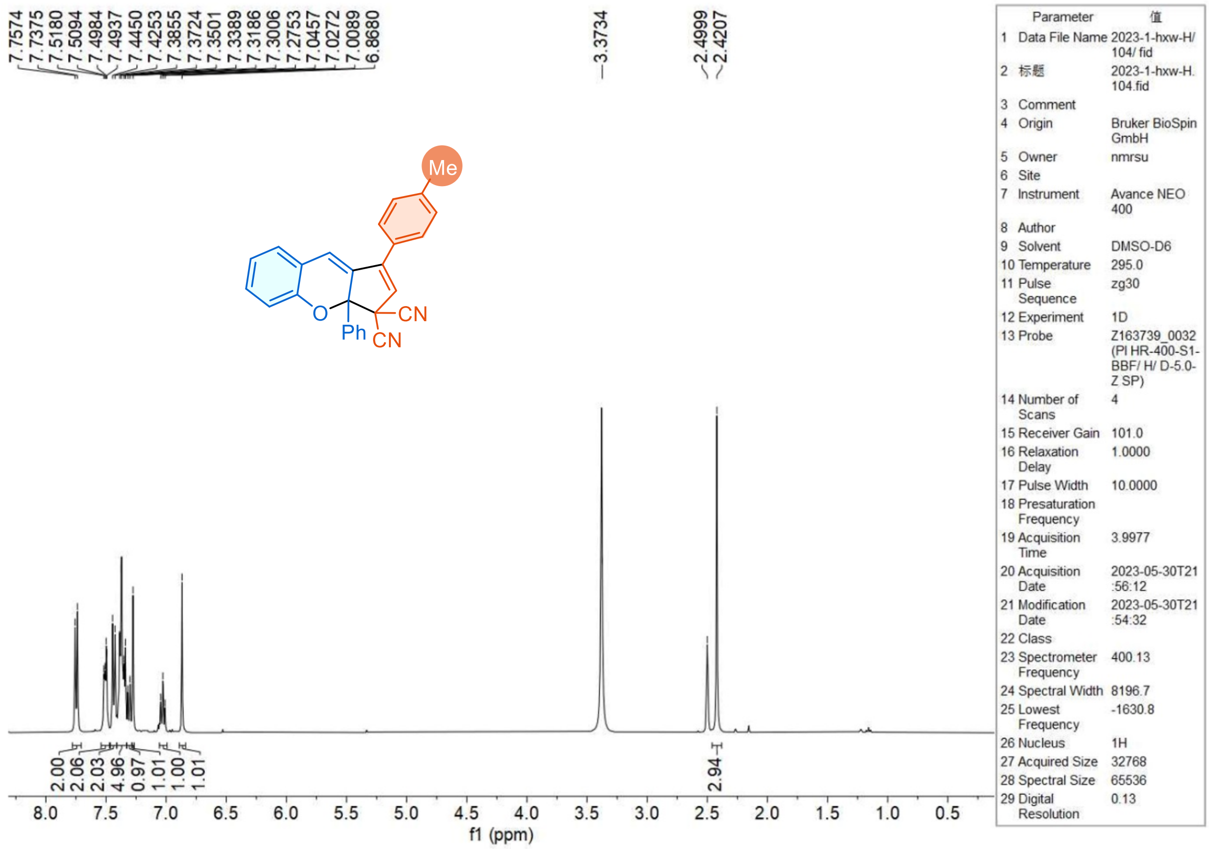

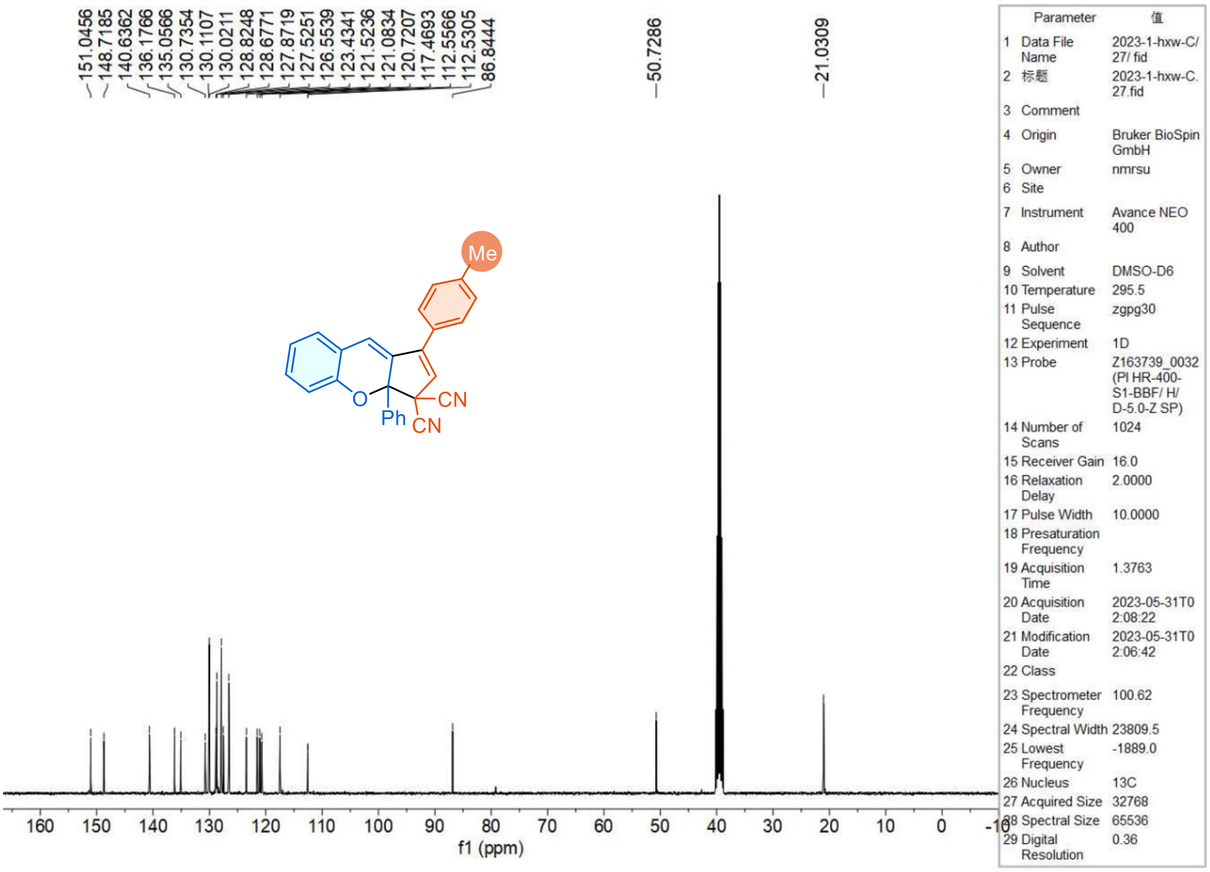

1-(3-Bromophenyl)-3a-phenylcyclopenta[*b*]chromene-3,3(3a*H*)-dicarbonitrile (product 3ae)

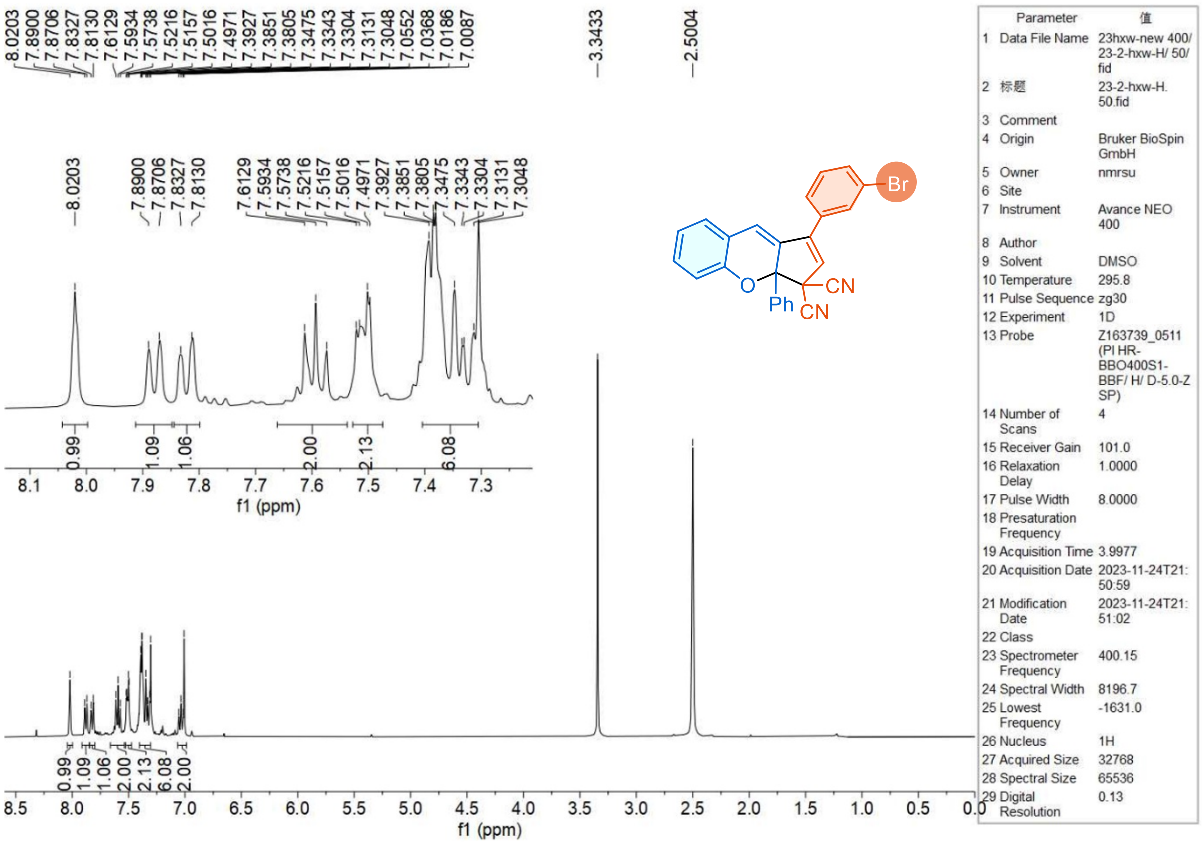

Parameter	值
1 Data File Name	23hxw-new 400/ 23-2-hxw-H/ 50/ fid
2 标题	23-2-hxw-H. 50.fid
3 Comment	
4 Origin	Bruker BioSpin GmbH
5 Owner	nmrsu
6 Site	
7 Instrument	Avance NEO 400
8 Author	
9 Solvent	DMSO
10 Temperature	295.8
11 Pulse Sequence	zg30
12 Experiment	1D
13 Probe	Z163739_0511 (PI HR-BBO400S1-BBF/ H/ D-5.0-Z SP)
14 Number of Scans	4
15 Receiver Gain	101.0
16 Relaxation Delay	1.0000
17 Pulse Width	8.0000
18 Presaturation Frequency	
19 Acquisition Time	3.9977
20 Acquisition Date	2023-11-24T21:50:59
21 Modification Date	2023-11-24T21:51:02
22 Class	
23 Spectrometer Frequency	400.15
24 Spectral Width	8196.7
25 Lowest Frequency	-1631.0
26 Nucleus	1H
27 Acquired Size	32768
28 Spectral Size	65536
29 Digital Resolution	0.13

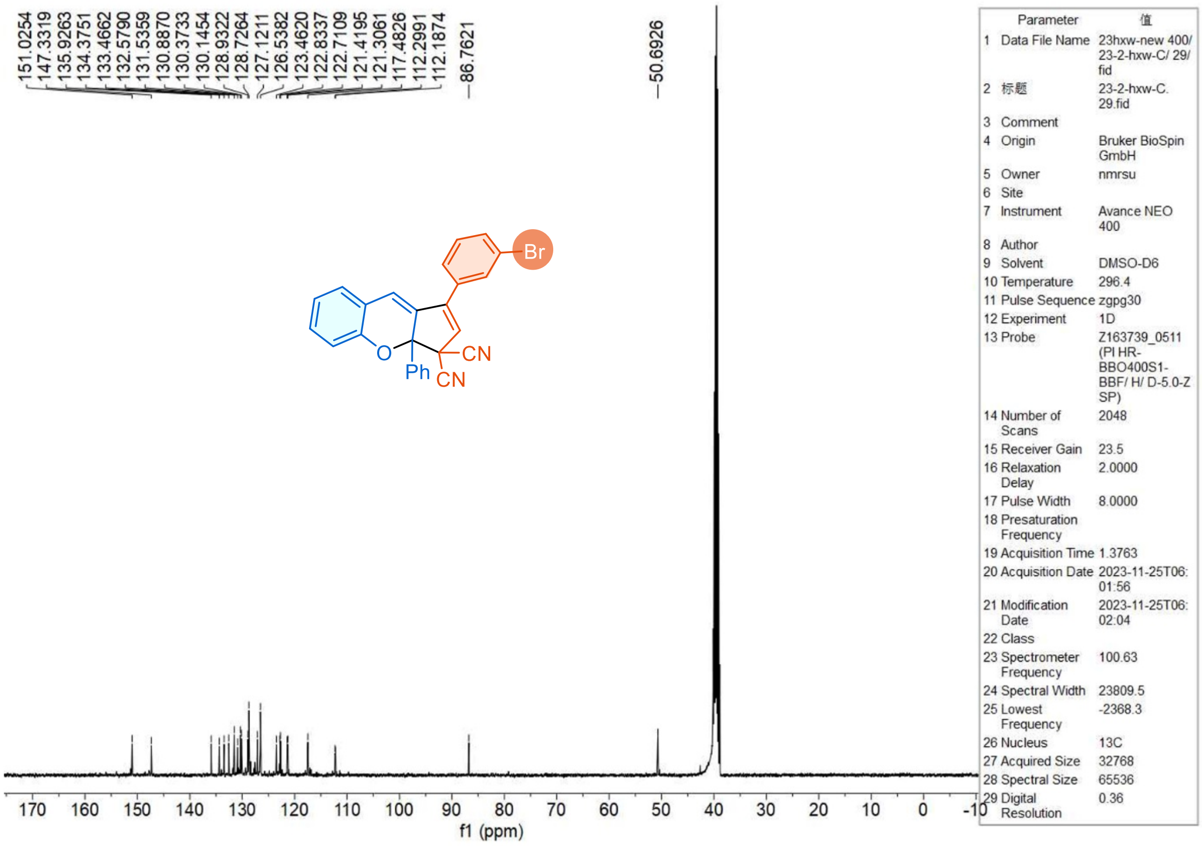

Parameter	值
1 Data File Name	23hxw-new 400/ 23-2-hxw-C/ 29/ fid
2 标题	23-2-hxw-C. 29.fid
3 Comment	
4 Origin	Bruker BioSpin GmbH
5 Owner	nmrsu
6 Site	
7 Instrument	Avance NEO 400
8 Author	
9 Solvent	DMSO-D6
10 Temperature	296.4
11 Pulse Sequence	zgpg30
12 Experiment	1D
13 Probe	Z163739_0511 (PI HR-BBO400S1-BBF/ H/ D-5.0-Z SP)
14 Number of Scans	2048
15 Receiver Gain	23.5
16 Relaxation Delay	2.0000
17 Pulse Width	8.0000
18 Presaturation Frequency	
19 Acquisition Time	1.3763
20 Acquisition Date	2023-11-25T06:01:56
21 Modification Date	2023-11-25T06:02:04
22 Class	
23 Spectrometer Frequency	100.63
24 Spectral Width	23809.5
25 Lowest Frequency	-2368.3
26 Nucleus	13C
27 Acquired Size	32768
28 Spectral Size	65536
29 Digital Resolution	0.36

1-(3,4-Dichlorophenyl)-3a-phenylcyclopenta[*b*]chromene-3,3(3a*H*)-dicarbonitrile (product 3af)

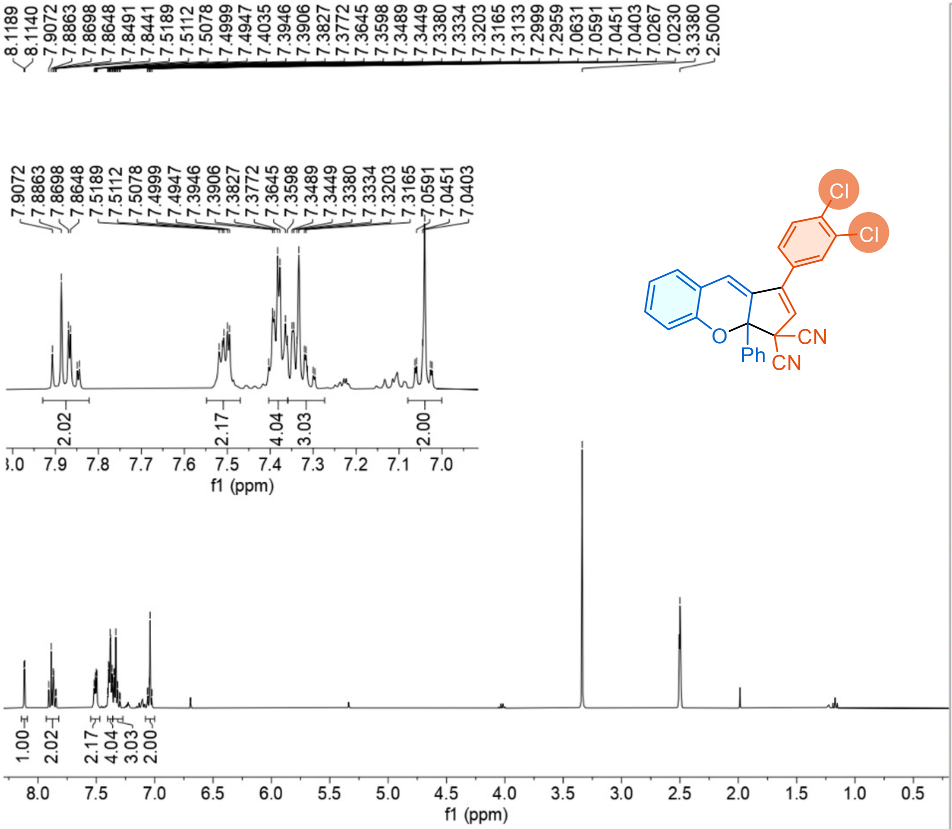

	Parameter	值
1	Data File Name	2023hxw old 400/ 2023hxw old 400/ 2023-2-hxw-H/ 40/ fid
2	标题	2023-2-hxw-H.40.fid
3	Comment	
4	Origin	Bruker BioSpin GmbH
5	Owner	nmrsu
6	Site	
7	Instrument	Avance NEO 400
8	Author	
9	Solvent	DMSO-D6
10	Temperature	295.6
11	Pulse Sequence	zg30
12	Experiment	1D
13	Probe	Z163739_0032 (PI HR-400-S1-BBF/ H/ D-5.0-Z SP)
14	Number of Scans	4
15	Receiver Gain	101.0
16	Relaxation Delay	1.0000
17	Pulse Width	10.0000
18	Presaturation Frequency	
19	Acquisition Time	3.9977
20	Acquisition Date	2023-11-14T01:24:15
21	Modification Date	2023-11-14T01:23:28
22	Class	
23	Spectrometer Frequency	400.13
24	Spectral Width	8196.7
25	Lowest Frequency	-1631.0
26	Nucleus	1H
27	Acquired Size	32768
28	Spectral Size	65536
29	Digital Resolution	0.13

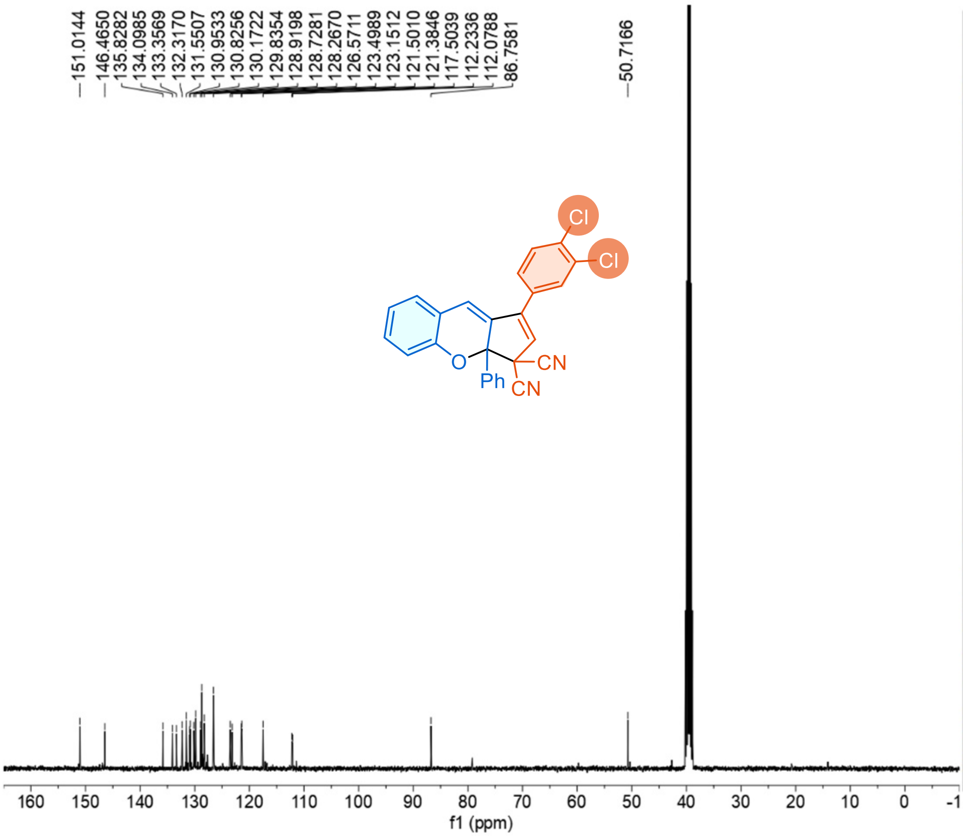

	Parameter	值
1	Data File Name	2023hxw old 400/ 2023-2-hxw-C/ 42/ fid
2	标题	2023-2-hxw-C.42.fid
3	Comment	
4	Origin	Bruker BioSpin GmbH
5	Owner	nmrsu
6	Site	
7	Instrument	Avance NEO 400
8	Author	
9	Solvent	DMSO-D6
10	Temperature	296.1
11	Pulse Sequence	zgpg30
12	Experiment	1D
13	Probe	Z163739_0032 (PI HR-400-S1-BBF/ H/ D-5.0-Z SP)
14	Number of Scans	1024
15	Receiver Gain	16.0
16	Relaxation Delay	2.0000
17	Pulse Width	10.0000
18	Presaturation Frequency	
19	Acquisition Time	1.3763
20	Acquisition Date	2023-11-14T02:24:34
21	Modification Date	2023-11-14T02:23:48
22	Class	
23	Spectrometer Frequency	100.62
24	Spectral Width	23809.5
25	Lowest Frequency	-1890.4
26	Nucleus	13C
27	Acquired Size	32768
28	Spectral Size	65536
29	Digital Resolution	0.36

1-(Naphthalen-1-yl)-3a-phenylcyclopenta[*b*]chromene-3,3(3a*H*)-dicarbonitrile (product 3ag)

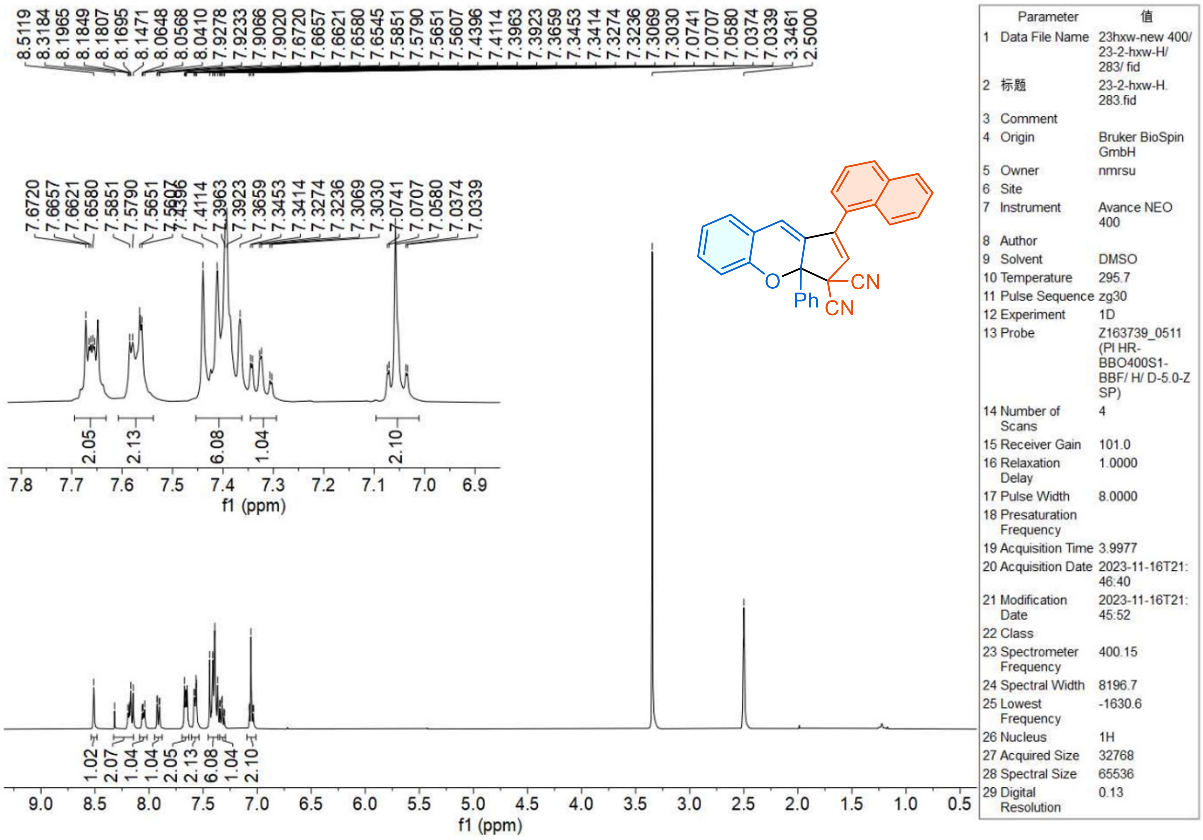

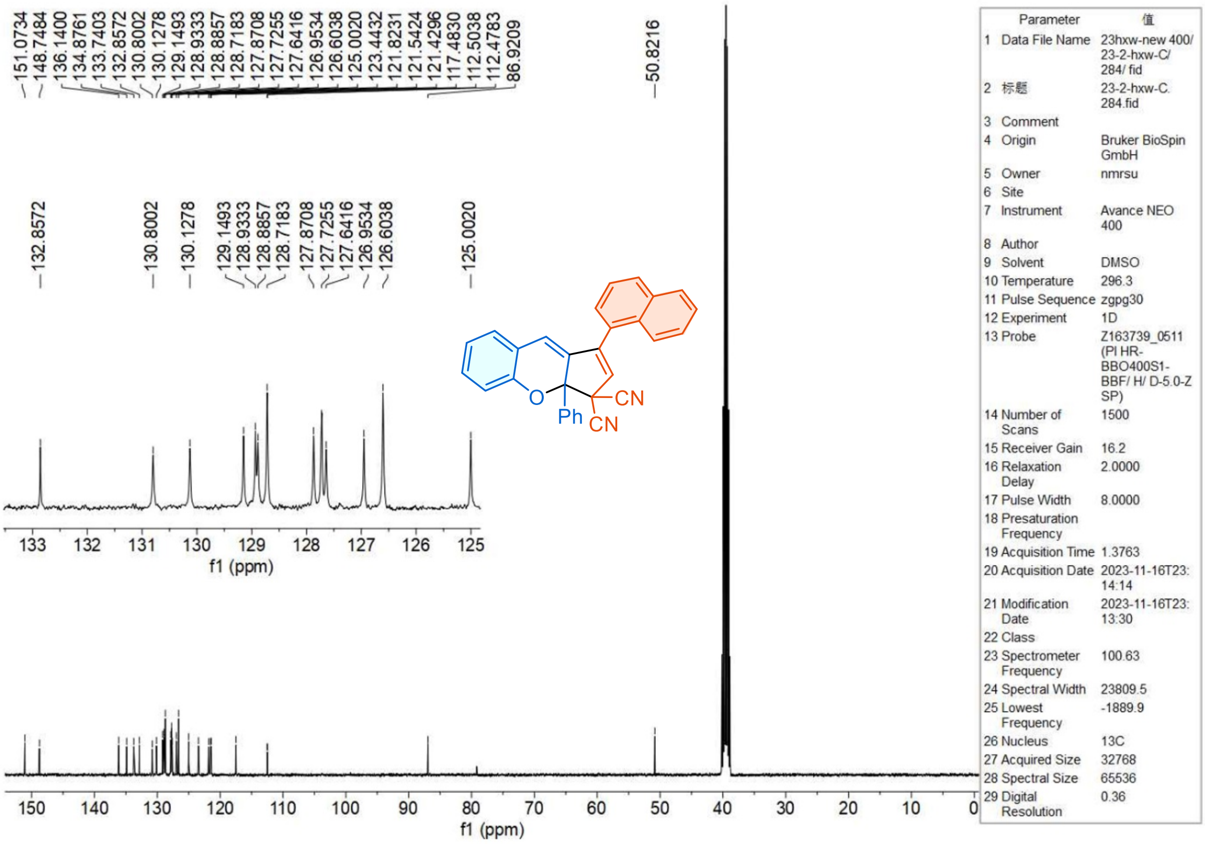

Methyl 3-cyano-1,3a-diphenyl-3,3a-dihydrocyclopenta[*b*]chromene-3-carboxylate (product 5a)

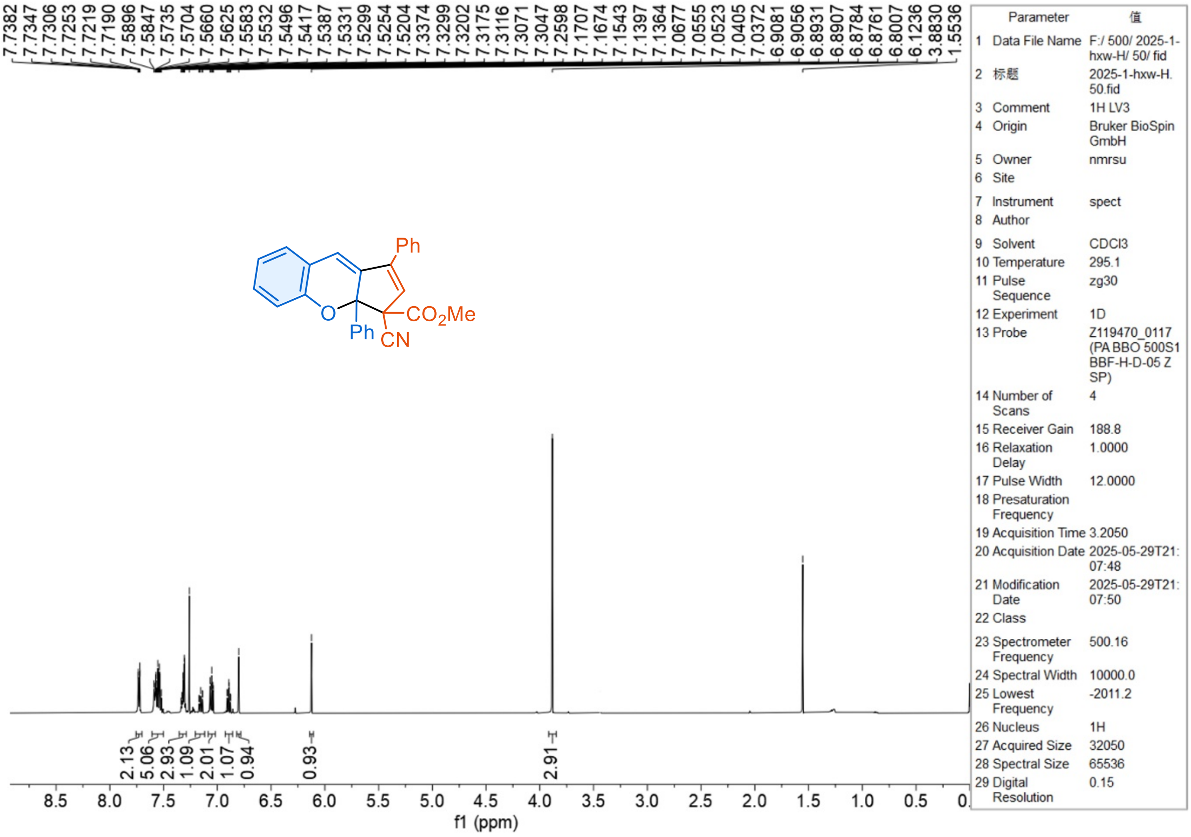

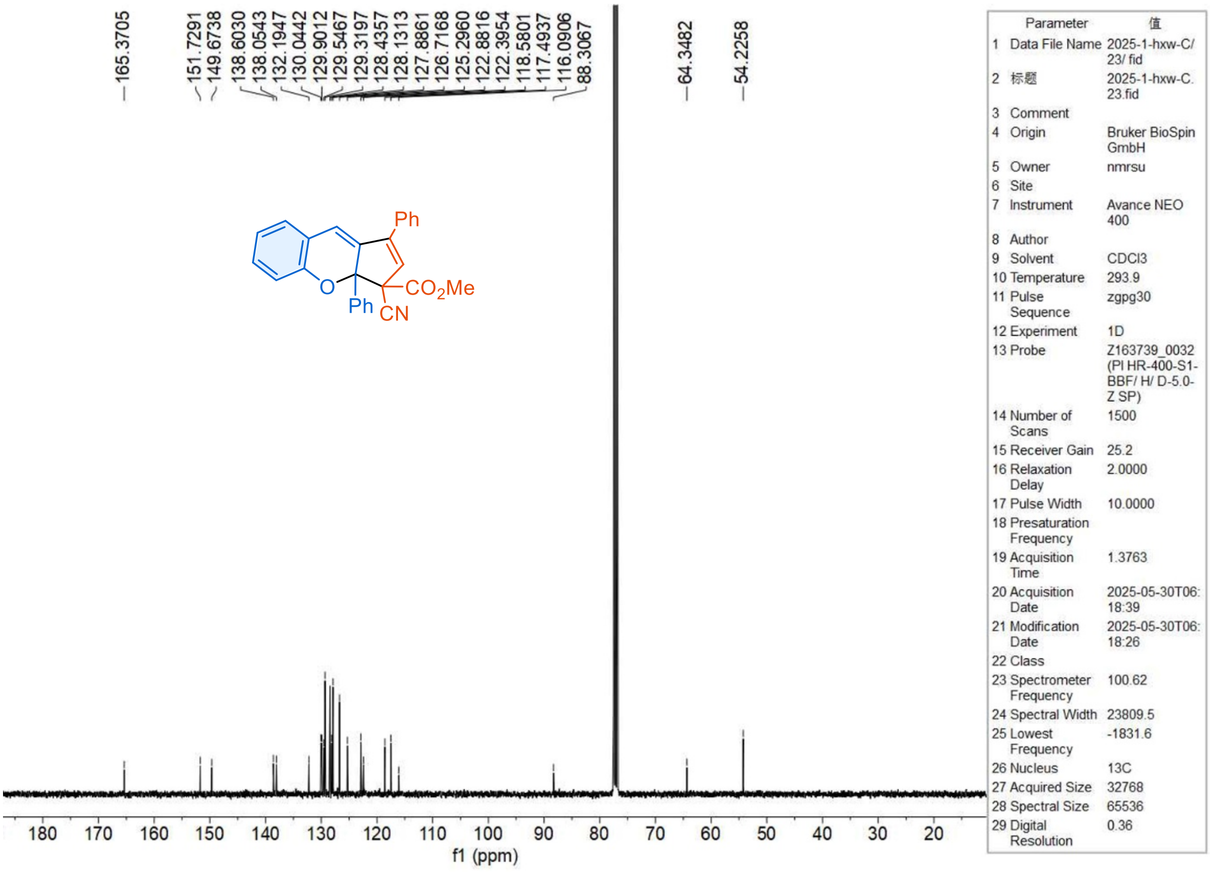

Ethyl 3-cyano-1,3a-diphenyl-3,3a-dihydrocyclopenta[*b*]chromene-3-carboxylate (product 5b)

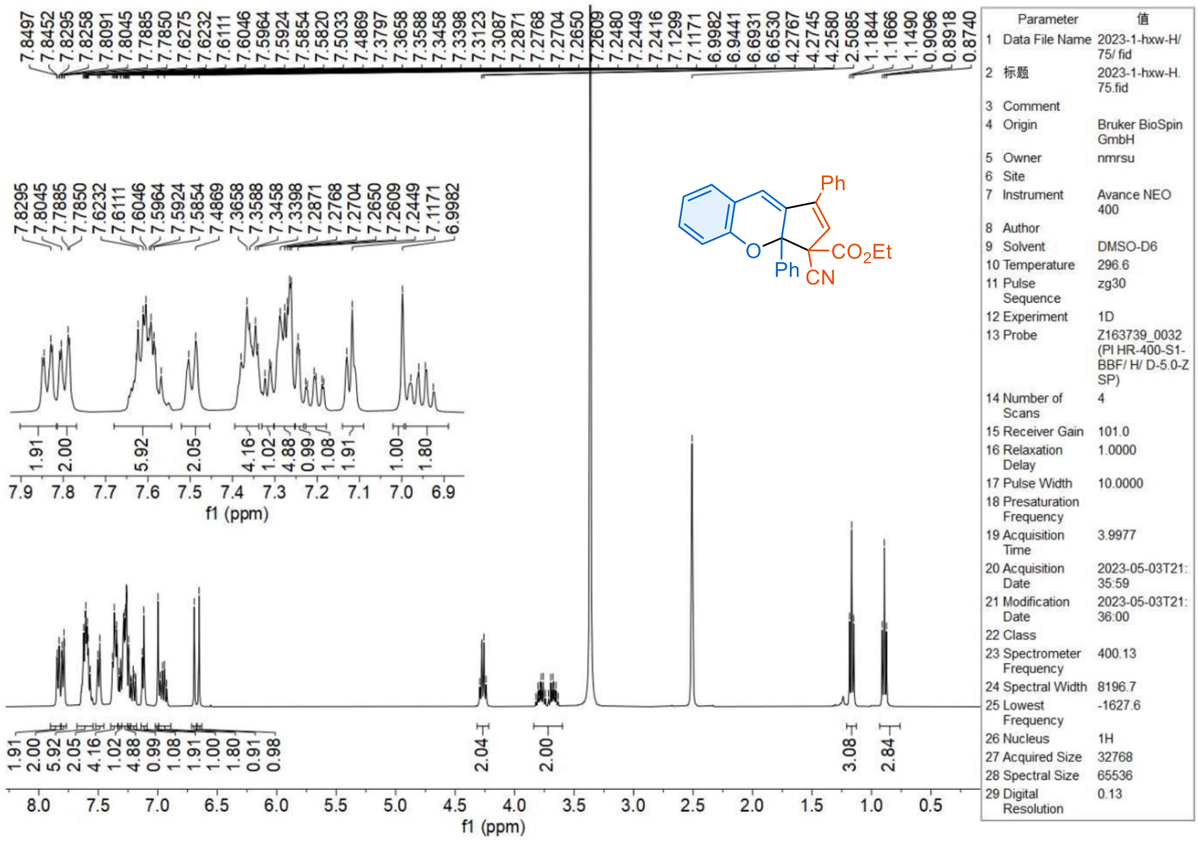

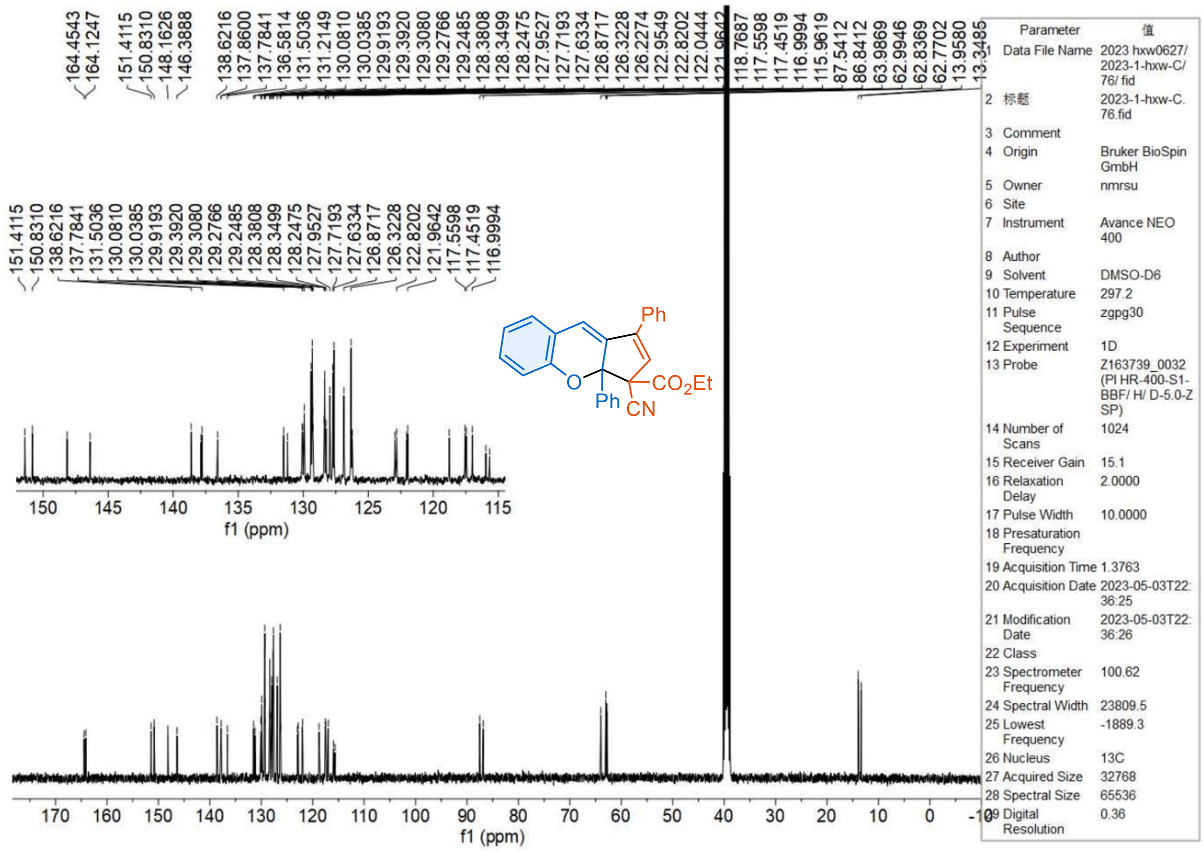

Isopropyl 3-cyano-1,3a-diphenyl-3,3a-dihydrocyclopenta[*b*]chromene-3-carboxylate (product 5c)

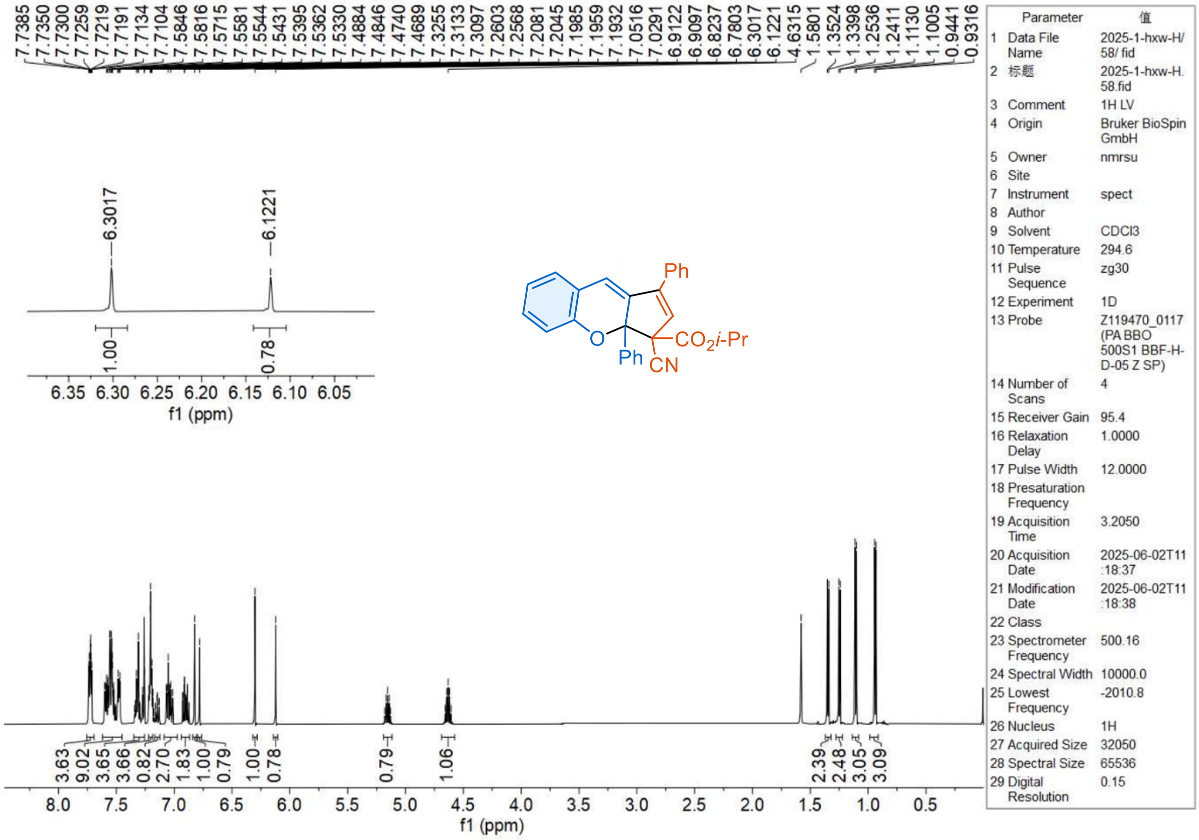

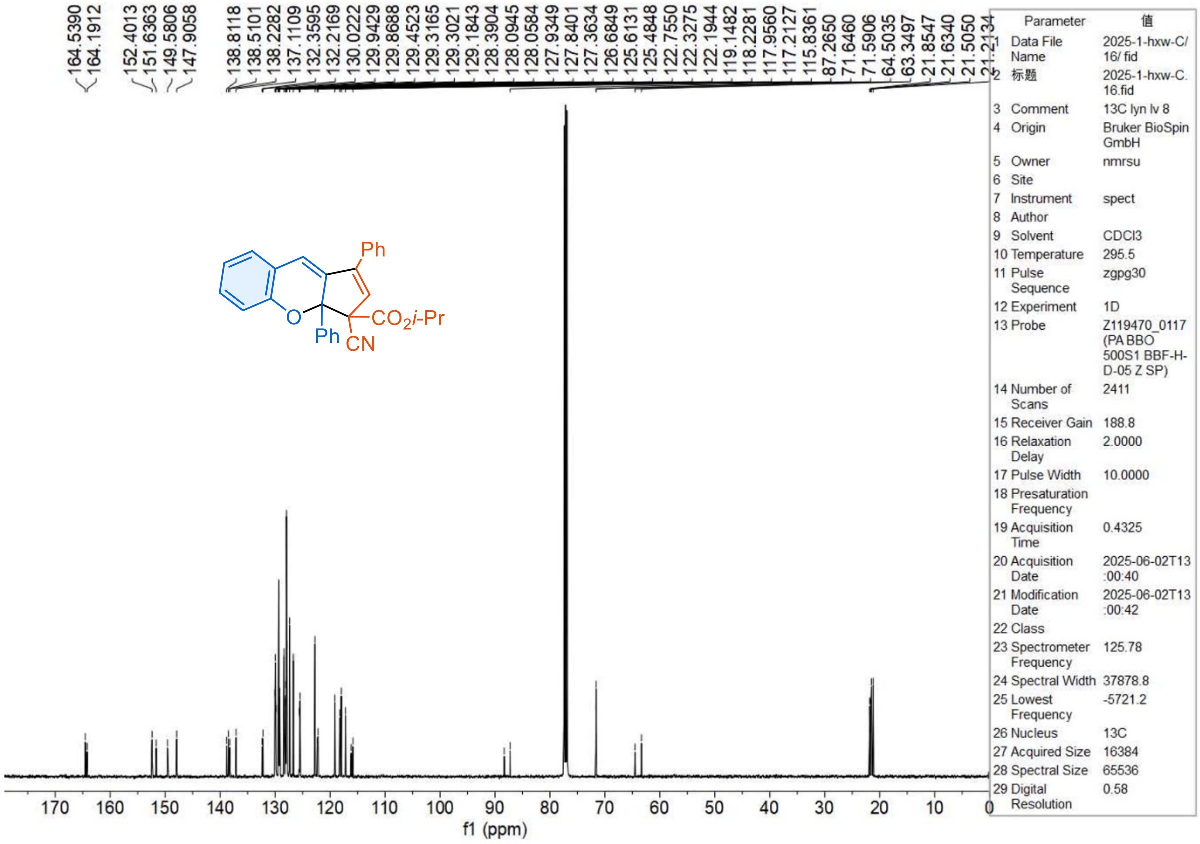

2-Methyl-1,3a-diphenylcyclopenta[*b*]chromene-3,3(3a*H*)-dicarbonitrile (product 7)

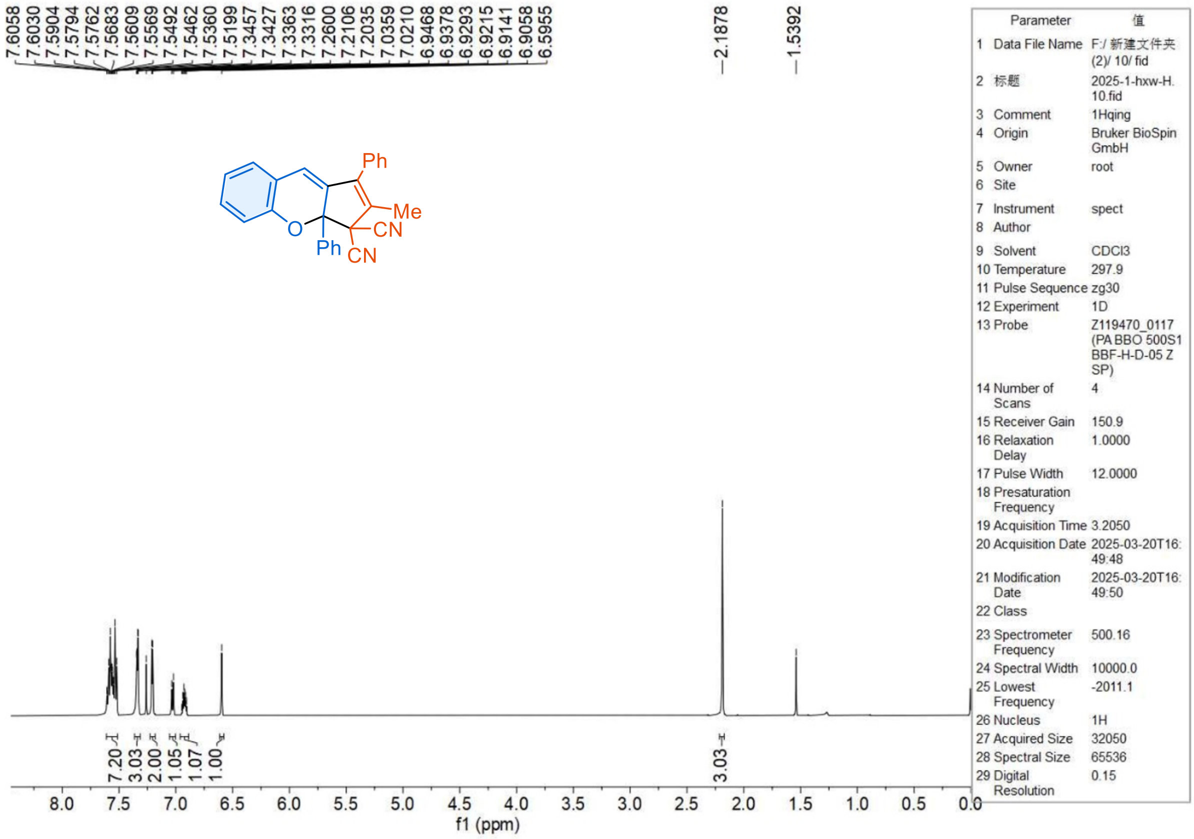

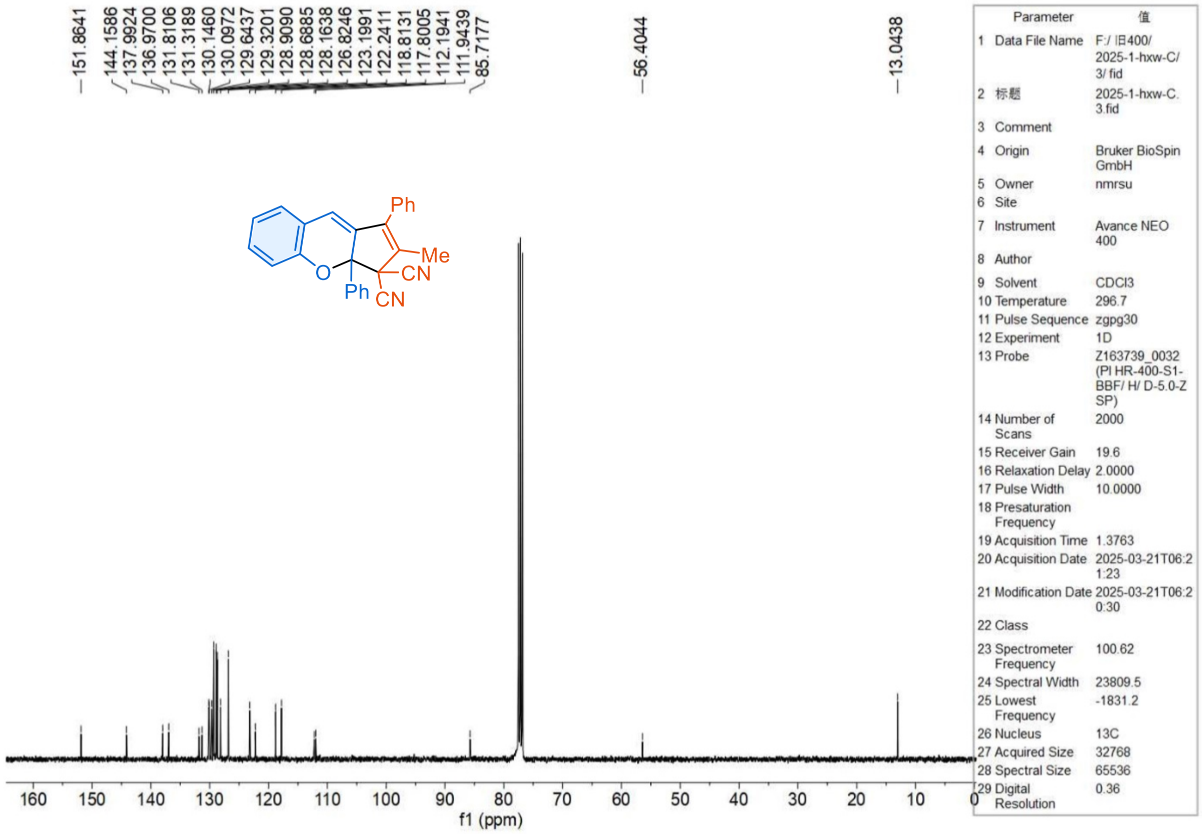

2,9-Dibromo-1,3a-diphenylcyclopenta[*b*]chromene-3,3(3a*H*)-dicarbonitrile (product 8)

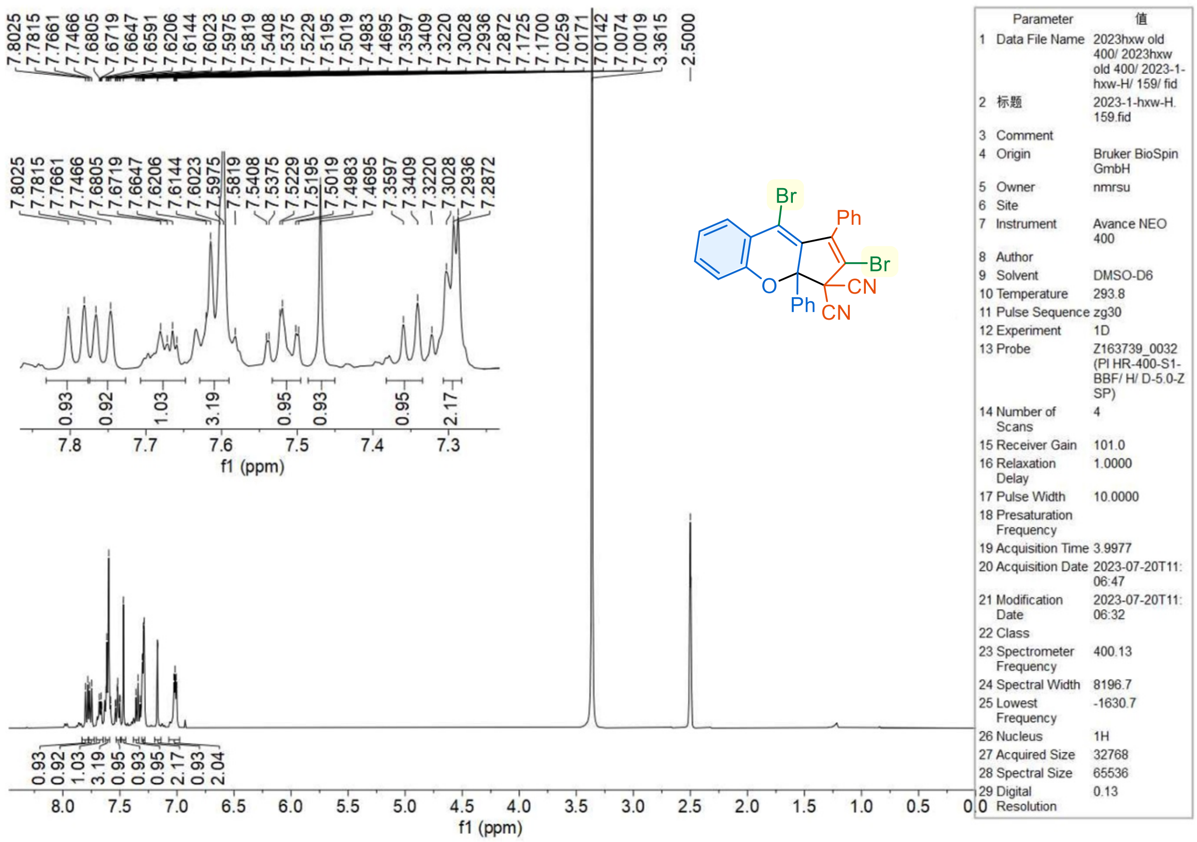

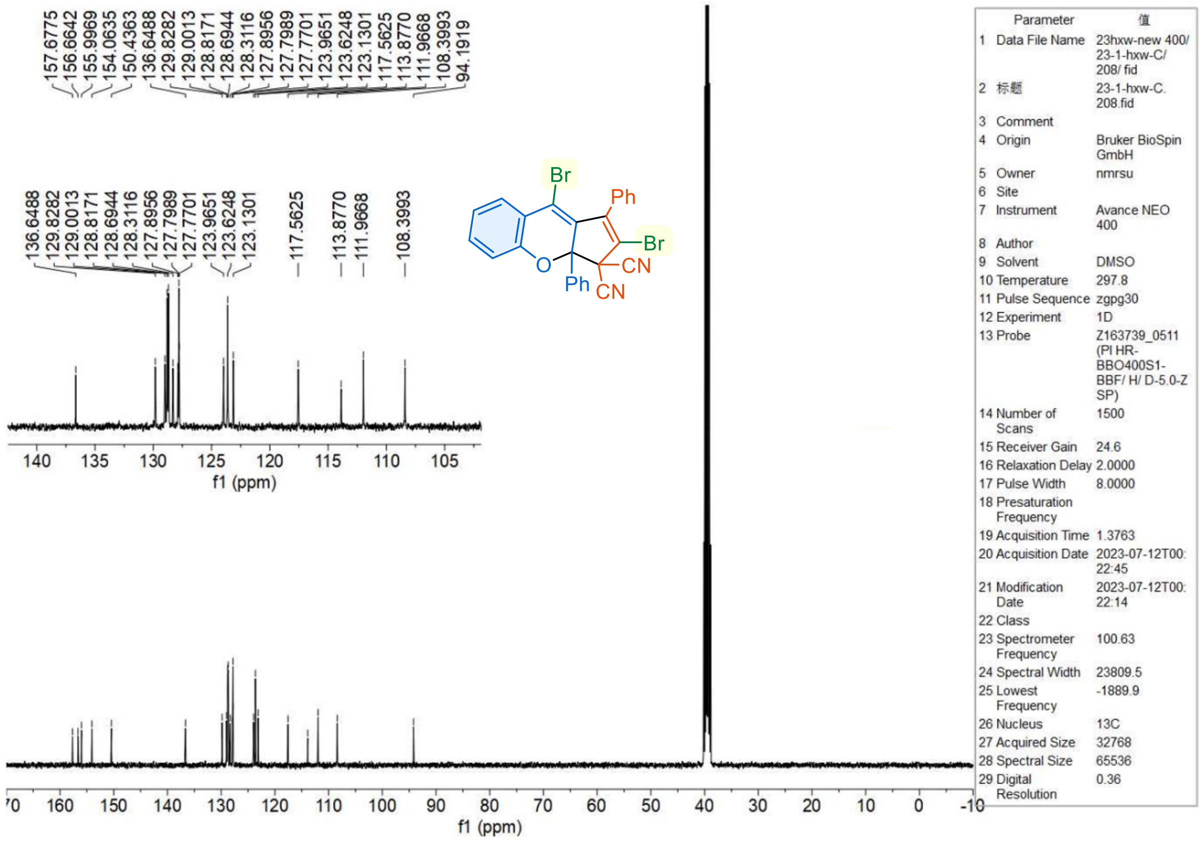

2-Bromo-9-hydroxy-1,3a-diphenyl-9,9a-dihydrocyclopenta[*b*]chromene-3,3(3a*H*)-dicarbonitrile (product 9)

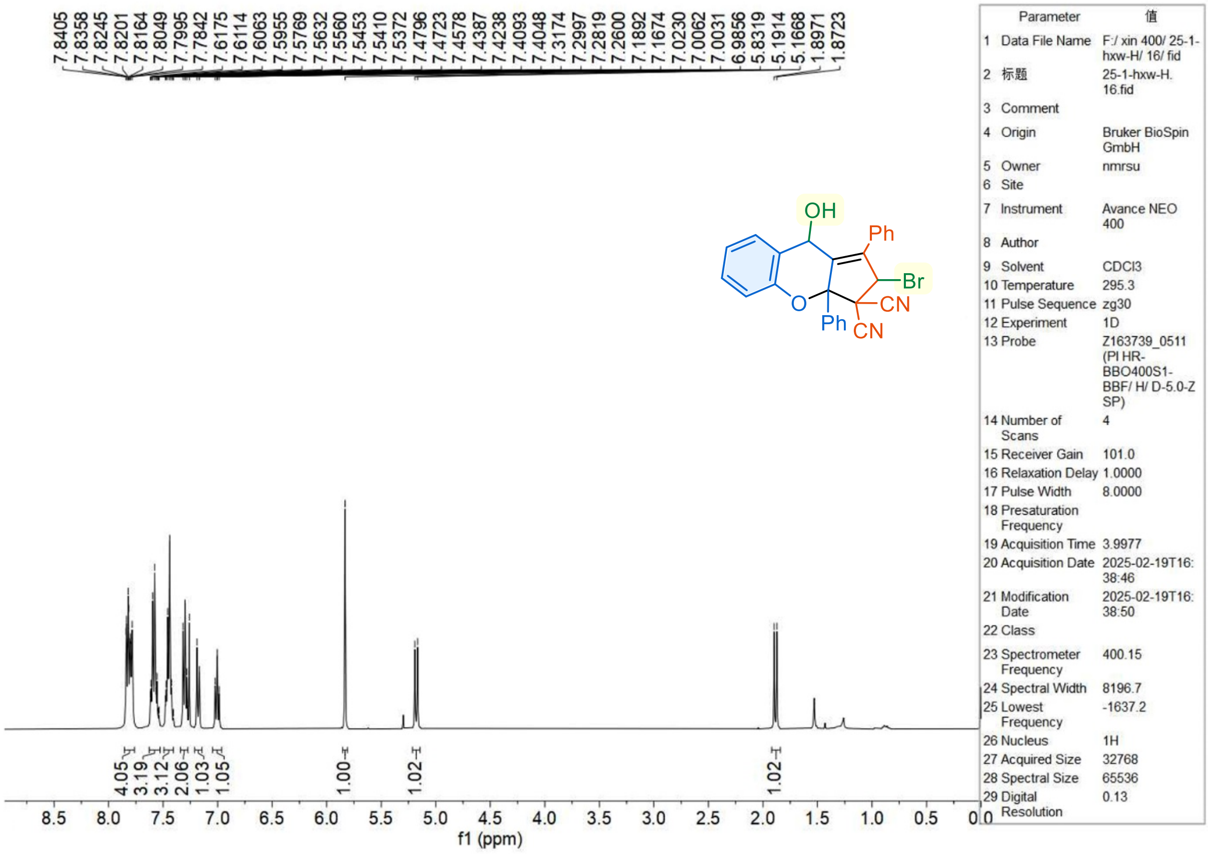

	Parameter	值
1	Data File Name	F:/ xin 400/ 25-1-hxw-H/ 16/ fid
2	标题	25-1-hxw-H.16.fid
3	Comment	
4	Origin	Bruker BioSpin GmbH
5	Owner	nmrsu
6	Site	
7	Instrument	Avance NEO 400
8	Author	
9	Solvent	CDCl3
10	Temperature	295.3
11	Pulse Sequence	zg30
12	Experiment	1D
13	Probe	Z163739_0511 (PI HR-BBO400S1-BBF/ H/ D-5.0-Z SP)
14	Number of Scans	4
15	Receiver Gain	101.0
16	Relaxation Delay	1.0000
17	Pulse Width	8.0000
18	Presaturation Frequency	
19	Acquisition Time	3.9977
20	Acquisition Date	2025-02-19T16:38:46
21	Modification Date	2025-02-19T16:38:50
22	Class	
23	Spectrometer Frequency	400.15
24	Spectral Width	8196.7
25	Lowest Frequency	-1637.2
26	Nucleus	1H
27	Acquired Size	32768
28	Spectral Size	65536
29	Digital Resolution	0.13

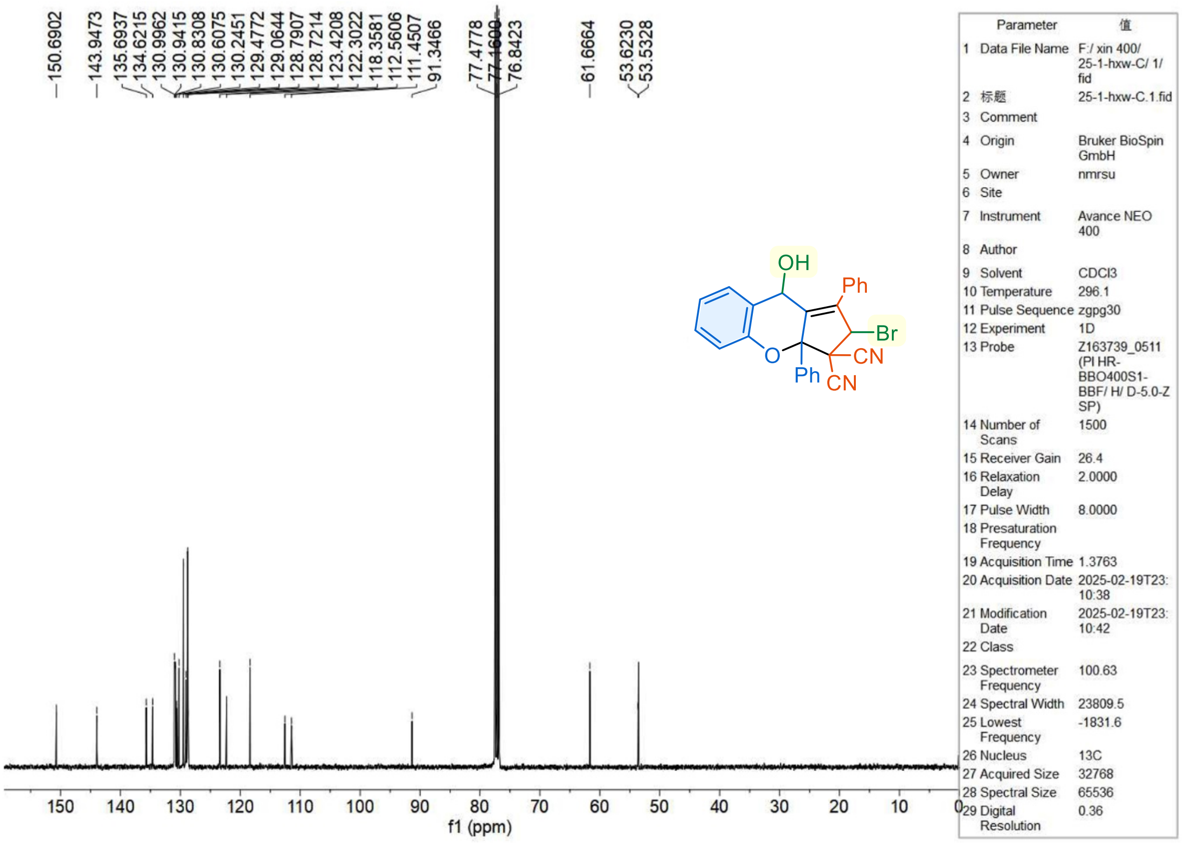

	Parameter	值
1	Data File Name	F:/ xin 400/ 25-1-hxw-C/ 1/ fid
2	标题	25-1-hxw-C.1.fid
3	Comment	
4	Origin	Bruker BioSpin GmbH
5	Owner	nmrsu
6	Site	
7	Instrument	Avance NEO 400
8	Author	
9	Solvent	CDCl3
10	Temperature	296.1
11	Pulse Sequence	zgpg30
12	Experiment	1D
13	Probe	Z163739_0511 (PI HR-BBO400S1-BBF/ H/ D-5.0-Z SP)
14	Number of Scans	1500
15	Receiver Gain	26.4
16	Relaxation Delay	2.0000
17	Pulse Width	8.0000
18	Presaturation Frequency	
19	Acquisition Time	1.3763
20	Acquisition Date	2025-02-19T23:10:38
21	Modification Date	2025-02-19T23:10:42
22	Class	
23	Spectrometer Frequency	100.63
24	Spectral Width	23809.5
25	Lowest Frequency	-1831.6
26	Nucleus	13C
27	Acquired Size	32768
28	Spectral Size	65536
29	Digital Resolution	0.36

4-(2-Benzyl-5-phenylfuran-3-yl)phenol (product 13)

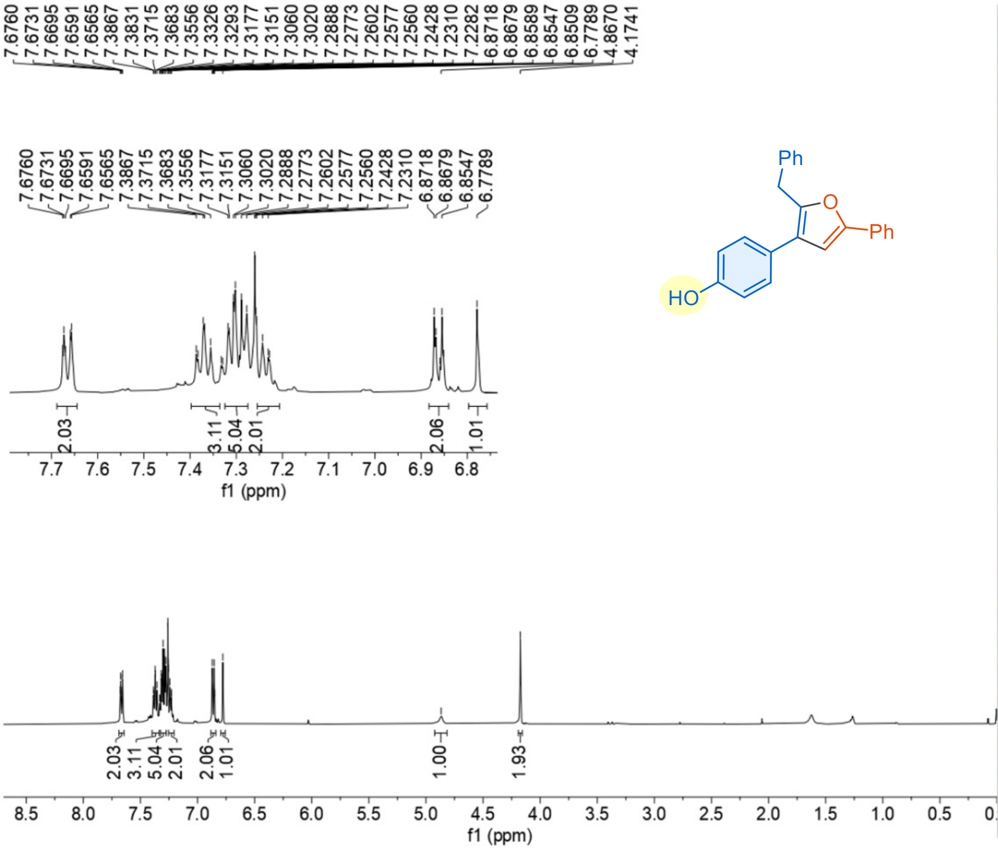

Parameter	值
1 Data File Name	500MHz/ 60/ fid
2 标题	2024-1-hxw-H. 60.fid
3 Comment	1H
4 Origin	Bruker BioSpin GmbH
5 Owner	root
6 Site	
7 Instrument	spect
8 Author	
9 Solvent	CDCl3
10 Temperature	294.0
11 Pulse Sequence	zg30
12 Experiment	1D
13 Probe	Z119470_0117 (PA BBO 500S1 BBF-H-D-05 Z SP)
14 Number of Scans	4
15 Receiver Gain	188.8
16 Relaxation Delay	2.0000
17 Pulse Width	12.0000
18 Presaturation Frequency	
19 Acquisition Time	2.0447
20 Acquisition Date	2024-10-23T17:26:58
21 Modification Date	2024-10-23T17:27:00
22 Class	
23 Spectrometer Frequency	500.16
24 Spectral Width	8012.8
25 Lowest Frequency	-1017.8
26 Nucleus	1H
27 Acquired Size	16384
28 Spectral Size	65536
29 Digital Resolution	0.12

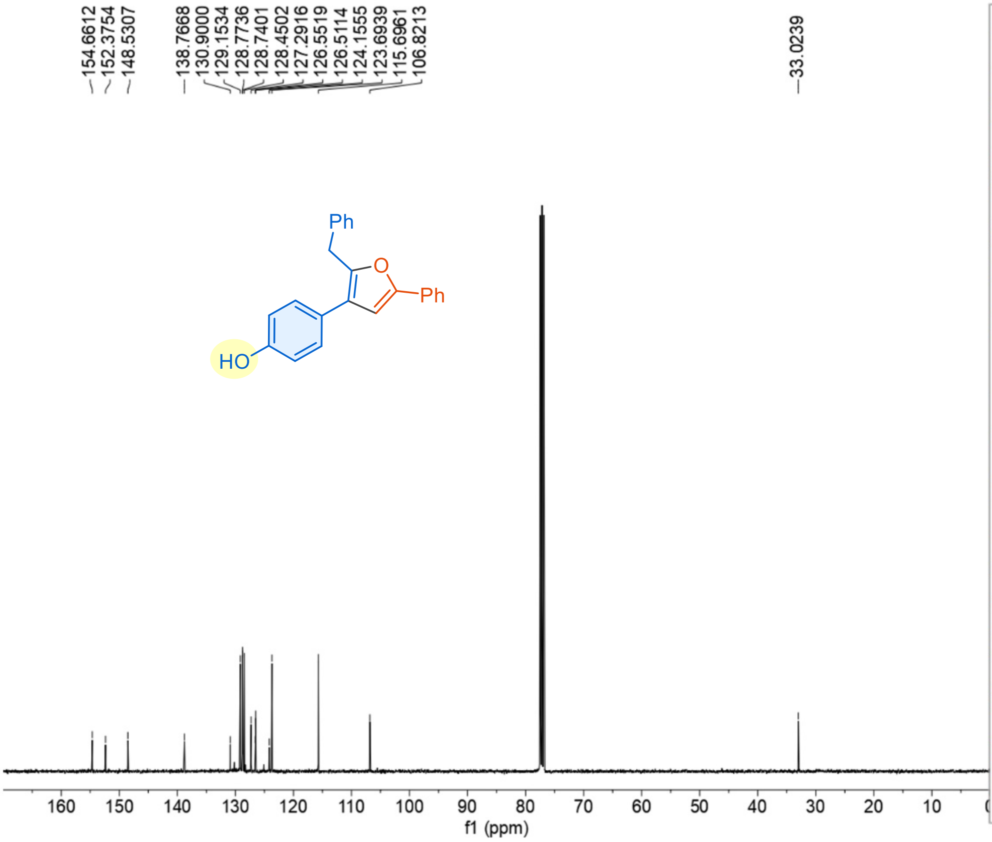

Parameter	值
1 Data File Name	F:/ jiu 400/ 78/ fid
2 标题	2024-2-hxw-C. 78.fid
3 Comment	
4 Origin	Bruker BioSpin GmbH
5 Owner	nmrsu
6 Site	
7 Instrument	Avance NEO 400
8 Author	
9 Solvent	CDCl3
10 Temperature	293.6
11 Pulse Sequence	zgpg30
12 Experiment	1D
13 Probe	Z163739_0032 (PI HR-400-S1-BBF/ H/ D-5.0-Z SP)
14 Number of Scans	2000
15 Receiver Gain	34.8
16 Relaxation Delay	2.0000
17 Pulse Width	10.0000
18 Presaturation Frequency	
19 Acquisition Time	1.3763
20 Acquisition Date	2024-10-26T05:22:04
21 Modification Date	2024-10-26T05:21:06
22 Class	
23 Spectrometer Frequency	100.62
24 Spectral Width	23809.5
25 Lowest Frequency	-1722.0
26 Nucleus	13C
27 Acquired Size	32768
28 Spectral Size	65536
29 Digital Resolution	0.36

15. Proposed Mechanisms for the Generation of Compound 9 and 13

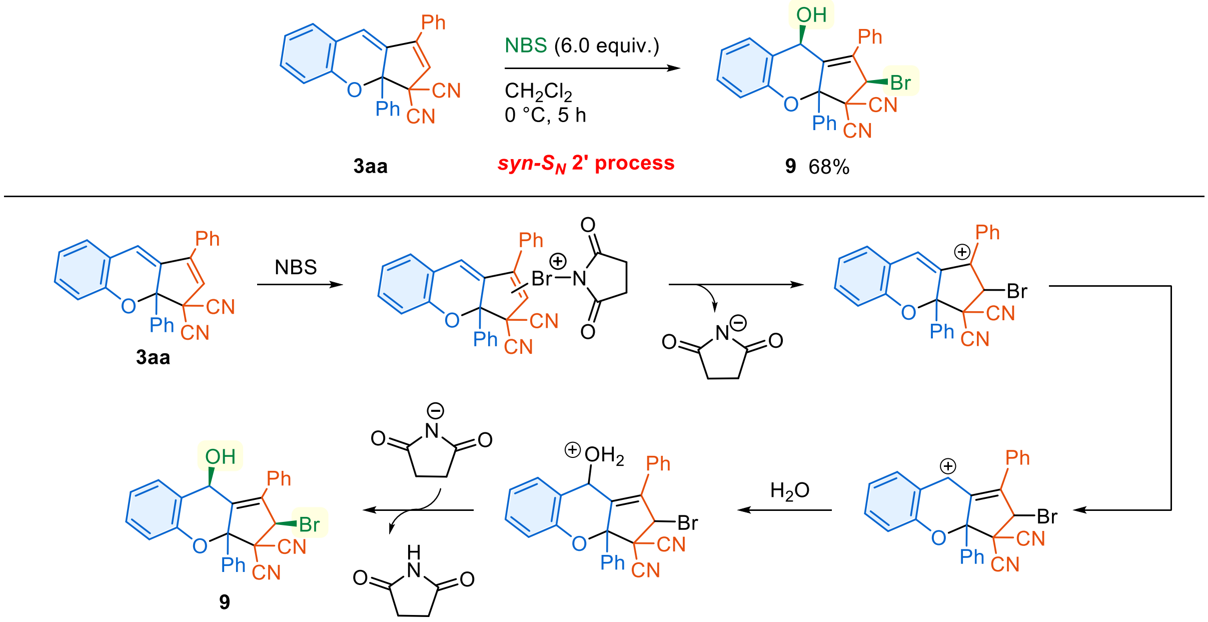

Scheme S1. Proposed mechanism for the formation of compound **9**

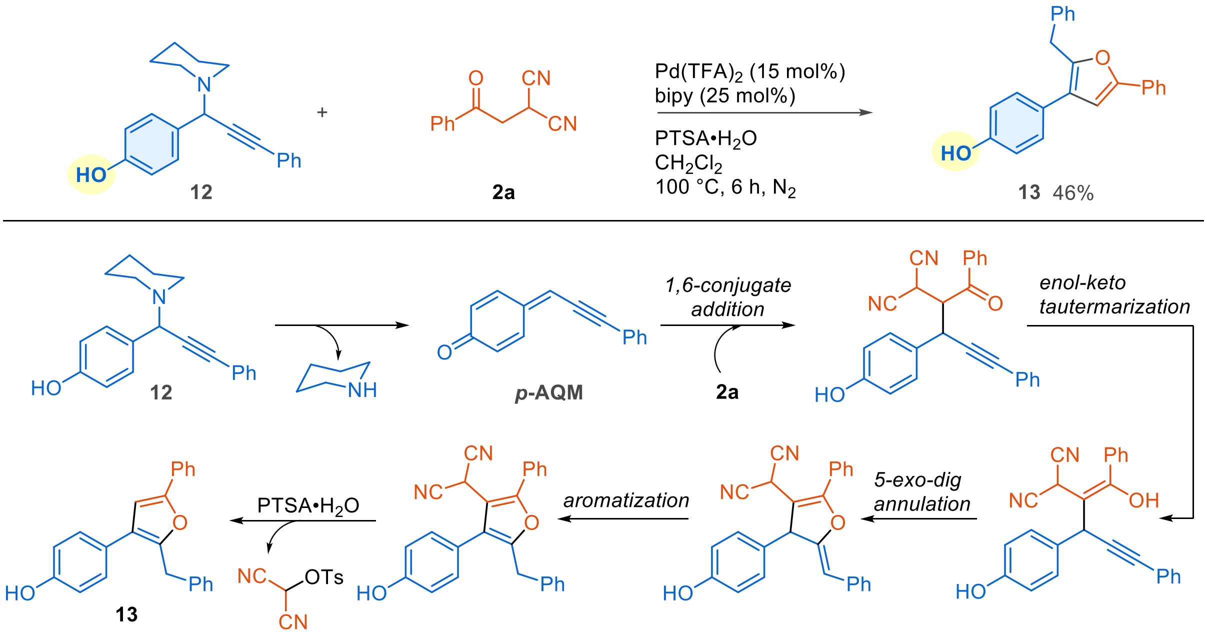

Scheme S2. Proposed mechanism for the formation of compound **13**

16. GC-MS Spectra for Mechanistic Investigations

Intermediate F

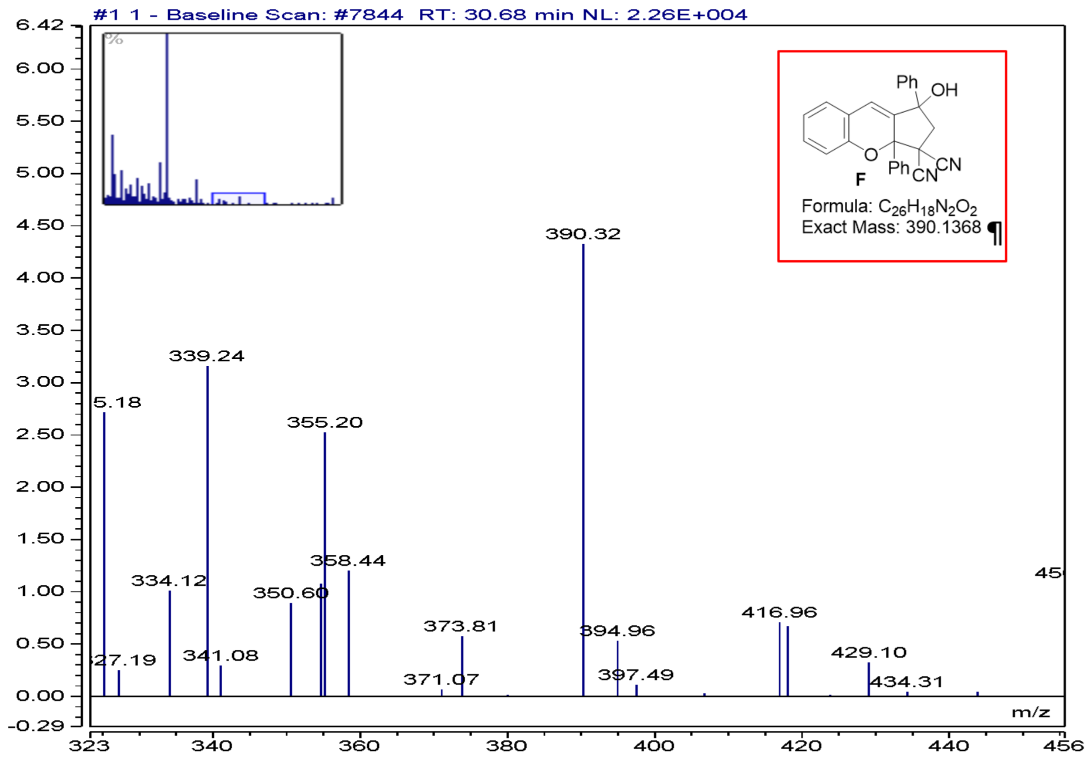

Figure S5. GC-MS Spectra of possibly intermediate **F**

17. Photophysical Properties of Product 3 and Their Derivatives

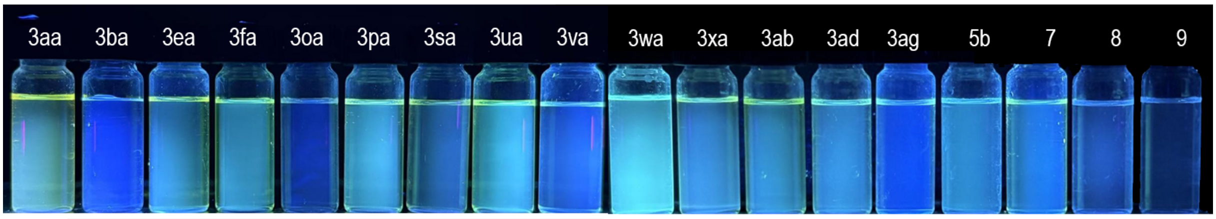

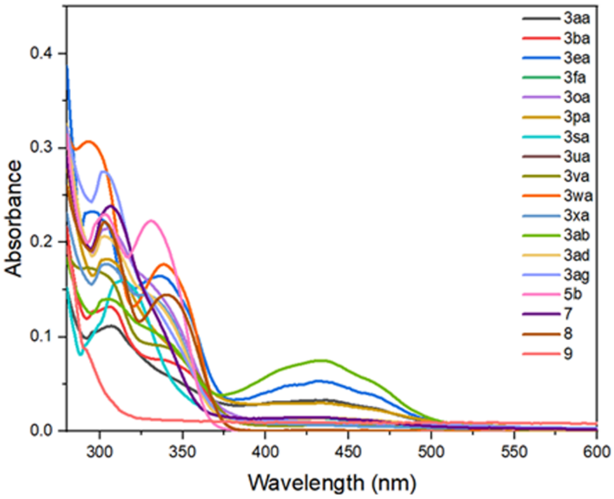

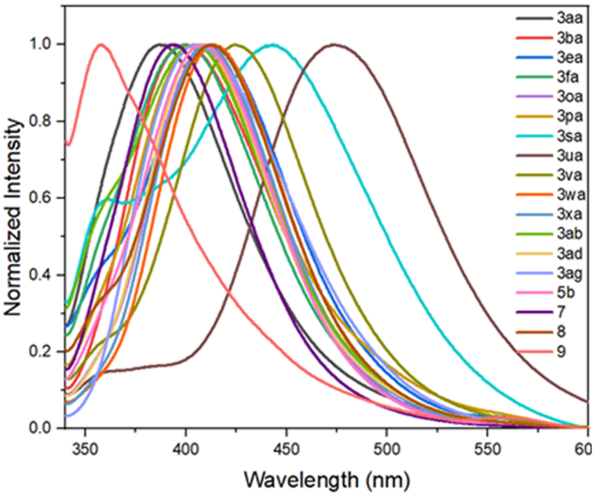

Table S6. Photophysical Properties for product **3** and their derivatives

entry	product	λ_{abs} (nm)[a]	ε ($L \cdot mol^{-1} \cdot cm^{-1}$)[b]	λ_{em} (nm)[c]	Stokes shift (nm)
1	**3aa**	328	7350	387	59
2	**3ba**	340	3753	400	60
3	**3ea**	336	8225	413	77
4	**3fa**	334	6115	398	64
5	**3oa**	333	7590	410	77
6	**3pa**	342	4693	405	63
7	**3sa**	315	7931	443	128
8	**3ua**	331	11145	474	143
9	**3va**	338	8839	425	87
10	**3wa**	344	4313	414	70
11	**3xa**	329	7219	409	80
12	**3ab**	334	5208	405	71
13	**3ad**	336	6715	408	72
14	**3ag**	335	6418	405	70
15	**5b**	331	11145	407	76
16	**7**	334	6919	395	61
17	**8**	341	7239	412	71
18	**9**	-	-	358	-

[a]Wavelength in absorption spectra were measured in dimethyl sulfoxide solutions (ca. 2 × 10^{-5} M) at room temperature. [b]ε refers to the molar extinction coefficient. [c]Wavelength in emission spectra were recorded at

λ_{exc} = 320 nm.

18. References

(1) Armarego, W. L. F. *Purification of Laboratory Chemicals*, 8th ed.; Butterworth-Heinemann: Oxford, U.K., **2017**.

(2) He, X.; Xie, M.; Li, R.; Choy, P. Y.; Tang, Q.; Shang, Y.; Kwong, F. Y. A Organocatalytic Approach for Assembling Flavanones via a Cascade 1,4-Conjugate Addition/oxa-Michael Addition between Propargylamines with Water. *Org. Lett.* **2020**, *22*, 4306−4310.

(3) Rakshit, A.; Dhara, H. N.; Sahoo, A. K.; Alam, T.; Patel, B. K. Visible-Light-Mediated Synthesis of Thio-Functionalized Pyrroles. *Org. Lett.* **2022**, *24*, 3741−3746.

(4) Hu, W.; He, X.; Zhou, T.; Zuo, Y.; Zhang, S.; Yang, T.; Shang, Y. Construction of isoxazolone-fused phenanthridines via Rh-catalyzed cascade C–H activation/cyclization of 3-arylisoxazolones with cyclic 2-diazo-1,3-diketones. *Org. Biomol. Chem.* **2021**, *19*, 552−556.
